# PCBP1/2 and TDP43 Function as NAT10 Adaptors to Mediate mRNA ac^4^C Formation in Mammalian Cells

**DOI:** 10.1002/advs.202400133

**Published:** 2024-11-18

**Authors:** Zhi‐Yan Jiang, Yu‐Ke Wu, Zuo‐Qi Deng, Lu Chen, Yi‐Min Zhu, Yuan‐Song Yu, Hong‐Bo Wu, Heng‐Yu Fan

**Affiliations:** ^1^ Zhejiang Key Laboratory of Precise Protection and Promotion of Fertility Assisted Reproduction Unit Department of Obstetrics and Gynecology Sir Run Run Shaw Hospital School of Medicine Zhejiang University Hangzhou 310016 China; ^2^ MOE Key Laboratory for Biosystems Homeostasis and Protection and Innovation Center for Cell Signaling Network Life Sciences Institute Zhejiang University Hangzhou Zhejiang 310058 China; ^3^ Department of Reproductive Endocrinology, Women's Hospital, School of Medicine Zhejiang University Hangzhou 310002 China; ^4^ Savaid Stomatology School Hangzhou Medical College Hangzhou 310053 China; ^5^ Department of Reproductive Medicine Qinzhou Maternal and Child Health Care Hospital Qinzhou 535099 China; ^6^ Center for Biomedical Research Shaoxing Institute Zhejiang University Shaoxing 312000 China

**Keywords:** mRNA modification, N‐4‐acetylcytidine, posttranscriptional regulation, RNA binding protein, testis, transcriptome

## Abstract

Massive numbers of modified bases in mRNAs sculpt the epitranscriptome and play vital roles in RNA metabolism. The only known acetylated RNA modification, N‐4‐acetylcytidine (ac^4^C), is highly conserved across cell types and among species. Although the GCN5‐related acetyltransferase 10 (NAT10) functions as an ac^4^C writer, the mechanism underlying the acetylation process is largely unknown. In this study, the NAT10/PCBP/TDP43 complex mediated mRNA ac^4^C formation in mammalian cells is identified. RNA‐binding proteins (RBPs) are identified, affiliated with two different families, poly(rC)‐binding protein 1/2 (PCBP1/2) and TAR DNA binding protein 43 (TDP43), as NAT10 adaptors for mRNA tethering and substrate selection. Knockdown of the adaptors resulted in decreased mRNA acetylation abundance in HEK293T cells and ablated cytidine‐rich ac^4^C motifs. The adaptors also affect the ac^4^C sites by recruiting NAT10 to their binding sequences. The presence of the NAT10/PCBP/TDP43 complex in mouse testes highlights its potential physiological functions in vivo. These findings reveal the composition of the mRNA ac^4^C writer complex in mammalian cells and expand the knowledge of mRNA acetylation and ac^4^C site preferences.

## Graphical abstract

Poly(rC)‐binding protein 1/2 (PCBP1/2) and TAR DNA binding protein 43 (TDP43) function as NAT10 adaptors for mRNA acetylation in mammalian cells. These adaptors are responsible for substrate tethering and selection. Loss of the adaptors results in decreased mRNA acetylation abundance and ablated cytidine‐rich ac^4^C motifs. The presence of the NAT10/PCBP/TDP43 complex is conserved among cell types and across species.

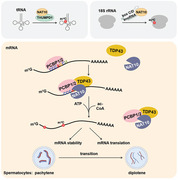

## 1 Introduction

Various RNA modifications sculpt the epitranscriptome and regulate multiple post‐transcriptional metabolic processes, including splicing,^[^ ^1^ ^]^ translocation,^[^ ^2^ ^]^ translation,^[^ ^3^ ^]^ and degradation.^[^ ^4^ ^]^ To date, more than 170 types of modifications have been identified in eukaryotic mRNAs.^[^ ^5^ ^]^ Among these, N‐4‐acetylcytidine (ac^4^C) is the only identified acetylated modification. ac^4^C exists in three categories of RNAs, including transfer RNA (tRNA), ribosomal RNA (rRNA), and mRNA, and is abundant across species.^[^ ^6^ ^]^ Transcriptome‐wide ac^4^C mapping of mammalian mRNAs has revealed that the modification distributes along the whole transcript with high enrichment in the 5′‐untranslated region (5′‐UTR) and in the vicinity of the transcription start site (TSS).^[^ ^6c^ ^]^ The ac^4^C residues within the coding sequence (CDS) of a transcript have a wobble site preference in codons, which further promotes codon decoding and enhances the stability of the host mRNA. The ac^4^C residues within the 5′‐UTR bivalently affect translation initiation in a position‐dependent manner, as those residing in the Kozak sequence around the annotated translation initiation site (TIS) hamper translation initiation, whereas those located downstream of the non‐annotated TIS promote non‐canonical translation initiation.^[^ ^7^ ^]^ Recently, it has emerged that ac^4^C might be a diagnostic indicator, as increased ac^4^C abundance has been detected in tumors and in patients suffering from various diseases, including gastric cancer and systemic lupus erythematosus.^[^ ^8^ ^]^ Although the underlying mechanism remains elusive, acetylation of specific mRNAs can promote cell proliferation, tumorigenesis, and cancer metastasis, and influence cell metabolism. This strong relationship between mRNA acetylation and diseases has driven research into the potential pathogenic role of mRNA ac^4^C and its physiological function.

To date, GCN5‐related N‐acetyltransferase 10 (NAT10) is the only known ac^4^C‐catalyzing enzyme.^[^ ^6a–c^ ^]^ Studies have found that various mRNAs are acetylated by NAT10 in diverse cell types and across species, which further upregulates their protein levels, including *COL5A1* in gastric cancer cell metastasis^[^ ^8a^ ^]^ and *AHNAK* in DNA damage repair response against chemotherapy in bladder cancer.^[^ ^9^ ^]^ The lack of *NAT10* hinders cell proliferation and migration but does not affect cell viability.^[^ ^6^, ^10^ ^]^ Germ cell‐specific knockout of *Nat10* mouse resulted in sterility in both genders. *Nat10* ablation in spermatocytes causes failure of meiotic entry, abnormal chromosomal behavior, DNA damage repair defects, and a pachytene stage arrest.^[^ ^11^ ^]^ Similarly, oocytes from *Nat10^fl/fl^;Stra8‐*Cre mice were arrested at the pachytene‐like stage. NAT10 guards the CCR4‐NOT complex‐driven maternal mRNA removal, further promoting meiotic maturation and early embryogenesis.^[^ ^12^ ^]^

However, the mechanisms underlying mRNA acetylation remain unclear. Multiple domains have been found within the NAT10 protein, including a DUF1726 domain, two tandem domains that function as an ATPase and a helicase, an N‐acetyltransferase domain, and a predicted tRNA‐binding domain from the N‐terminus to the C‐terminus.^[^ ^9^, ^13^ ^]^ Despite the presence of a tRNA‐binding domain, NAT10 requires THUMP domain‐containing 1 (THUMPD1) for tRNA acetylation.^[^ ^6b^ ^]^ Similarly, its homolog, Kre33 in yeast, requires Tan1, the THUMPD1 homolog in yeast. In 18S rRNA acetylation, a vertebrate‐specific box C/D snoRNA, U13, is responsible for bridging NAT10 with the substrate RNA. However, the construction mechanism of the mRNA N‐acetyltransferase complex and potential adaptors that facilitate NAT10‐mRNA targeting remain elusive. Whether such adaptors affect substrate selection and ac^4^C site specificity, and whether the potential complex is conserved among cell types and across species remain to be determined.

In this study, we identified three mRNA‐binding proteins, PCBP1/2 and TDP43 (encoded by *TARDBP* gene), as NAT10 adaptors for mRNA acetylation in mammalian cells. The PCBP/TDP43/NAT10 complex specifically mediates mRNA ac^4^C formation and does not influence ac^4^C abundance in non‐poly(A) RNA, total RNA, or other mRNA modifications. A lack of *PCBP1/2* or *TDP43* results in a decrease or loss of acetylation of mRNAs. These proteins may partially account for mRNA substrate selection. Furthermore, we demonstrated that the conjugation of PCBP/TDP43/NAT10 is conserved across cell types and among species. These results reveal the molecular mechanism of mRNA ac^4^C formation in mammalian cells and clarify the functions of PCBP1/2 and TDP43 as mRNA adaptors in mRNA acetylation.

## 2 Results

### 2.1 Identification of PCBP1, PCBP2, and TDP43 as NAT10‐Associated Proteins

**Figure 1:**
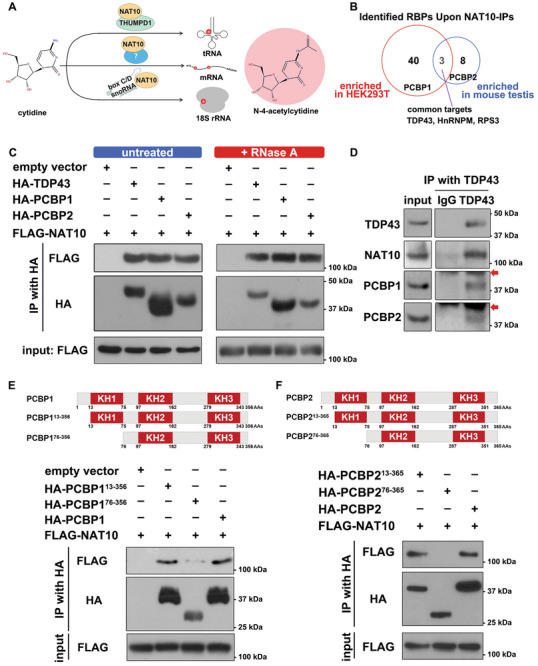
Identification and confirmation of TDP43 and PCBP1/2 as NAT10 adaptors. A) Scheme of N‐4‐acetylcytidine formation in mammalian RNAs. THUMPD1 and box C/D snoRNAs facilitate NAT10 in tRNA and 18S rRNA ac^4^C production, respectively. In mRNA ac^4^C formation, the presumed adaptor remains unknown. B) Comparison of NAT10 interactomes in HEK293T cells and mouse testis. HEK293T cells expressing N‐terminus FLAG‐tagged NAT10 and wild‐type (WT) mouse testis were subjected to affinity purification with anti‐FLAG and anti‐NAT10 antibody, respectively, and mass spectrometry analysis. RNA‐binding proteins (RBPs) including PCBP1, PCBP2, and TDP43 presented affinity toward NAT10.^[^ ^15^ ^]^ C) Co‐immunoprecipitation (co‐IP) results showing the interactions between FLAG‐tagged NAT10 and HA‐tagged TDP43, PCBP1, and PCBP2, which were resistant to RNase A digestion. D) Endogenous immunoprecipitation (endo‐IP) results showing spontaneous coordination of TDP43, NAT10, and PCBP1/2 in 293T cells. Anti‐TDP43 antibody was applied in endo‐IP. Arrows indicated the beads band. E‐F) Diagram of N‐terminal truncated PCBP1/2 constructs and validation of the interaction between NAT10 and WT or mutant PCBPs. Co‐IP results indicated that the hnRNP K homology (KH) 1 domain in PCBP1/2 was responsible for their conjugation with NAT10.

Formation of ac^4^C in tRNA and 18S rRNA by NAT10 requires THUMPD1 and U13 box C/D snoRNA, respectively (**Figure** **1**A).^[^ ^6b^ ^]^ However, little is known about the co‐factors involved in mRNA ac^4^C production. To identify candidates that serve as mRNA adaptors in the ac^4^C acetylase complex in mammalian cells, we used affinity purification to pull down FLAG‐tagged NAT10, the known ac^4^C writer from HEK293T cells (Figure S1A, Supporting Information). 293T transfected with the empty vector carrying FLAG was applied as a negative control. Subsequent mass spectrometry analysis showed a high correlation within groups, identifying 535 proteins in the FLAG‐NAT10 interactome (Figure S1B,C, Supporting Information). To clarify whether a similar group of proteins exist in mRNA acetylation, the affinity purification assay was also performed to pull down endogenous NAT10 from wild‐type (WT) mouse testis, with IgG serving as the negative control (Figure S1D, Supporting Information). Within the interactome, 43 and 11 RNA binding proteins (RBPs) were identified in 293T cells and mouse testis, and 3 proteins including TDP43 (TAR DNA binding protein 43, encoded by *TARDBP* gene, labeled as *TDP43* as follows), HnRNP M (heterogeneous ribonuclear protein M) and RPS3 (ribosomal protein S3) were detected as common targets (Figure 1B). Meanwhile, PCBP1 (Poly (rC)‐binding protein 1, also termed HNRNPE1) and its homolog, PCBP2 (also termed *HNRNPE2*), were defined as NAT10 interactors in 293T cells and mouse testis, respectively. PCBP2 is highly conserved in peptide sequence and protein structure compared to PCBP1, which is previously reported to spontaneously form dimers with PCBP1, and was also subjected to analysis (Figure S1E, Supporting Information).^[^ ^14^ ^]^ In addition to its primary localization in nucleolus, NAT10 also expressed in the nucleoplasm in 293T cells, where TDP43 and PCBP1 mainly dispersed and form foci (Figure S2A, Supporting Information). In mouse testis, immunofluorescence (IF) analyses indicated that PCBP1/2 and TDP43 were expressed in spermatocytes in meiotic prophase I, whereas in pachytene‐stage spermatocytes, PCBP1 and TDP43 formed plaque‐like signals similar to NAT10 (Figure S2B, Supporting Information). IF results also presented the co‐existence of PCBP1/2, TDP43, and NAT10 in mouse nucleus, with a preferred localization of NAT10 around the nucleolus in mouse oocytes (Figure S2C, Supporting Information). The interactions between TDP43, PCBP1, PCBP2, and NAT10 were validated by co‐immunoprecipitation (co‐IP; Figure 1C). Furthermore, the co‐IP results accompanied by RNase A treatment indicated that the interaction affinity with NAT10 was independent of the RNAs. Endogenous immunoprecipitation (endo IP) using a TDP43 antibody further confirmed TDP43 interaction with NAT10, which also bound to PCBP1 and PCBP2 in 293T cells (Figure 1D).

Using PCBP1 constructs carrying three tandem separated sections of full‐length PCBP1, PCBP1^1–96^ (96 amino acid residues, 96 AAs), PCBP1^97–278^ (182 AAs), and PCBP1^279–356^ (78 AAs), we found that the interaction between PCBP1 and NAT10 depended on the 96 amino acids at the N‐terminus of PCBP1 (Figure S2D, Supporting Information). Moreover, PCBP1 contains three hnRNP K homology domains (KH domains, KH1‐3, respectively), while its KH1 domain resides from threonine 13 (T13) to isoleucine 75 (I75).^[^ ^14a^ ^]^ We further applied PCBP1 constructs with N‐terminal truncation (PCBP1^13–356^, truncated ahead of the KH1 domain and PCBP1^76–356^, with the KH1 domain deleted) and found that the interaction between PCBP1 and NAT10 relies on the KH1 domain in PCBP1 (Figure 1E). Based on the similarity between the PCBP1/2 proteins, these homologs shared 87.2% consensus positions and 81.1% identity positions, with 95.2% identity positions in their KH1 domains (Figure S1E, Supporting Information). Hence, we applied PCBP2 constructs with N‐terminal truncation (PCBP2^13–365^, truncated ahead of the KH1 domain and PCBP2^76–365^, with the KH1 domain deleted) and found that the KH1 domain in PCBP2 determined the interaction between PCBP2 and NAT10 (Figure 1F). Similarly, we verified whether the interaction between TDP43 and PCBP1 required the same region of interaction between NAT10 and PCBP1. Both N‐terminus‐truncated PCBP1 constructs could bind to TDP43, indicating that the binding affinity of PCBP1 to TDP43 relied on the C‐terminal region within PCBP1 (Figure S2E, Supporting Information). These results indicate that the KH1 domains of PCBP1 and PCBP2 are responsible for their interaction with NAT10, whereas the interaction between PCBP1 and TDP43, called the C‐terminus of PCBP1, and NAT10/PCBP/TDP43 conjugated together in vivo.

### 2.2 PCBP1/2 and TDP43 are Required for N‐4‐Acetylation on Cytidines In Vivo

**Figure 2:**
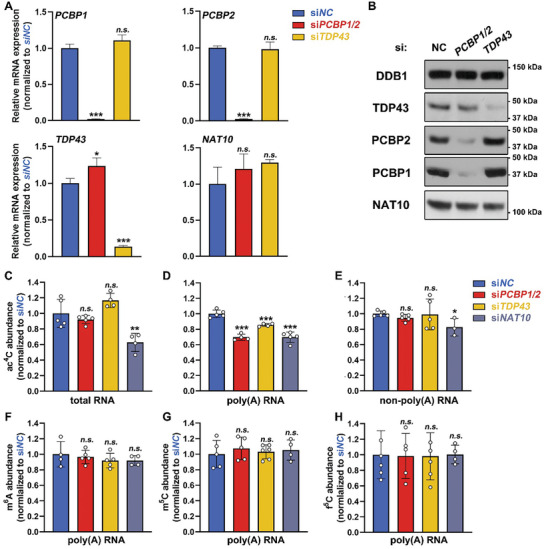
Depletion of PCBP1/2 and TDP43 resulted in decreased mRNA ac^4^C abundance in HEK293T cells. A) Comparison of the mRNA expression levels of *PCBP1/2, TDP43*, and *NAT10* between the control group (si*NC*) and upon *PCBP1/2* or *TDP43* knockdown (*siPCBP1/2* and *siTDP43*, respectively) by RT‐qPCR. Gene expression levels were normalized to *GAPDH*. Mean ± SEM, n = 3. *P*‐values were calculated using the two‐tailed Student's *t*‐test between the knockdown groups and si*NC* group. ***P < 0.001. *n.s*., not significant. B) Comparison of the protein expression levels of PCBP1/2, TDP43, and NAT10 between the control group (si*NC*) and *PCBP1/2* or *TDP43* knockdown groups. DDB1 was blotted as a loading control. C‐E) LC‐MS/MS detection of ac^4^C abundance (ac^4^C/C) in total RNA, poly(A) RNA, and non‐poly(A) RNA in the control group and in *PCBP1/2*, *TDP43*, or *NAT10* knockdown groups. The ac^4^C abundance was normalized to the si*NC* group in each RNA type. Mean ± SEM. *P < 0.05, **P < 0.01. F‐H) LC‐MS/MS detection of m^6^A, m^5^C, and f^5^C abundance (m^6^A/A, m^5^C/C, and f^5^C/C, respectively) in poly(A) RNA in the control group and upon *PCBP1/2*, *TDP43*, or *NAT10* knockdown. The indicated modification abundance was normalized to the control group. Mean ± SEM.

Considering the stability of the NAT10/PCBP/TDP43 complex, we first examined the expression level of each subunit upon *PCBP1/2* and *TDP43* depletion by siRNAs. Owing to sequence similarity and siRNA non‐specificity, *PCBP1/2* was depleted together (Figure S3A–C, Supporting Information). Negative control siRNA (*siNC*) was also applied. Reverse transcription followed by quantitative PCR (RT‐qPCR) and western blotting showed that the RNA and protein levels of PCBP1/2 were dramatically reduced, whereas the expression levels of TDP43 and NAT10 were slightly altered (**Figure** **2**A,B). Vice versa.

NAT10 has previously been reported to harbor an N‐4‐acetyltransferase and to serve as an acetylase in ac^4^C formation in mRNAs in multiple cell types.^[^ ^6^, ^8^, ^11^, ^16^ ^]^ However, PCBP1/2 and TDP43 do not contain any catalytic domains, and we speculated that they serve as NAT10 adaptors in mRNA acetylation. To confirm whether these potential NAT10 adaptors affect mRNA ac^4^C abundance, RNAs extracted from *siNC*, *siPCBP1/2*, and *siTDP43* 293T cells were subjected to HPLC‐MS/MS analysis. 293T cells with *NAT10* knockdown were served as a positive control, with its depletion efficiency validated (Figure S4L–M, Supporting Information). Poly(A) RNAs were isolated from the indicated groups using oligo(dT)‐conjugated beads. Flowthroughs were collected, purified, and designated as non‐poly(A) RNAs. Poly(A) RNA enrichment was validated by detecting non‐poly(A) RNA (represented by 18S rRNA) to mRNA (represented by *GAPDH*) ratio using RT‐qPCR (Figure S4A, Supporting Information). Despite maintaining ac^4^C levels in total RNA and non‐poly(A) RNA (92.03% and 94.69% in *siPCBP1/2* and 116.58% and 99.04% in *siTDP43* compared to the si*NC* group, respectively), ac^4^C abundance was reduced to 69.54% and 85.45% of its level in the control group, respectively, in poly(A) RNA upon *PCBP1/2* and *TDP43* depletion (Figure 2C–E). However, *NAT10* knockdown resulted in decreased ac^4^C abundance in all categories of RNA: approximately 62.66% in total RNA, 69.67% in poly(A) RNA, and 82.57% in non‐poly(A) RNA of the ones in the si*NC* group. Meanwhile, the depletion of *PCBP1/2*, *TDP43*, and *NAT10* did not alter the abundance of other modifications in poly(A) RNAs, including N‐6‐methyladenosine (m^6^A), 5‐methylcytosine (m^5^C), and 5‐formylcytosine (f^5^C) (Figure 2F–H, respectively). These results indicate that PCBP1/2 and TDP43 specifically mediate poly(A) RNA ac^4^C formation in HEK293T cells.

### 2.3 Characterization of mRNA Acetylome in HEK293T Cells

**Figure 3:**
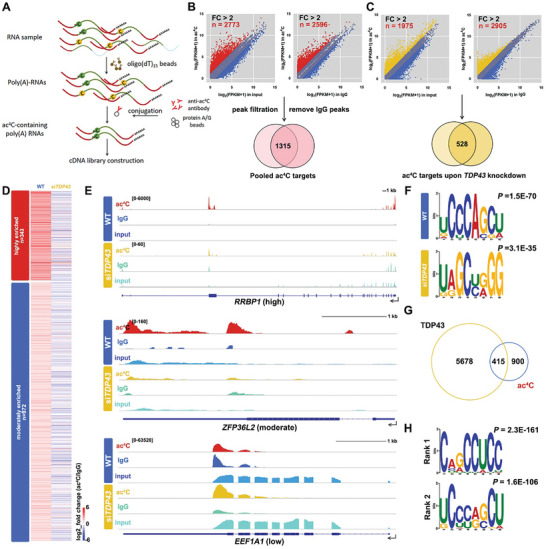
Transcriptome‐wide mapping of ac^4^C in HEK293T mRNAs. A) Schematic illustration of ac^4^C RNA‐immunoprecipitation tandem sequencing analysis (acRIP‐seq). Using oligo (dT)_15_ beads, poly(A) RNAs were enriched from total RNA extracted from HEK293T cells and subjected to affinity purification with anti‐ac^4^C antibody, cDNA pool construction, and further sequencing analysis. B‐C) ac^4^C(+) poly(A) RNAs were defined based on transcript enrichment levels in the ac^4^C‐enriched groups relative to the inputs and the IgG‐enriched groups in the WT (B) and the *siTDP43* (C) groups. Only transcripts that passed two‐tailed Student's *t*‐tests both in comparison to the inputs and the IgG groups were selected as pooled ac^4^C targets (*P <* 0.05). FC, fold change. D) Heatmap indicating enrichment levels of WT ac^4^C targets in WT and *siTDP43* mRNAs. The color key from red to blue indicated relative enrichment extents from high to low. E) IGV browser views of transcript reads in *RRBP1* (highly acetylated), *ZFP36L2* (moderately acetylated), and *EEF1A1* (not‐acetylated) transcripts mapped to the human reference genome (hg19). Reads in the acRIP, IgG‐enriched, and input groups are presented. The intron/exon (line/box) genomic structure is shown in dark blue. F) Enriched sequence motif analysis of ac^4^C peak clusters identified by acRIP‐seq. Up, ac^4^C motif in WT 293T mRNAs (*P* = 1.5E‐70). Down, ac^4^C motif in *siTDP43* mRNAs (*P* = 3.1E‐35). Binding motifs were analyzed by the MEME motif. G) Overlap between ac^4^C‐modified transcripts and TDP43‐bound mRNAs in 293T cells.^[^ ^18a^ ^]^ H) Enriched sequence motif of ac^4^C transcripts bound by TDP43. The top 2 sequences were presented.

To confirm the occurrence of ac^4^C within mRNAs and the presumed function of the NAT10/PCBP/TDP43 complex, RNA‐immunoprecipitation upon ac^4^C followed by sequencing (acRIP‐seq) was applied to map transcriptome‐wide ac^4^Cs in 293T poly(A) RNAs (**Figure** **3**A). Poly (A) RNAs isolated from the WT and *siTDP43* 293T cells were incubated with protein A beads conjugated to the anti‐ac^4^C antibody. The pulled‐down ac^4^C‐containing poly(A) RNA was subjected to cDNA library construction and SMART‐seq2 analysis. AcRIP‐seq reads were mapped to a reference human genome (hg19), and the peaks were identified upon enrichment relative to the input and immunoglobulin G (IgG) control groups (fold change > 2, *P* < 0.05, Figure 3B,C). Gene expression levels were assessed as fragments per kilobase of transcripts per million mapped reads (FPKM). Immunoprecipitation analysis was performed in duplicates or triplicates for the indicated groups (Figure S5A, Supporting Information). After filtration, 1315 genes were annotated as ac^4^C targets in WT 293T cells (referred as ac^4^C(+) as following). Based on the enrichment between the acRIP group and its corresponding IgG group, 343 genes were defined as highly acetylated genes (fold change (FPKM_ac4C_ + 1) / (FPKM_IgG_ + 1) > 5), 972 genes were annotated as moderately acetylated genes (fold change (FPKM_ac4C_ + 1) / (FPKM_IgG_ + 1) > 2 and meanwhile fold change (FPKM_ac4C_ + 1) / (FPKM_input_ + 1) > 2), while the rest were named not‐acetylated ones in the WT group (referred as ac^4^C(–), Figure 3D and Table S1, Supporting Information). Only 528 genes were designated ac^4^C(+) ones in the *siTDP43* group (Figure 3C and Table S2, Supporting Information). Representative IGV (Integrative Genomics Viewer) browser shots depicting genome and transcriptome alignments of the defined high and moderate ac^4^C(+) targets, as well as ac^4^C(–) genes (*RRBP1*, *ZFP36L2*, and *EEF1A1*, respectively), showed discrete peaks in ac^4^C(+) mRNAs that were ablated or attenuated by peak height in the IgG and/or input group (Figure 3E). Gene Ontology (GO) analyses revealed that ac^4^C(+) mRNAs functioned in various pathways, including RNA metabolism, chromatin remodeling, and signaling (Figure S5B, Supporting Information). Previous research identified cytidines at the wobble site of codons as ac^4^C‐preferred loci.^[^ ^6c^ ^]^ To investigate potential ac^4^C‐preferred sequences, the ac^4^C‐enriched peak data were subjected to MEME analysis,^[^ ^17^ ^]^ and a UCCCAGCU motif (*P* = 1.5E‐70) was found to be the most potent ac^4^C harboring sequences (Figure 3F). *TDP43* knockdown led to an altered motif named UAGCYRGG (Y = C/U, R = A/G, *P* = 3.1E‐35), indicating that mRNA acetylation was disrupted when TDP43 was absent. When integrating acRIP‐seq data with previously reported TDP43 and PCBP1 crosslinking and immunoprecipitation sequencing (CLIP‐seq) data, ≈30% of the putative ac^4^C‐harbored gene transcripts overlapped with the TDP43 and PCBP1 targets in 293T cells^[^ ^18^ ^]^ (Figure 3G; Figure S5C, Supporting Information). Intriguingly, the second enriched TDP43‐bound motif, UCYYRSCU (S = G/C, *P* = 1.6E‐106) presented high analogy to the ac^4^C harboring sequences (Figure 3H). Also, a group of ac^4^C(+) mRNAs harbored a TDP43‐preferred UG‐rich motif, UGUGCG (Figure S5D, Supporting Information). 282 of them were designated as high fidelity TDP43 ac^4^C targets, since they were reported as TDP43‐interacting transcripts, harboring UGUGCG motif, and presented decreased acetylation upon *TDP43* depletion (Figure S5E,F, Supporting Information). Among the high fidelity targets, 29.43% (83/282) localized in the ac^4^C(+) mRNAs containing the predicted UCCCAGCU motif (Figure S5G, Supporting Information). Collectively, these results demonstrate that TDP43 involves in ac^4^C production and the RNA binding capacity of PCBPs and TDP43 might account for their roles in mRNA acetylation.

### 2.4 Validation of the ac^4^C Abundance Variation upon *PCBP1/2* and *TDP43* Depletion

**Figure 4:**
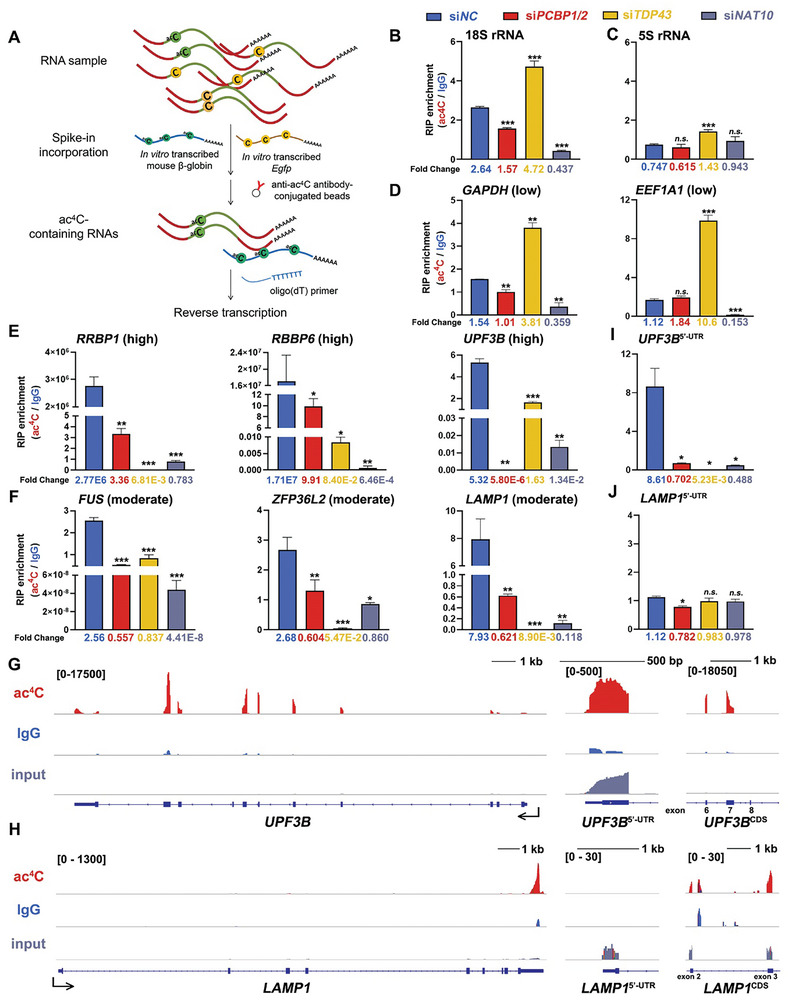
Depletion of PCBP1/2 and TDP43 resulted in decreased ac^4^C abundance or loss of acetylation in WT ac4C mRNAs. A) Schematic of acRIP‐qPCR analysis. RNA samples extracted from the control and *PCBP1/2*, *TDP43*, or *NAT10* knockdown groups were incorporated with the in vitro transcribed ac^4^C‐containing mouse β‐globin probe and ac^4^C‐null *Egfp* probe and enriched by anti‐ac^4^C antibody‐conjugated beads. The enriched RNAs by affinity purification were further reverse transcribed with oligo(dT)_30_ primer. B‐C) Validation of RT‐qPCR results of 18S and 5S rRNA recovered from acRIP. 18S rRNA served as a known ac^4^C target control. Mean ± SEM. ***P < 0.001. *n.s*., not significant. D‐F) RT‐qPCR results showing that low acetylated mRNAs (*GAPDH* and *EEF1A1*), highly acetylated mRNAs (*RRBP1*, *RBBP6*, and *UPF3B*), and moderately acetylated mRNAs (*FUS*, *ZFP36L2*, and *LAMP1*) varied in acRIP enrichment. Mean ± SEM. *P < 0.05. G‐H) IGV browser views of transcript reads of ac^4^C targets that either contained or did not contain 5′‐UTR acetylation peaks (*UPF3B* in G and *LAMP1* in H) in the ac^4^C‐, IgG‐enriched and the input in the control group. Enlarged views of the 5′‐UTR and selected exons within the CDS are presented. UTR, untranslated region. CDS, coding sequence. I‐J) RT‐qPCR results showing diverted enrichment levels of the 5′‐UTR of *UPF3B* (I) and *LAMP1* (J) in the indicated groups. Mean ± SEM.

ac^4^C abundance of selected targets, including transcripts with high, moderate, and low acetylation levels, was validated by RT‐qPCR. In general, in vitro transcribed and polyadenylated ac^4^C‐containing mouse β‐globin RNA and ac^4^C‐null *Egfp* mRNAs were incorporated into total RNA extracted from the control group and the indicated knockdown group. The mixture was further subjected to acRIP, and the enriched transcripts were reverse transcribed using oligo(dT) primers (**Figure** **4**A). RT‐qPCR results showed successful recovery of mouse β‐globin in all groups and failure in *Egfp* recovery (Figure S5H–I, Supporting Information). As an internal positive control, 18S rRNA harbored ac^4^C at two discrete sites in helices 34 and 45.^[^ ^6b^ ^]^ RT‐qPCR results showed that 18S rRNA was enriched in the control group while ac^4^C(–) 5S rRNA could not be enriched (Figure 4B,C). Although *PCBP1/2* knockdown led to a slight decrease and *TDP43* knockdown resulted in elevated 18S rRNA acetylation levels, the ac^4^C level of 18S rRNA sharply decreased upon *NAT10* depletion. While ac^4^C(–) transcripts (*GAPDH* and *EEF1A1*) were not enriched in the acRIP group in the control and *siPCBP1/2* groups, depletion of *TDP43* induced ac^4^C accumulation in these RNAs (Figure 4D). In contrast, transcripts with high and moderate ac^4^C(+) (*RRBP1*, *RBBP6*, and *UPF3B* as highly acetylated, *FUS*, *ZFP36L2*, and *LAMP1* as moderately acetylated) displayed ac^4^C level reduction upon deletion of both *PCBP1/2* and *TDP43*, either maintaining a lower ac^4^C level or loss of modification (Figure 4E,F). Meanwhile, the lack of *NAT10* decreased ac^4^C levels in all the above‐mentioned ac^4^C(+) transcripts.

Other than its distribution throughout the CDS of the gene body, previous research had reported an even stronger occurrence of ac^4^C at the 5′‐end in the proximity of the TSS.^[^ ^6^, ^7^ ^]^ IGV browser shots of the indicated ac^4^C(+) genes defined in the WT groups with either presence or absence of 5′‐UTR ac^4^C peaks (*UPF3B* and *LAMP1*, respectively, Figure 4G,H). RT‐qPCR results showed knockdown of *NAT10*, as well as the predicted adaptors *PCBP1/2* and *TDP43*, would lead to 5′‐UTR ac^4^C abundance reduction of 5′‐UTR ac^4^C(+) transcripts (Figure 4I). For ac^4^C(+) transcripts with no 5′‐UTR ac^4^C enrichment, neither depletion of *NAT10* nor adaptor knockdown altered their 5′‐UTR non‐acetylation states (Figure 4J). These results indicate that the NAT10/PCBP/TDP43 complex is responsible for mRNA acetylation within the 5′‐UTR and CDS, whereas ablation of each subunit leads to attenuation or loss of acetylation in ac^4^C(+) transcripts and aberrant gain of acetylation in ac^4^C(–) transcripts.

### 2.5 PCBP1/2 and TDP43 Facilitate NAT10 to Bind mRNAs

**Figure 5:**
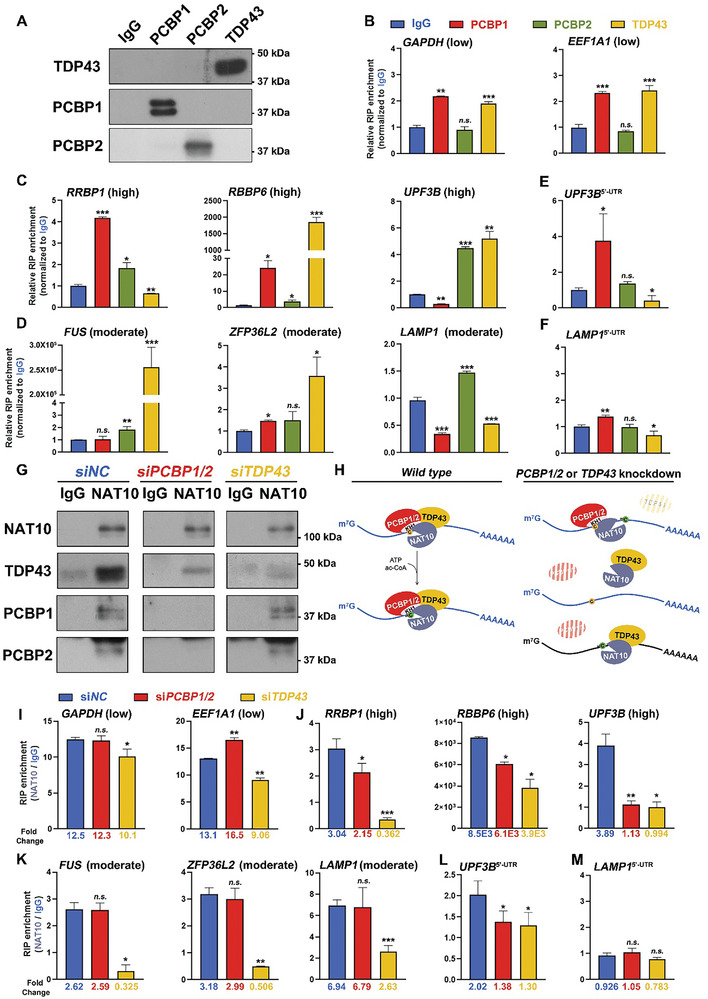
PCBP1/2 and TDP43 facilitated the binding of NAT10 to ac^4^C‐preferred mRNAs. A) Western blots of immunoprecipitated PCBP1, PCBP2, and TDP43 in endogenous RNA‐immunoprecipitation (RIP). Rabbit isotype IgG was applied as the control. B‐D) RT‐qPCR results showing that mRNAs that served as non‐preferred (*GAPDH* and *EEF1A1)*, highly preferred (*RRBP1*, *RBBP6*, and *UPF3B*), and moderately preferred (*FUS*, *ZFP36L2* and *LAMP1*) ac^4^C substrates were tethered by PCBP1/2 and TDP43 in varying degrees. Mean ± SEM. *P < 0.05, **P < 0.01, ***P < 0.001. *n.s*. not significant. E‐F) RT‐qPCR results showing that acetylation‐preferred mRNA 5′‐UTRs were diversely tethered by PCBP1/2 and TDP43. Mean ± SEM. G) Western blots of immunoprecipitated NAT10, TDP43, PCBP1, and PCBP2 in RIP by anti‐NAT10 antibody in the control (*siNC*) and the adaptor‐depleted groups (*siPCBP1/2* and *siTDP43*, respectively). The interaction between TDP43 and NAT10 was maintained when PCBP1/2 was depleted. Vice versa. H) Schematic illustration of NAT10/PCBP/TDP43 complex in mRNA acetylation. Under normal conditions, NAT10/PCBP1/TDP43 formed a stable heterogeneous tetramer, with PCBPs binding to NAT10 through their KH1 domain. By tethering mRNAs, PCBP1/2 and TDP43 recruited NAT10 to preferred mRNAs and NAT10 transferred acetyl groups to cytidines at the indicated sites. Loss of PCBP1/2 or TDP43 incurred instability in the complex, resulting in unspecific binding to non‐acetylation mRNA substrate or even failure in mRNA binding, consequently leading to aberrant ac^4^C formation. I‐K) RT‐qPCR results showing the varied connection between NAT10 and mRNAs of the indicated acetylation‐preferred groups upon *PCBP1/2* and *TDP43* knockdown. Mean ± SEM. L‐M) RT‐qPCR results showing the diverted connection between NAT10 and mRNA 5′‐UTRs of the *UPF3B* (L) and *LAMP1* (M) upon *PCBP1/2* and *TDP43* knockdown. Mean ± SEM.

In vitro biochemical and biophysical studies have shown that NAT10 contains an N‐terminal DUF domain, an ATPase‐cognate RNA helicase domain, an N‐acetyltransferase domain, and a C‐terminal tRNA‐binding domain.^[^ ^9^, ^13^ ^]^ Since PCBP1/2 and TDP43 contain diverse RNA‐binding domains (KH domains in PCBPs and RNA‐recognition motifs in TDP43) and a group of shared targets between ac^4^C(+) and PCBP/TDP43‐bound transcripts, we speculated that these subunits bridged NAT10 and potential mRNA substrates. To confirm their binding affinities toward mRNAs, PCBP1‐, PCBP2‐, and TDP43‐binding RNAs in 293T cells were immunoprecipitated using the indicated antibody‐conjugated protein A/G beads. The immunoprecipitation efficiency was validated by western blot analysis (**Figure** **5**A). The preferred ac^4^C substrates, defined as ac^4^C(+) transcripts in acRIP‐seq, were enriched in PCBP1‐, PCBP2‐, and TDP43‐bound groups individually or simultaneously when compared to the IgG group, whereas the affinity between adaptors and ac^4^C(–) transcripts was much lower (Figure 5B–D). Similarly, PCBP1/2 and TDP43 also showed an affinity for the 5′‐UTR ac^4^C(+) regions, whereas only a basal combination was detected in the ac^4^C(–) 5′‐UTRs (Figure 5E,F).

To further confirm whether these adaptors assist NAT10 in recruiting the preferred ac^4^C baits, endogenous NAT10‐RIP was performed on 293T cells upon *PCBP1/2* and *TDP43* knockdown. Western blot results showed successful enrichment of NAT10 in all groups, with maintained TDP43 co‐capture in the *siPCBP1/2* group and PCBP1/2 co‐capture maintained in the *siTDP43* group (Figure 5G). In WT 293T cells, PCBPs interact with NAT10 through their KH1 domains, and a putative model goes as the adaptor subunits in this complex tether the mRNA substrates, with NAT10 subsequently transferring the acetyl group to the cytidines (Figure 5H). In the absence of PCBP1/2 and TDP43, the acetyltransferase complex may become unstable or non‐specifically bind to mRNAs. Herein, the binding capability of NAT10 toward the ac^4^C(–) transcripts presented slight alterations, while such an interaction with the ac^4^C(+) transcripts was dramatically downregulated upon adaptor depletion individually or simultaneously (*RRBP1*, *RBBP6*, and *UPF3B* in the former group and *FUS*, *ZFP36L2*, and *LAMP1* in the latter group, Figure 5I–K). The 5′‐UTR acetylation showed a similar trend (Figure 5L,M). These results indicate that PCBP1/2 and TDP43, the adaptor subunits, but not the catalytic subunit NAT10, are capable of targeted mRNA binding, thus driving acetylation of the mRNA substrates.

### 2.6 PCBP1 is Responsible for mRNA ac^4^C Site Preference

**Figure 6:**
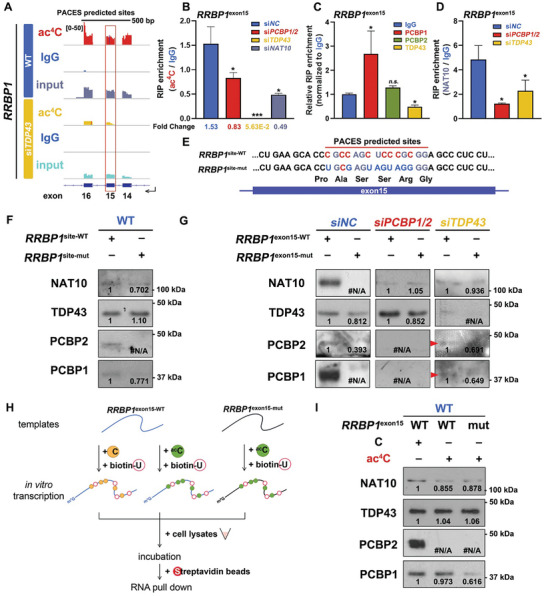
Affinity between PCBP1 and mRNA affected ac^4^C site preference. A) IGV browser shots of transcript reads of *RRBP1* exon 14 – 16 mapping in acRIP‐seq mapping to the human reference genome. Nucleotides 3299–3313 within *RRBP1* ^exon15^ (CGCCAGCUCCCGCGG) were predicted to harbor ac4C according to PACES, further designated as *RRBP1* ^site^. B) RT‐qPCR results confirming *RRBP1* ^exon15^ mRNA acetylation in the control group (*siNC*) but lost upon *PCBP1/2*, *TDP43*, or *NAT10* depletion. Mean ± SEM. *P < 0.05, ***P < 0.001. C) RT‐qPCR results showing PCBP1 affinity in interaction with *RRBP1* ^exon15^ mRNA in endogenous RIP. Mean ± SEM. *n.s*. not significant. D) RT‐qPCR results showing the affinity of NAT10 toward *RRBP1* ^exon15^ mRNA decreased or lost upon *TDP43* and *PCBP1/2* knockdown, respectively. Mean ± SEM. E) Illustration of RNA probe designed for RNA pull‐down. *RRBP1* ^site^ was cloned from HEK293T cDNA and in vitro transcribed with biotinylated‐uridine incorporation directly or after neutral mutation in which most cytidines within *RRBP1* ^site^ were substituted by other nucleosides (C3299U, C3302G, C3305U, C3307G, C3308U, C3309A, and C3311G, indicated as *RRBP1* ^site1–WT^ and *RRBP1* ^site1–mut^, respectively). F) RNA pull‐down results indicated PCBP1/2 presented specific affinity toward WT *RRBP1* ^site^. G) RNA pull‐down results indicated diverted affinity between *RRBP1* ^exon15^ and NAT10/PCBP/TDP43 complex subunits in the control group and upon *PCBP1/2*, *TDP43*, and *NAT10* knockdown. H) Schematic of RNA pull‐down analysis with cytidine and acetylated cytidine incorporated probes. WT and mutated *RRBP1* exon 15 were in vitro transcribed with cytidines and acetylated cytidines as indicated. Biotinylated uridines were also incorporated for further enrichment. The RNA probes were mixed with the indicated cell lysates and subjected to affinity pull‐down. I) RNA pull‐down results showing the affinity between the PCBP/TDP43/NAT10 complex and the indicated RNA probes. PCBP1 specifically interacted with *RRBP1* ^exon15^, discarding the incorporation of ac^4^C, while PCBP2 merely contacted with ac^4^C(–) RNAs. TDP43 presented no difference in binding with ac^4^C‐containing or ac^4^C‐lacking mRNAs. The gray values of the bands were quantified by ImageJ and normalized to the control group pulled down through probes containing WT sequences. #N/A indicated that no band detected.

PCBP1/2 is known to have high affinity and specificity for cytidine‐rich polypyrimidine sequences,^[^ ^19^ ^]^ whereas TDP43 has been reported to bind UG‐rich motifs within long clusters.^[^ ^18^, ^20^ ^]^ To investigate whether PCBP1/2 affected mRNA acetylation site preference, we used PACES, a software for ac^4^C site prediction, to uncover the association between adaptors and ac^4^C(+) transcripts.^[^ ^21^ ^]^ An ac^4^C(+) target of the highly acetylated group, *RRBP1*, was predicted to harbor ac^4^Cs in exon 15 (nucleotide 3299 – 3313, **Figure** **6**A). The RT‐qPCR results revealed *RRBP1* exon 15 harbored ac^4^C in the si*NC* group, whereas its acetylation abundance sharply decreased upon *PCBP1/2* and *TDP43* knockdown (Figure 6B). RIP followed by RT‐qPCR revealed that PCBP1 had a high preference to *RRBP1* exon 15, while the interaction between NAT10 and such RNA sequences was ablated or attenuated upon *PCBP1/2* and *TDP43* depletion, respectively (Figure 6C,D). The predicted ac^4^C harboring sites, together with the upstream and downstream 10 base pairs (referred as *RRBP1* ^site–WT^), were subcloned and subjected to site mutagenesis, in which all cytidines were substituted with other nucleosides (referred as *RRBP1* ^site–mut^) without altering the encoding peptides (Figure 6E). The *RRBP1* ^site–WT^ and *RRBP1* ^site–mut^ were thus in vitro transcribed with biotinylated uridine incorporation. Biotin‐avidin interaction‐based RNA pull‐down assays showed that TDP43 had no preference between these two RNA probes, whereas PCBP1/2 and NAT10 only bound to the WT sequence (Figure 6F). Furthermore, full‐length *RRBP1* exon15 was subcloned, mutated, and transcribed into a cytidine‐reserved form (*RRBP1* ^exon15–WT^) and a site‐specific cytidine mutant (*RRBP1* ^exon15–mut^). Incorporating the probes did not change *RRBP1* expression levels in the inputs (Figure S6A, Supporting Information). The pull‐down efficiency of the referred RNAs was validated using RT‐qPCR (Figure S6B, Supporting Information). While PCBP1/2 and NAT10 interacted only with WT *RRBP1* exon15, TDP43 maintained the interaction with its mutant, although with a slightly decrease in affinity (Figure 6G). Although *TDP43* depletion did not obstacle the NAT10‐PCBP1/2‐*RRBP1* exon 15 interaction, the connection between NAT10 and *RRBP1* exon 15 was abolished upon *PCBP1/2* knockdown. Meanwhile, the preference of TDP43 to WT *RRBP1* exon 15 over its mutant was attenuated. Furthermore, acetylated cytidines were incorporated in WT and mutated *RRBP1* exon15 RNAs and the probes were subjected to RNA pull‐down analysis (Figure 6H). Substitution of cytidines by ac^4^C did not alter the binding affinity between NAT10, TDP43, PCBP1, and WT *RRBP1* ^exon15^, while ectopic ac^4^C incorporation within *RRBP1^exon15^* mutant lost its interaction with PCBP1 (Figure 6I; Figure S6C, Supporting Information). Meanwhile, the ac^4^C‐containing probe lost its interaction with PCBP2. These results indicate that PCBP1/2 and TDP43 function as RNA adaptors, rather than readers, to assist NAT10 in anchoring site‐specific RNA sequences and facilitating cytidine acetylation.

### 2.7 PCBP1/2‐TDP43/NAT10 Functions as an mRNA Acetyltransferase Complex in Mouse Testes

**Figure 7:**
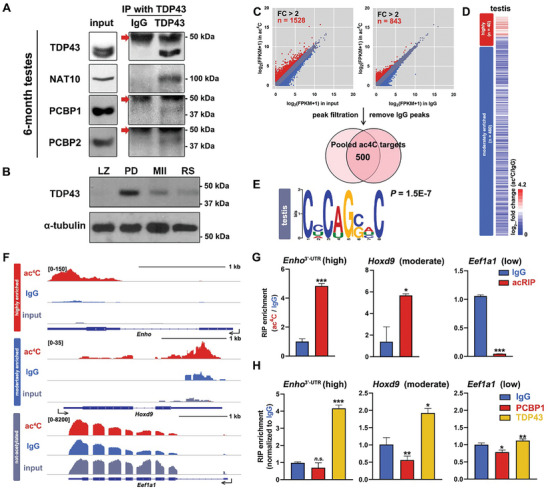
NAT10/PCBP/TDP43 complex functioned in mRNA acetylation in mouse testes. A) Endo‐IP results showing the spontaneous coordination of TDP43, NAT10, and PCBP1/2 in mouse testes. Anti‐TDP43 antibody was applied in endo‐IP. Arrows indicated the beads band. B) Western blot results showing TDP43 expression in spermatocytes isolated from WT mouse testes using flow cytometry sorting (FACS) (LZ, leptotene and zygotene, PD, pachytene and diplotene, MII, metaphase II and RS, round spermatids). α‐tubulin was blotted as a loading control. C) Acetylated mRNAs in mouse testes were defined through enrichment abundance between the acRIP and the inputs and IgG‐enriched groups. FC, fold change. D) Heatmap indicating enrichment levels of WT mouse testes ac^4^C targets. The color key from red to blue indicates relative enrichment extents from high to low. E) Enriched sequence motif analysis of ac4C peak clusters identified by acRIP‐seq in mouse testis. A CCHCAGSHC (H = C/U/A, S = C/G, P = 1.5E‐7) motif was detected by MEME analysis. F) IGV browser views of highly acetylated (*Enho* 3′‐UTR), moderately acetylated (*Hoxd9*), and not‐acetylated (*Eef1a1*) transcripts mapping to the mouse reference genome (mm10). Reads in the acRIP, IgG, and input groups are presented. The intron/exon (line/box) genomic structure is shown in dark blue. G) RT‐qPCR results showing diverted acetylation abundance of highly acetylated (3′‐UTR of *Enho*), moderately acetylated (*Hoxd9*), and low acetylated mRNAs (*Eef1a1*). Mean ± SEM. *P < 0.05, ***P < 0.001. H) RT‐qPCR results showing TDP43 affinity toward preferred ac^4^C‐targeted mRNAs in mouse testes. Mean ± SEM. **P < 0.01.

Previous research has found abundant mRNA ac^4^Cs in male spermatogenesis and germ cell‐specific ablation of *Nat10* result in meiotic entry defects, including aberrant homologous chromosome synapsis, DNA double‐strand break (DSBs) repair failure, and pachytene stage‐arrested spermatocytes in mouse testes.^[^ ^11^ ^]^ Moreover, the loss of TDP43 in male mouse germ cells results in mid‐pachytene‐stage arrested spermatocytes and severe infertility.^[^ ^22^ ^]^ Similarly, mouse embryos loss of maternal NAT10 and TDP43 lacked developmental competence, with most of them arrested at the 2‐cell stage.^[^ ^12^, ^23^ ^]^ The vital role of the NAT10/PCBP/TDP43 complex in ac^4^C modification regulation, as well as its similar function in mouse spermatogenesis and early embryogenesis, prompted us to examine its existence and potential functions across species. Western blot results showed that NAT10 and PCBP1/2 spontaneously interacted with TDP43 in mouse testes by endo‐IP using the anti‐TDP43 antibody (**Figure** **7**A). Western blotting results showed a fairly weak expression of TDP43 in spermatocytes at the leptotene and zygotene stages, but a higher level in pachytene and diplotene stage spermatocytes, where TDP43 and NAT10 might function together in ac^4^C production (Figure 7B). mRNAs extracted and purified from mouse testes were subjected to acRIP‐seq, and ac^4^C(+) transcripts were defined by comparing their enrichment levels between the acRIP group and the IgG‐enriched and input groups (Figure 7C). The analysis was performed in triplicates, and a high correlation was detected within the groups (Figure S7A, Supporting Information). Among these, transcripts from 500 genes were defined as ac^4^C(+) mRNAs, of which 40 were categorized into the high acetylation group and 460 into the moderate acetylation group (Figure 7D; Table S3, Supporting Information). GO analysis showed that the ac^4^C(+) genes in the mouse testes mainly function in developmental processes and transcriptional regulation (Figure S7B, Supporting Information). A similar motif, CHCAGSHC (H = C/U/A, S = C/G, *P* = 1.5E‐7) was designated as the ac^4^C‐preferred sequence (Figure 7E). Representative IGV browser shots displayed ac^4^C(+) targets (3′‐UTR of *Enho* from the high acetylation group and *Hoxd9* from the moderate acetylation group) and ac^4^C(–) transcripts (*Eef1a1*) recovered from acRIP‐seq, presenting genome and transcriptome alignment mapping to the mouse genome (mm10, Figure 7F). acRIP followed by RT‐qPCR results confirmed the diverse acetylation levels of ac^4^C(+) and ac^4^C(–) mRNAs in both the CDS and UTRs (Figure 7G; Figure S7D–F, Supporting Information). RIP followed by RT‐qPCR results verified binding affinity between TDP43 and ac4C‐preferred transcripts in mouse testis, with a decreased affinity from highly acetylated ones (*Enho* 3′‐UTR) and moderately acetylated ones (*Hoxd9*, *Rnf183*, and *Hoxb7*) to ac4C(–) mRNAs (*Eef1a1* and *Gapdh*), discarding the location of the peak (Figure 7H; Figure S7H–J, Supporting Information). Acetylation of 18S rRNA and its interaction with adaptors were validated as controls (Figure S7C,G, Supporting Information). These data suggest that the ac^4^C acetyltransferase NAT10/PCBP/TDP43, which exists in multiple cell types and across species, can direct mouse testicular mRNA acetylation and is essential for spermatogenesis.

## 3 Discussion

The discovery of RNA modifications has given rise to emerging studies on the epigenetics – epitranscriptome. These modified bases are responsible for several aspects of RNA metabolism. RNAs in all categories, including rRNAs, tRNAs, mRNAs, long non‐coding RNAs (lncRNAs),^[^ ^24^ ^]^ microRNAs (miRNAs),^[^ ^25^ ^]^ circular RNAs (cirRNAs),^[^ ^26^ ^]^ and retrotransposons^[^ ^27^ ^]^ are chemically modified bases. The functions of various modifications in mRNA metabolism have recently been discussed. One of the most abundant modifications, m^6^A, exists in the CDS and presents a preference for the 3′‐UTR, especially in the vicinity of the stop codon.^[^ ^28^ ^]^ Such modifications earn diverse roles in mRNA metabolism, as YTH‐domain factor 2 (YTHDF2), an m^6^A reader that interacts with the substrate mRNA and recruits it to cytoplasmic P‐bodies for degradation, while other readers,^[^ ^4^, ^29^ ^]^ YTHDF1/3 promotes its translation.^[^ ^3b^ ^]^ The m^5^C modification has a different localization pattern, as a strong preference is presented in regions downstream and close to the TSS.^[^ ^2^, ^30^ ^]^ With the help of the Aly/REF export factor (ALYREF), m^5^C instructs the nuclear/cytosolic translocation of substrate mRNAs. Expression of the modified base‐transferase complex, namely the writers, guards timely and appropriate mRNA modifications. Loss of either methyltransferase‐like 3 (METTL3) or METTL14, the two core subunits of the N‐6‐methyltransferase complex, leads to m6A reduction in poly(A) RNAs.^[^ ^4^, ^28^ ^]^ Similarly, depletion of the m^5^C writer NSUN2 resulted in decreased m^5^C abundance and inhibited mRNA transportation from the nucleus to the cytosol.^[^ ^2^ ^]^ These advances call for in‐depth research on other modifications, including ac^4^C, regarding the constituents and structure of the writer complex, factors affecting sequence selectivity and specificity, and functions of mRNA modifications.

In this study, we clarified the constituents of the writer complex involved in the to‐date only acetylation modification, ac^4^C, in the mammalian mRNAs. In addition to the core catalytic subunit NAT10, three mRNA‐binding proteins, PCBP1/2 and TDP43, were found to serve as adaptors for tethering NAT10 and substrate mRNAs (Figure 1). The NAT10/PCBP/TDP43 complex presented high specificity for acetylation of mRNAs, which did not affect acetylation of non‐poly(A) RNAs or other modifications in mRNAs (Figures 2 and 4). The adaptors were also responsible for substrate selection in acetylation, as 1) the adaptors facilitated the interaction of NAT10 with ac^4^C‐preferred mRNAs and did not affect its affinity to mRNAs with a low acetylation tendency (Figures 3 and 5); 2) the capability of PCBPs to tether cytidine‐rich sequences assisted the complex in anchoring ac^4^C‐preferred motifs since a UCCCAGCU sequence was detected as an ac^4^C(+) motif in HEK293T mRNAs (Figure 3); 3) PCBP1 could direct the binding and further acetylation to some specific sites in mRNAs, including *RRBP1* exon 15 (Figure 6). The interaction of NAT10/PCBP/TDP43 was also conserved among cell types and across species, as the subunits were co‐expressed in oocytes and spermatocytes, and maintained their interactions in the mouse testes (Figure 7; Figure S2, Supporting Information).

Writer complexes toward RNA modification consist of multiple subunits. For instance, eigh subunits are reported to function in the N‐6‐methyltransferase complex, including METTL3 and METTL14 as the core catalytic subunits,^[^ ^28^, ^31^ ^]^ Cbl proto‐oncogene like 1 (CBLL1, also known as HAKAI) to assist in complex conjugation,^[^ ^32^ ^]^ Zinc finger CCCH‐type containing 13 (ZC3H13) as the prerequisite for the writer complex localizing in the nucleus,^[^ ^33^ ^]^ and Vir like m6A methyltransferase associated (VIRMA, also known as KIAA1429), Wilm's tumor 1‐associated protein (WTAP)^[^ ^34^ ^]^ and RNA binding motif protein 15 and 15B (RBM15/15B)^[^ ^24b^ ^]^ as adaptors in RNA linking. When lacking WTAP, reduced RNA‐binding capability of METTL3 and decreased mRNA m^6^A abundance were detected in HeLa cells.^[^ ^34b^ ^]^ Attenuated m^6^A levels have also been detected in *XIST* mRNAs when RBM15/15B were depleted, further impairing *XIST*‐directed gene silencing.^[^ ^24b^ ^]^ Here, we report the NAT10/PCBP/TDP43 complex mediates acetylation in mammalian mRNAs. The complex works with multiple subunits, which is stable in RNA binding for selected substrates, and might also be structured, since subunits in a multiple‐component complex might mutually promote stability.^[^ ^35^ ^]^ Adaptors divert the RNA‐binding protein families in the complex, enhancing the RNA binding efficiency. Moreover, PCBP1/2 homologs have similar functions in mRNA acetylation, in addition to similar structures. It is possible that being an adaptor of NAT10 might be a part of the functions of PCBP1/2 and TDP43. Previous research has found that PCBP1 functions as an 8‐oxo‐7,8‐dihydroguanosine (8‐oxo‐G) reader to induce apoptosis.^[^ ^36^ ^]^ TDP43 binds to UG‐rich mRNAs and regulates their stability and polyadenylation.^[^ ^20^, ^37^ ^]^ In addition, there may be other RBPs working in the N‐4‐acetyltransferase complex, either as adaptors or regulatory subunits, since research on the structures of NAT10 and the entire complex is still limited. Further research on how these proteins assemble and how the multi‐subunit complex functions in integrity is required.

Recently, the physiological roles of NAT10 have been discussed. Depletion or ablation of NAT10 did not affect cell viability but hampered cell proliferation and migration.^[^ ^6^, ^10^ ^]^ Germ cell‐specific knockout of NAT10 resulted in severe sterility in mice of both genders, as *Nat10*‐null oocytes harvested from *Nat10^fl/fl^;Zp3*‐Cre female mice were poor in quality and unable to experience CCR4‐NOT complex‐guarding maternal mRNA clearance during meiotic maturation, whereas *Stra8*‐Cre‐driven NAT10 removal from gametocytes ahead of meiosis resulted in a developmental arrest at the pachytene stage.^[^ ^11^, ^12^ ^]^ Similarly, the spermatocyte‐specific knockout of *Tdp43* led to subfertility and pachytene stage arrest in mice.^[^ ^22^ ^]^ Shared DSB repair defects and non‐homologous chromosome synapses in *Nat10*‐ and *Tdp43*‐null spermatocytes showed that NAT10 and TDP43 could function together in vivo. Further research on whether and how the NAT10/PCBP/TDP43 complex functions together is required.

In summary, we identified that NAT10/PCBP/TDP43 collaborates and mediates mRNA acetylation. While NAT10 serves as the core catalytic subunit, PCBP1/2 and TDP43 assist in selecting substrates, binding to mRNA, and stabilizing the complex. Loss of the constituents of the complex resulted in attenuated or ablated acetylation levels in ac^4^C(+) mRNAs, with missed cytidine‐rich ac^4^C motifs upon *TDP43* knockdown. Although the catalytic preference of NAT10 toward substrates remains unknown, PCBP1 and other potential adaptors could affect the ac^4^C site by recruiting NAT10 to their anchoring sequences.

## 4 Experimental Section

### Cell Culture

Human embryonic kidney 293T (HEK293T) cells were cultured in Dulbecco's Modified Eagle Medium (DMEM, GIBCO) plus 10% fetal bovine serum (FBS, GIBCO) and 1% penicillin‐streptomycin solution (Hyclone) in a humidified incubator, at 37 °C in 5% (v/v) CO_2_ atmosphere.

### Animals

Wild‐type (WT) ICR mice were obtained from the Zhejiang Academy of Medical Science, China. Mice were maintained under specific pathogen‐free (SPF) conditions in a controlled environment at 20–22 °C, within a 12/12 h light/dark cycle, 50–70% humidity, and food and water provided ad libitum. All animal experiments were conducted in accordance with the guidelines and regulations of Zhejiang University. The experimental protocol (ZJU20210252) was approved by the Institutional Animal Care and Research Committee of Zhejiang University.

### Oocyte Collection

The 28‐day‐old female mice were intraperitoneally injected with 5 IU of pregnant mare serum gonadotropin (PMSG, Ningbo Sansheng Pharmaceutical Co., Ltd., P. R. China) and enthanized 48 h later. Oocytes were harvested in M2 medium (M7167, Sigma‐Aldrich).

### Isolation of Spermatocytes

6‐week‐old male mice were applied for collecting spermatogenic cells at different stages. The fluorescence‐activated cell sorting (FACS) was performed following a published protocol.^[^ ^38^ ^]^ The testes were separated from the tunica albuginea and incubated with 5 mL PBS with 120 U mL^−1^ collagenase type I (Thermo Fisher Scientific, 17 100 017) for 10 min at 32 °C with gentle agitation. The dispersed seminiferous tubules were subjected to digestion with a buffer containing 0.25% Trypsin (Gibco, 25 200 072) and 0.1 mg mL^−1^ DNase I (Sigma‐Aldrich, DN25), which was then terminated with the addition of 1/10 volume of FBS. The gathered suspension was filtered through a 70 µm filter and centrifuged to collect cells. The cells were resuspended at a final concentration of 10^6^ cells per mL in Dulbecco's modified Eagle's medium (DMEM, C11995500BT, Gibco) with Hoechst 33 342 (5 µg per 10^6^ cells, Thermo Fisher Scientific) and 5 µL DNase I, gentle rotated for 30 min at 34 °C, and stained with propidium iodide (PI, 1 µg per 10^6^ cells, 25535‐16‐4, Sigma‐Aldrich) at 25 °C. Cell populations were then collected according to their fluorescent label with Hoechst 33 342/PI staining using FACS (BD Bioscience).

### Plasmid Transfection and RNA Interference

Human *NAT10, THUMPD1, PCBP1, PCBP2*, and *TDP43* were amplified by PCR from HEK293T cDNA pools and ligated into pDEST‐based eukaryotic expression vectors. Transient plasmid transfection of HEK293T cells was performed using Lipofectamine 2000 (Invitrogen, Carlsbad, CA, USA). Transient siRNAs (si*NC*, si*PCBP1*, si*PCBP2*, and si*TDP43*) were transfected into HEK293T cells using Lipofectamine RNAiMAX (Invitrogen). After 48 h of transfection in 24‐well plates or 96 h of transfection twice in 10‐cm dishes, HEK293T cells were collected and lysed for RNA isolation and western blotting analysis. The corresponding siRNA sequences used for gene knockdown are listed in Table S4, (Supporting Information).

### Affinity Purification and Mass Spectrometry Analysis

HEK293T cells were transfected with pDEST‐*Flag* and pDEST‐*Flag*‐*NAT10* for 24 h. Cell lysates were subjected to anti‐FLAG affinity gel (A4596; Sigma‐Aldrich, St. Louis, MI, USA) enrichment. For affinity purification in mouse testis, lysates gathered as described in Immunoprecipitation were incubated and purified by the indicated antibody‐conjugated beads. Bead‐bound proteins were subjected to sodium dodecyl sulfate (SDS)‐polyacrylamide gel electrophoresis (PAGE), Coomassie blue staining, and proteome analysis by mass spectrometry (QR HF‐X; Thermo Fisher Scientific, Waltham, MA, USA). Obitrap mass spectrometry data were processed using Maxquant version 1.6.10.43 (Max Planck Institute for Biochemistry, Martinsried, Germany) for feature detection, database searching, and protein quantification using the Swiss‐Prot human reference proteome. Oxidation and carbamidomethylation were set as the fixed modifications. Protein identification was performed using 10 ppm peptide tolerance.

### Immunoprecipitation

HEK293T cells transfected with the indicated plasmids for 48 h or WT 293T cells were harvested in lysis buffer (50 mM Tris‐HCl [pH 7.5], 150 mM NaCl, 1 mM EDTA, 1% Triton X‐100, and 1 mM PMSF). After centrifugation, lysates were subjected to immunoprecipitation (IP) using anti‐FLAG affinity gels. After incubation for 2 h at 4 °C, beads were gathered and rinsed with lysis buffer. For endogenous immunoprecipitation (endo‐IP) analysis, 293T cells and mouse testes were lysed and homogenized in lysis buffer. The indicated antibodies were mixed and conjugated with Protein A Sepharose 4 Fast Flow beads (GE Healthcare, Chicago, IL, USA) for 1 h at 25 °C. Rabbit (DA1E) mAb IgG XP Isotype (3423; Cell Signaling Technology, Danvers, MA, USA) was used as the control. After centrifugation, the lysates and antibody‐conjugated beads were incubated for 4 h at 4 °C. Bead‐bound proteins were eluted with SDS sample buffer for western blot analysis.

### Western Blot Analysis

Cell lysed with SDS sample buffer or eluted proteins in Ips were heated for 10 min at 95 °C. Lysates were separated through SDS‐PAGE, electrophoretically transferred to PVDF membranes (Millipore Crop., Bedford, MA, USA), and blocked with TBST containing 5% (m/v) non‐fat milk (Sangon Biotech, Shanghai, China) for 30 min at 25 °C. After probing with primary antibodies, the membranes were washed three times with TBST containing 20 mM Tris‐HCl (pH 7.5), 150 mM NaCl, and 0.1% (v/v) Tween‐20, and incubated with the corresponding horseradish peroxidase (HRP)‐linked secondary antibodies for 1 h at RT. After three washes with TBST, the bound antibodies were detected using WESTAR SUPERNOVA (Cyanagen, Bologna, Italy) according to the manufacturer's instructions. The antibodies and their dilutions are listed in Table S7, (Supporting Information).

### Immunofluorescence Analyses

Mounted 293T cells on glass and collected oocytes were fixed in 4% (m/v) paraformaldehyde (PFA) for 30 min at 25 °C. After permeabilization in PBS with 0.5% Triton X‐100 for 25 min at 25 °C, the samples were blocked in PBS with 1% bovin serum albumin (BSA, Sangon Biotech) and incubated with the primary antibodies at the indicated dilution overnight at 4 °C (see in Table S7, Supporting Information). For mouse testes, the testes were fixed in 4% PFA, embedded in paraffin, and sectioned. The slides were deparaffinized, rehydrated, and incubated with 10 mM sodium cirtrate solution (pH 6.0) for 15 min at 95 °C. After cooling down to 25 °C, the sections were washed, blocked with 10% goat serum (ZLI‐9065, ZSGB‐Bio, Beijing, China), and incubated with primary antibodies. The samples were washed and incubated with Alexa Fluor 488‐ or 594‐conjugated secondary antibodies (Jackson Immuno Research Laboratories), and DAPI (Molecular Probes) for 30 min at 25 °C. Mounted 293T cells and oocytes were imaged using a Zeiss LSM710 confocal microscope (Germany). Images of the testis slides were acquired using an epifluorescence microscope (Nikon Eclipse 80i, Japan).

### Isolation of Total, Non‐Poly(A), and Polyadenylated RNAs

Total RNAs were extracted from the indicated samples using the Invitrogen Ambion TRIzol reagent (Thermo Fisher Scientific) according to the manufacturer's instructions. DNA was removed by treatment with Turbo Dnase (Thermo Fisher Scientific). Polyadenylated RNAs (poly(A) RNAs) were enriched using oligo (dT)_25_ Dynabeads (Thermo Fisher Scientific) for HPLC‐MS/MS, dot blot analysis, and acRIP‐seq, according to the manufacturer's instructions. The flowthroughs were collected as non‐poly(A) RNAs, including 18S and 28S rRNAs, and precipitated using isopropanol. Purity was evaluated by RT‐qPCR using primers specific for 18S rRNA and *GAPDH* (see in Table S6, Supporting Information for primer descriptions) as described in the following section (Reverse transcription and quantitative polymerase chain reaction).

### Reverse Transcription and Quantitative Polymerase Chain Reaction

RNAs isolated and purified by oligo(dT) beads, extracted from the indicated RNA interference groups, and mRNAs harvested by acRIP were reverse‐transcribed, and cDNA abundance was evaluated using quantitative polymerase chain reaction (qPCR). For poly(A) RNA enrichment qualification, RNAs were reverse‐transcribed with random primers using PrimeScript II Reverse Transcriptase (Takara Bio, Shiga, Japan) according to the manufacturer's instructions. To evaluate RNA interference efficiency, total RNAs extracted from the indicated groups were reverse transcribed with oligo (dT)_30_ primers (see the corresponding sequences in Table S6, Supporting Information) using M‐MLV Reverse Transcriptase (28 025; Invitrogen) following the manufacturer's instructions. To evaluate mRNA acetylation abundance, immunoprecipitated RNAs were reverse transcribed with oligo (dT)_15_ primers using PrimeScript II Reverse Transcriptase. The cDNAs were subjected to qPCR using Power SYBR Green PCR Master Mix (Applied Biosystems, Life Technologies) and a Bio‐Rad CFX96 Touch Real‐Time PCR system. For each experiment, qPCR was performed in triplicates.

### Ac^4^C Detection and Quantification by HPLC‐MS/MS

HPLC‐MS/MS analysis of ac^4^C was performed as previously described.^[^ ^11^, ^39^ ^]^ In brief, RNAs were digested with Nuclease P1 (Sigma‐Aldrich, N8630) at 1 U/400 ng RNA in reaction buffer (100 mM ammonium acetate, pH 5.5, 2.5 mM NaCl and 250 µM ZnCl_2_) for 2 h at 37 °C. 5′‐ and 3′‐end phosphate groups were removed by Antaretic Phosphatase (NEB, M0289S) at 1 U/100 ng RNA in Antaretic Phosphatase buffer (NEB, B0289S) for 2 h at 37 °C. Samples were further lyophilized, reconstituted in 50 µL 20% (v/v) acetonitrile, and injected into the LC‐MS/MS (SCIEX, QTRAP 6500+ LC‐MS/MS, USA).

### In Vitro Transcription of ac^4^C‐Containing β‐Globin and Designed Biotinylated Oligoribonucleotides

The mouse β‐globin DNA template (corresponding sequences in Table S5, Supporting Information) was in vitro transcribed using a High Yield T7 CAP 1 AG+ac^4^CTP mRNA Synthesis Kit (Jena Bioscience) according to the manufacturer's instructions. In brief, 200 ng DNA templates were subjected to in vitro transcription for 4 h at 37 °C. After treatment with Turbo Dnase, poly(A) tails (for around 200 to 250 base pairs) were added to the β‐globin RNAs using the Poly(A) Tailing Kit (Invitrogen, AM1350). The polyadenylated RNAs were further recovered from the phenol: chloroform = 1:1 (v/v) treatment and isopropanol precipitation and diluted with Rnase‐free H_2_O.

The designed biotinylated oligonucleotides of the indicated mRNAs were cloned by PCR from the 293T cDNA pool and were in vitro transcribed using the mMESSAGE mMACHINE SP6 kit (AM1340; Invitrogen) following a modified protocol according to the manufacturer's instructions. Biotinylated UTP (Bio‐16‐UTP; AM8452; Invitrogen) was simultaneously added to the reaction mixture.

### Acetylated RNA Immunoprecipitation

To map global ac^4^C in poly(A) RNAs and evaluate ac^4^C abundance in individual mRNAs, acetylated RNA immunoprecipitation (acRIP) was performed using the anti‐ac^4^C antibody as previously described with minor modifications.^[^ ^40^ ^]^ Either anti‐ac^4^C antibody (1 µg) or rabbit monoclonal IgG isotype control (1 µg) was conjugated to 300 µg Dynabeads Protein A (ThermoFisher Scientific, 10002D) in DPBS for 1 h at RT. Beads were rinsed twice and resuspended with 100 µL acRIP buffer containing DPBS, 0.05% Triton X‐100, and 0.1% BSA. For acRIP‐seq, poly(A) RNAs enriched from the indicated samples were incorporated into in vitro transcribed β‐globin probes containing ac^4^C (1:1000) and subjected to affinity purification. For acRIP‐seq sample preparation, the RNAs were eluted into 100 µL acRIP buffer containing 80 U Recombinant Ribonuclease Inhibitor (RRI; Takara) and mixed with antibody‐conjugated beads. After incubation for 4 h at 4 °C, beads were rinsed three times with pre‐cold acRIP buffer. The immunoprecipitated RNAs were eluted by Proteinase K (PK, 50 µg, Sigma Aldrich) digestion in 100 µL PK buffer containing 100 mM Tris‐HCl, pH 7.5, 150 mM NaCl, 12.5 mM NaCl, 12.5 mM EDTA, 2% (m/v) SDS and 40 U RRI, for 1 h at 37 °C. The elutions were extracted by Phenol:Chloroform (1:1 v/v) and ethanol precipitation using 0.3 M acetate sodium, pH 5.5, and 2.5 volumes of 100% ethanol overnight at ‐20 °C. The precipitate was resuspended in Rnase‐free H_2_O for further analysis. For acRIP‐qPCR, DNase‐treated total RNAs were mixed with ac^4^C‐containing β‐globin probes (1:1000). ac^4^C‐enriched and IgG‐enriched RNAs in each sample were reverse transcribed with oligo (dT)_30_ primers using PrimeScript II Reverse Transcriptase (2690; Takara) and subjected to qPCR analysis. The efficiency of acRIPs was evaluated by the enrichment of mouse‐globin. The primers used are listed in Table S6, (Supporting Information).

For acRIP‐seq, acetylated mRNAs were subjected to RNA‐seq using the Smart‐seq2 method with minor modifications. In brief, mRNAs from input samples were diluted to 0.2 ng µL^−1^, and 2.5 µL were used for library construction. For IP samples of the IgG and acRIP groups, as the concentration of mRNAs is too low to be quantified, 2.5 µL was used to construct the library. The samples were incubated with oligo(dT) primers and a deoxynucleoside triphosphate mixture for 3 min at 72 °C, and cDNA pools were obtained by Smart‐seq2 reverse transcription reactions. After the first‐strand reaction, the cDNA was pre‐amplified with limited cycles (≈12 cycles). Sequencing libraries were constructed with 0.5 ng cDNA using the TruePrep DNA Library Prep Kit V2 for Illumina (TD503; Vazyme, Nanjing, China), according to the manufacturer's instructions. Barcoded libraries were pooled and sequenced on the Illumina NovaSeq 6000 platform in the 151 bp paired‐end mode.

### acRIP‐Seq Analysis

Raw reads were trimmed to remove low‐quality bases and adaptor sequences using Trim Galore v0.6.7. Reads were further mapped to the human (hg19) and mouse (mm10) genomes using STAR v2.7.10a for HEK293T and mouse testicular mRNAs, respectively.^[^ ^41^ ^]^ Uniquely mapped reads were applied for gene expression quantification using FeatureCounts v2.0.2.^[^ ^42^ ^]^ Acetylated gene expression was further analyzed using the DESeq2 R package. An adjusted P‐value of < 0.05, and fold change (FC) of acRIP/input and acRIP/IgG of > 2 and > 5 were considered statistically significant to identify ac^4^C (+) mRNAs. Fragments per kilobase of transcript per million mapped reads (FPKM) were calculated to validate gene expression and acetylation levels and normalized to gene length and sequencing depth.

To further generate the ac^4^C distribution within the target mRNAs, binned ac4C enrichments over transcripts were transformed into consistent lengths using deepTools. The ac^4^C enrichment levels are displayed as log2 ratios (acRIP/input). To clarify the peak location within the transcripts, Sambamba v0.7.1^[^ ^43^ ^]^ was used to remove duplicate reads, and the relative sizes of UTR and CDS were parsed from the annotation BED files. The ac^4^C peak positions were gathered at the intersections of the parsed MACS2 outputs. The ac^4^C sequence motifs were identified using MEME, a suite of tools for detecting motifs with representative features.^[^ ^17^ ^]^

### Ribonucleoprotein Immunoprecipitation (RIP) Assay

293T cells were harvested in polysome lysis buffer containing 100 mM KCl, 5 mM MgCl_2_, 10 mM HEPES, pH 7.0, 1% NP40, 1 mM DTT, 1X PMSF, 100 U mL^−1^ RRI, and 400 µM VRC. The supernatants were gathered after centrifugation and immunoprecipitated with anti‐FLAG affinity gels for 4 h at 4 °C. Beads were collected and rinsed eight times with NT2 buffer containing 50 mM Tris‐HCl (pH 7.4), 150 mM NaCl, 1 mM MgCl_2_ and 1% NP40. The RNA bound to the beads was extracted using the Rneasy Mini Kit (74 004; QIAGEN, Venlo, Netherlands) according to the manufacturer's instructions and reverse transcribed as described in Reverse transcription and quantitative polymerase chain reaction section. The relative abundance of cDNA was analyzed based on the fold change between the RIP groups and their corresponding input groups.

### Biotinylated RNA Pull‐Down Assay

Proteins interacting with specific RNAs were examined using a biotinylated RNA pull‐down (PD) assay following a previously reported protocol.^[^ ^44^ ^]^ Briefly, 293T cells were harvested in polysome extraction buffer containing 20 mM Tris‐HCl (pH 7.4), 100 mM KCl, 5 mM MgCl_2_, 0.5% NP40, and 1X PMSF. The supernatants were gathered after centrifugation, 1:1 (v/v) mixed with indicated designed biotinylated ribonucleotides diluted to 2 µg mL^−1^ in 2×TENT buffer containing 20 mM Tris‐HCl, pH 8.0, 2 mM EDTA, 500 mM NaCl, 1% Triton X‐100, 200 U mL^−1^ RRI and 400 µM VRC and pre‐incubated for 30 min at RT. The mixtures of cell lysates and RNAs were incubated with Streptavidin Magnetic Beads (88 817; Pierce, Appleton, WI, USA) diluted in 1X TENT buffer for 2 h at 4 °C. Bead‐bound RNAs were extracted using TRIzol reagent, and the bound ribonucleoprotein complexes were eluted with SDS sample buffer and subjected to western blot analysis.

### Statisitical Analysis

Statistical data are presented as Mean ± standard error of the mean (SEM). Most experiments included at least three independent samples and were repeated for at least three times. For RT‐qPCR and LC‐MS/MS results, data of the groups treated with the indicated siRNAs was normalized to the control groups (*siNC*). For acRIP‐qPCR and RIP‐qPCR results, the enrichments were normalized to the IgG groups of the indicated samples. Two‐tailed unpaired Student's *t*‐tests were performed to compare the results of the two indicated experimental groups. Results of which *P <* 0.05, *P <* 0.01, and *P <* 0.001 were considered statistically significant, and were presented by asterisks (*), (**), and (***), respectively. “*n.s*.” indicated not significant.

## Conflict of Interest

The authors declare no conflict of interest.

## Author Contributions

Z.‐Y.J. and Y.‐K.W. contributed as co‐first authors. H.‐Y.F. and H.‐B.W. conceived the project. H.‐Y.F., Z.‐Y.J. and Y.‐K.W. designed and analyzed the experiments. Z.‐Y.J., Y.‐K.W., Z.‐Q.D., and L.C. performed the experiments. H.‐B.W., S.‐Y.Z., Y.‐M.Z., and Y.‐S.Y. provide funding support and important reagents. H.‐Y.F. and Z.‐Y.J. wrote the paper.

## Supporting information



Supporting Information

Supporting Table

Supporting Table

Supporting Table

Supporting Table

## Data Availability

Raw RNA‐seq data was deposited on the NCBI Gene Expression Omnibus database (https://www.ncbi.nlm.nih.gov/geo/query/acc.cgi?acc=GSE252077). The GEO accession number GSE252077 were suitable with the password “srenwwmwdpijnup”.
