# Supplementary material for: PCBP1/2 and TDP43 Function as NAT10 Adaptors to Mediate mRNA ac4C Formation in Mammalian Cells
Source: Adv Sci (Weinh). 2024 Nov 18;11(47):2400133. doi: 10.1002/advs.202400133 (PMC11653668; doi:10.1002/advs.202400133)
Supplement: Supplementary file 1 — Supporting Information [file ADVS-11-2400133-s003.docx]

**Supporting figures**

**
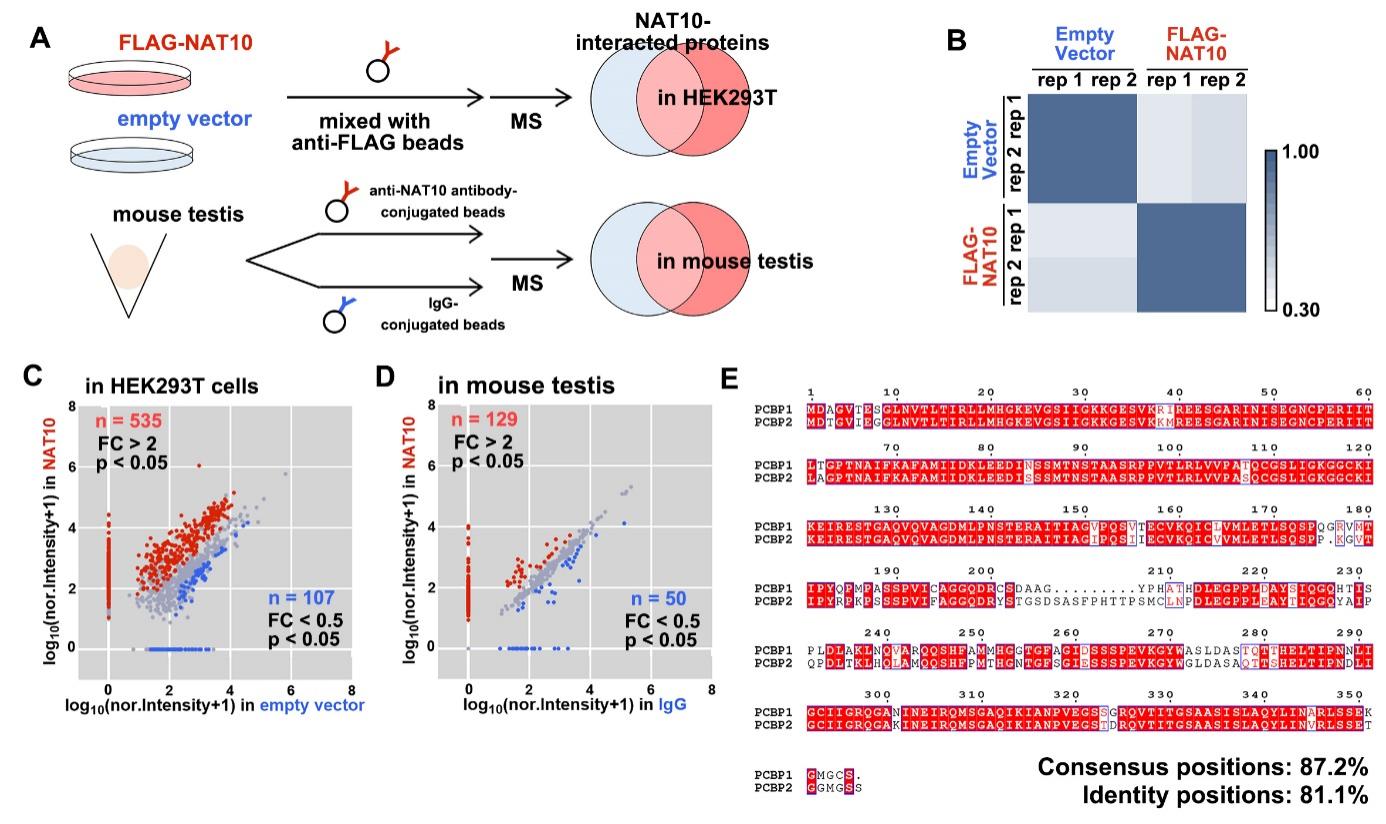
**

**Figure S1: Identifying NAT10 adaptors by proteomic analysis. A:** Schematic of affinity purification and tandem mass spectrometry analysis. HEK293T cells expressing FLAG-tagged NAT10 were lysed and subjected to immunoprecipitation with anti-FLAG beads. 293T cells transfected with empty vectors were served as the control. Wild-type (WT) mouse testis was lysed and immunoprecipitated with anti-NAT10 antibody-conjugated beads. IgG-conjugated beads were also applied as the control. Proteins enriched in the control group were removed from the NAT10 interactome. MS, mass spectrometry analysis. **B:** Heatmap of the Spearman correlation coefficients of the control, the NAT10-interactome and the control group in two replicates. Rep, replicate. **C-D:** Plot profile indicating the specific targets interacting with NAT10 in 293T cells and mouse testis, respectively. Red dots represented NAT10-specific binding proteins. FC, fold change. **E:** Alignment of the amino acid sequences between human PCBP1 and PCBP2 proteins. Amino acids with red coloring background represent position identity.

**
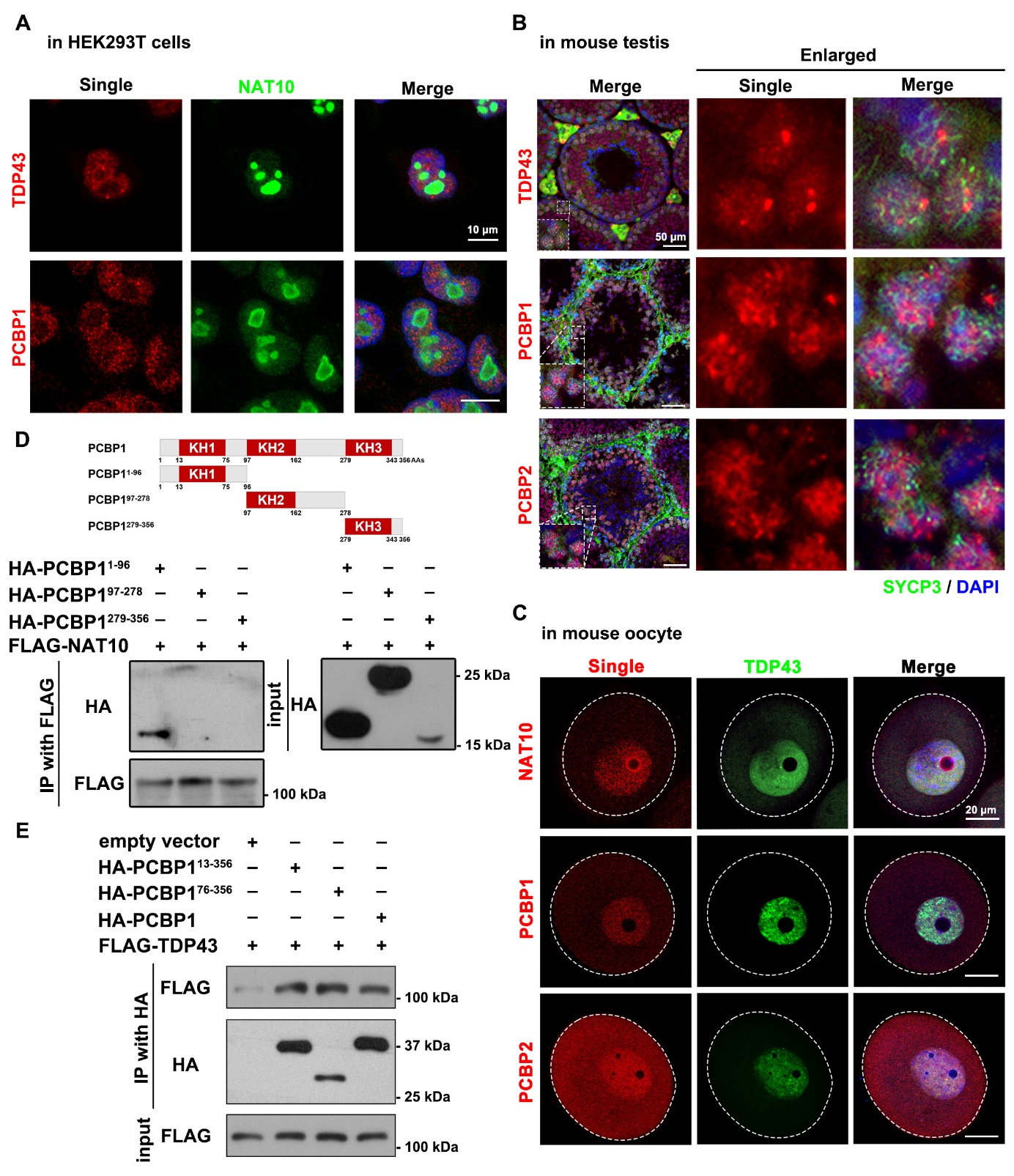
**

**Figure S2: Co-localization of PCBP/TDP43/NAT10 in the nucleus and confirmation of their interaction. A:** 293T cells stained with anti-NAT10 and the indicated candidate protein antibodies. DNA was stained with DAPI. Scale bar = 10 μm. **B:** Paraffin sections of WT testes stained with anti-SYCP3 antibody and the indicated protein antibodies. SYCP3 served as a marker for meiotic spermatocytes. PCBP1/2 and TDP43 co-expressed in spermatocytes at the pachytene stage. Scale bar = 50 μm. **C:** Mouse oocytes stained with anti-TDP43 and the indicated protein antibodies. DNA was stained with DAPI. Scale bar = 20 μm. **D:** Diagram of segmented PCBP1 constructs and co-IP results confirming the interaction between N-terminal PCBP1 (1-96 AAs) and NAT10. KH, hnRNP K homologue domain. AAs, amino acids. C-terminal PCBP1 constructs, HA-PCBP1^97-278^ and HA-PCBP1^279-356^ could not bind to NAT10. **E:** Co-IP results showing the interaction between N-terminus truncated PCBP1 with TDP43.


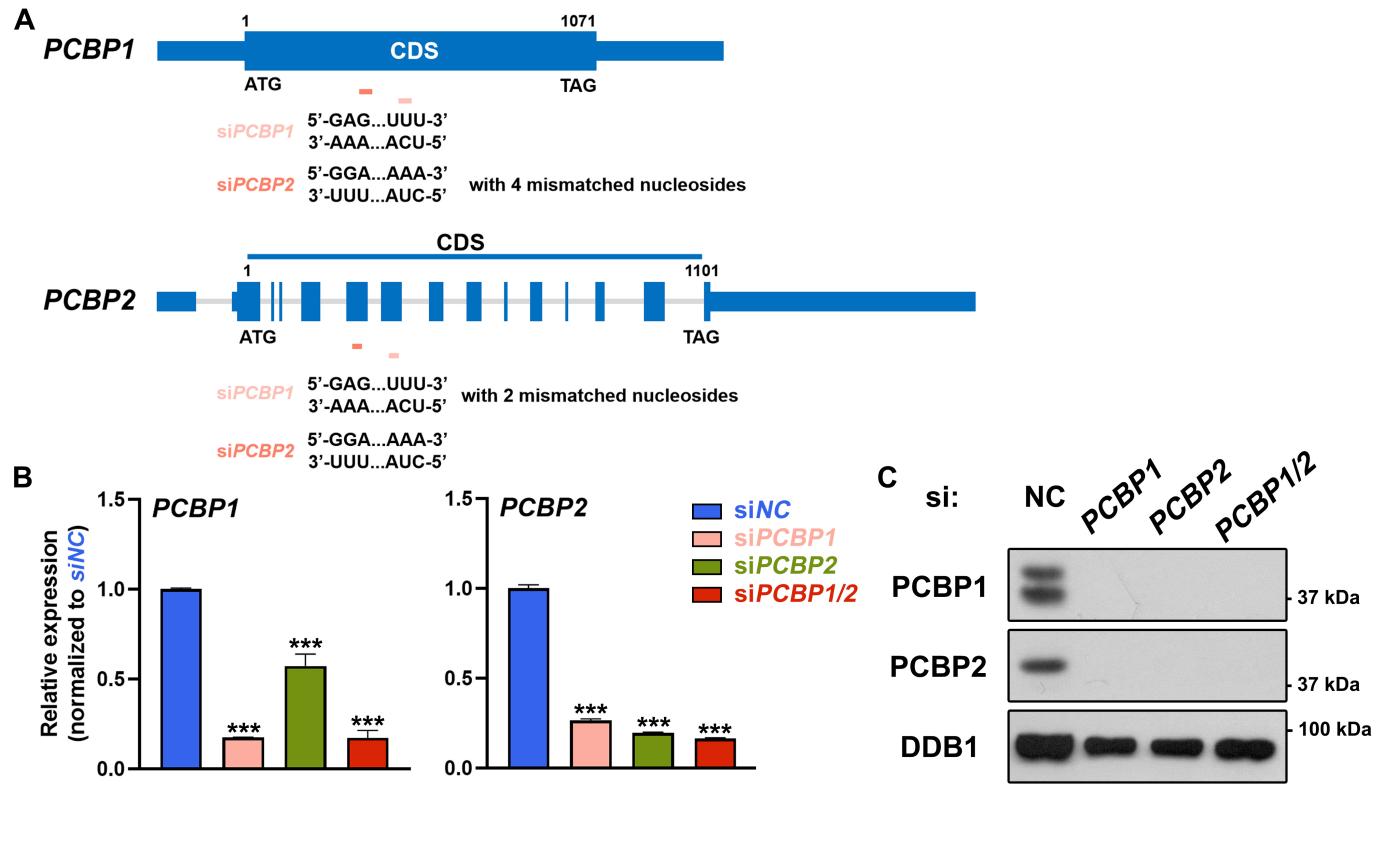


**Figure S3: Double RNA interference was applied in *PCBP1/2* knockdown according to their homology. A:** Schematic illustration of designed interference RNAs towards *PCBP1* and *PCBP2*. Targeted sites were marked below the sequences, with si*PCBP1* in light read and si*PCBP2* in red. CDS, coding sequence. **B:** RT-qPCR results showing the mRNA knockdown levels of *PCBP1/2* in *siPCBP1*, *siPCBP2* and *siPCBP1/2* groups. Gene expression levels were normalized to *GAPDH.* Mean ± SEM. ****P <* 0.001. **C:** Western bot results showing knockdown efficiency in *siPCBP1*, *siPCBP2* and *siPCBP1/2* groups. DDB1 was blotted as the loading control.


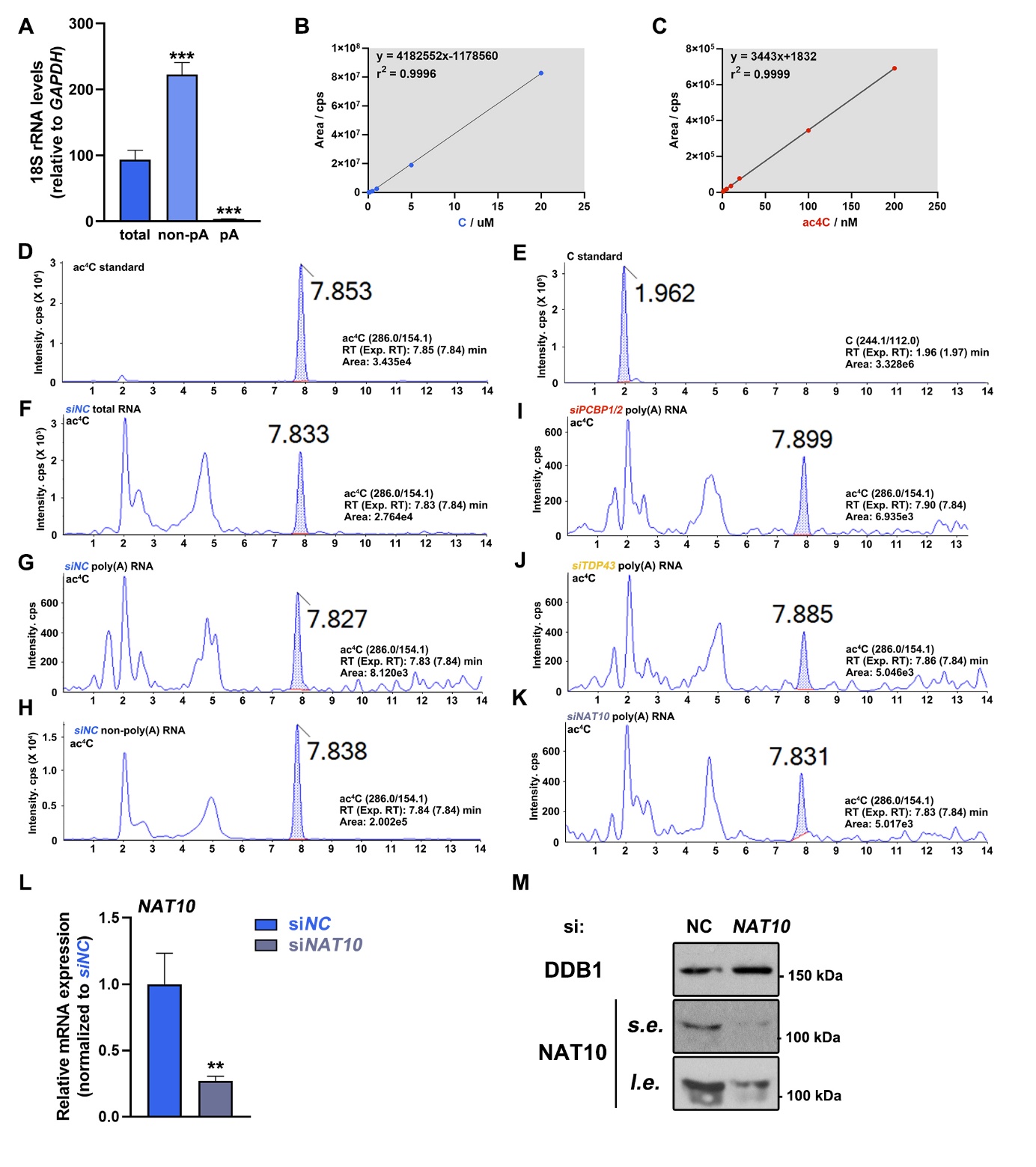


**Figure S4:** **Evaluation of PCBP1/2 and TDP43 effects on mRNA ac^4^C modification. A:** RT-qPCR results detecting 18S rRNA level to examine RNA purity. Expression of 18S rRNA level was normalized to *GAPDH*. Total, total RNA. pA, poly(A). non-pA, non-poly(A) RNA. Mean ± SEM. ****P <* 0.001. **B-C:** Calibration curves applied for cytidine (**B**) and N-4-acetylcytidine (**C**) quantification in LC-MS/MS analysis. **D-K:** LC-MS/MS chromatograms for quantification of ac^4^C in ac^4^C standards (**D**), C in C standards (**E**), ac^4^C in total RNA, poly(A) RNA and non-poly(A) RNA in the control group (*siNC*, **F**, **G** and **H**, respectively) and ac^4^C in poly(A) RNA upon *PCBP1/2*, *TDP43* and *NAT10* knockdown (**I**, **J** and **K**, respectively). **L:** RT-qPCR results showing *NAT10* knockdown efficiency. Mean ± SEM. ***P <* 0.01. **M:** Western blot results showing *NAT10* depletion efficiency. DDB1 was blotted as a loading control. *s.e.*, short exposure. *l.e.*, long exposure.


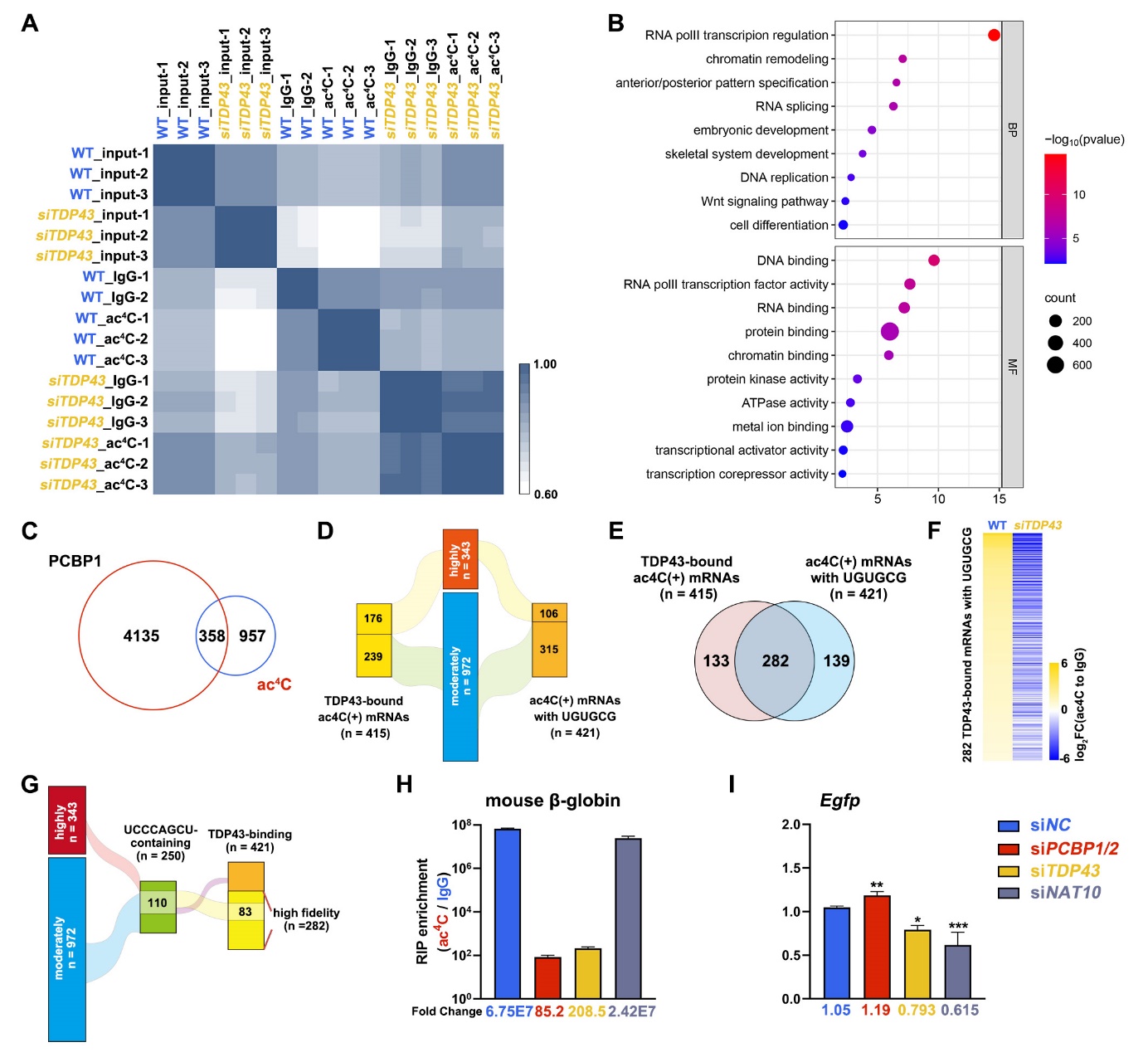


**Figure S5: Mapping of ac4C-containing poly(A) RNAs in HEK293T cells by acRIP-seq. A:** Heatmap of the Spearman correlation coefficients of the input, the IgG-enriched and the ac^4^C-enriched transcripts in WT and *siTDP43* groups (triplicates in input of both groups, duplicates in WT IgG groups, triplicates in *siTDP43* IgG groups and triplicates in both ac^4^C-enriched groups, respectively). The color key in blue from dark to light indicated the value of correlation coefficient from high to low. **B:** Bubble plot indicating the gene ontology (GO) enrichment analysis of the defined WT ac^4^C(+) mRNAs in terms of biological pathway (BP) and molecular function (MF). The color key from red to blue indicated the significance from high to low. **C:** Overlap between ac^4^C-harbored and PCBP1 bound transcripts. **D:** A Sankey diagram depicting the possible TDP43-bound ac^4^C(+) mRNAs. Reported TDP43-bound mRNAs and mRNAs containing UGUGCG, a known TDP43-recognizing motif, are presented. **E:** A Venn diagram showing the overlapped targets of reported TDP43 targets and ac^4^C(+) mRNAs containing UGUGCG. These mRNAs are further designated high fidelity targets. **F:** A heatmap showing the decreased acetylation of high fidelity TDP43 targets upon *TDP43* depletion. FC, fold change. **G:** A Sankey diagram depicting the high fidelity TDP43 mRNAs harboring UCCCAGCU, the predicted ac^4^C motif. **H-I:** Validation of RT-qPCR results of spiked-in ac^4^C(+) mouse β-globin and ac^4^C(–) *Egfp* recovered from acRIP. Mean ± SEM. **P* < 0.05, ***P* < 0.01, ****P* < 0.001.


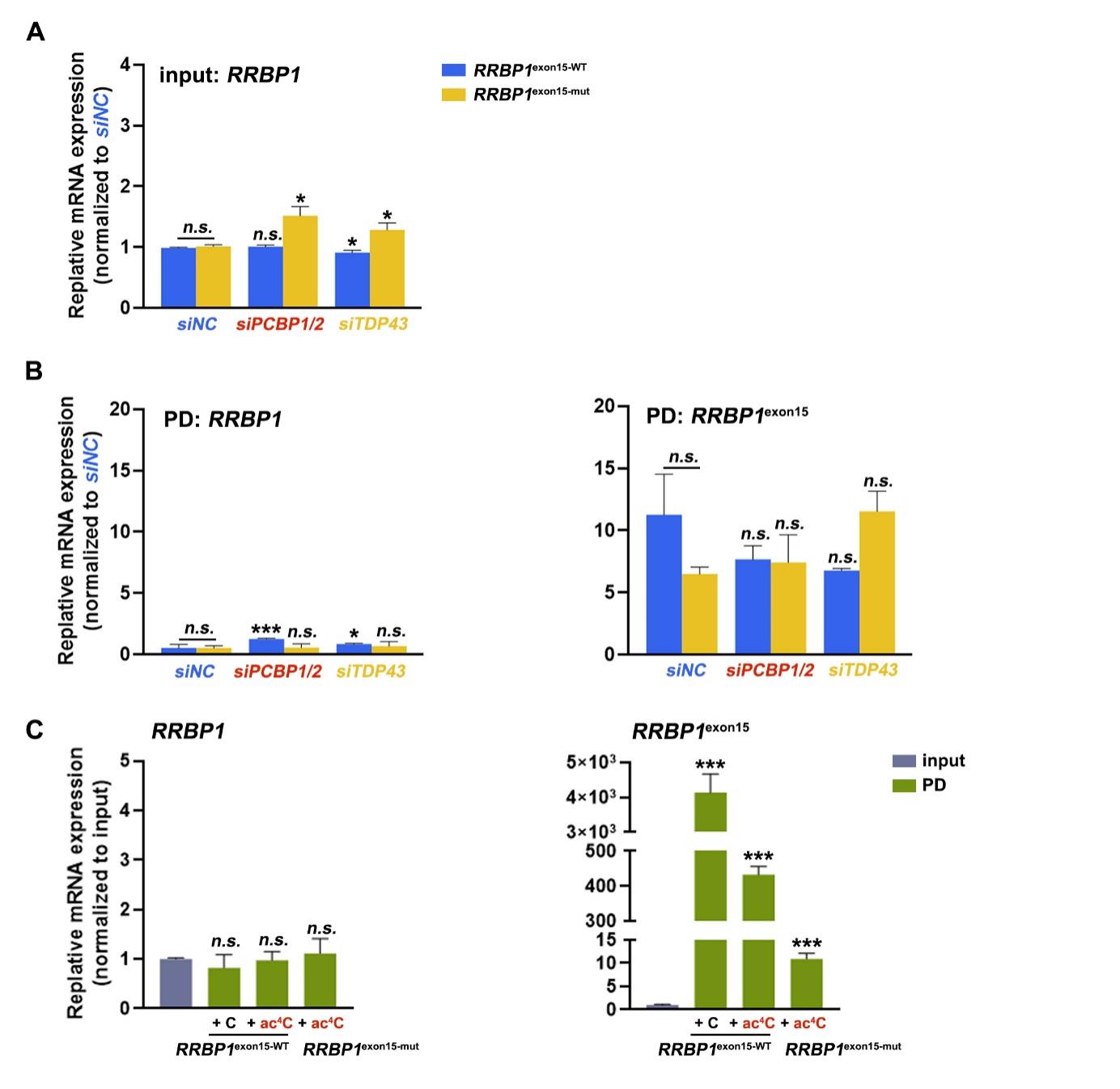


**Figure S6: Validation of affinity between *RRBP1*^exon15^ and NAT10/PCBP/TDP43 complex. A-B:** RT-qPCR results showing incorporation of *in vitro* transcribed WT and mutated *RRBP1*^exon15^ oligonucleotides did not alter overall *RRBP1* expression level and the indicated oligonucleotides were enriched in RNA pull down. PD, pull down. Mean ± SEM. **P* < 0.05, ****P* < 0.001. *n.s.* not significant. **C:** RT-qPCR results showing incorporation of *in vitro* transcribed *RRBP1*^exon15^ oligonucleotides with or without ac^4^C did not alter overall *RRBP1* expression level and the indicated oligonucleotides were enriched in RNA pull down. Mean ± SEM.


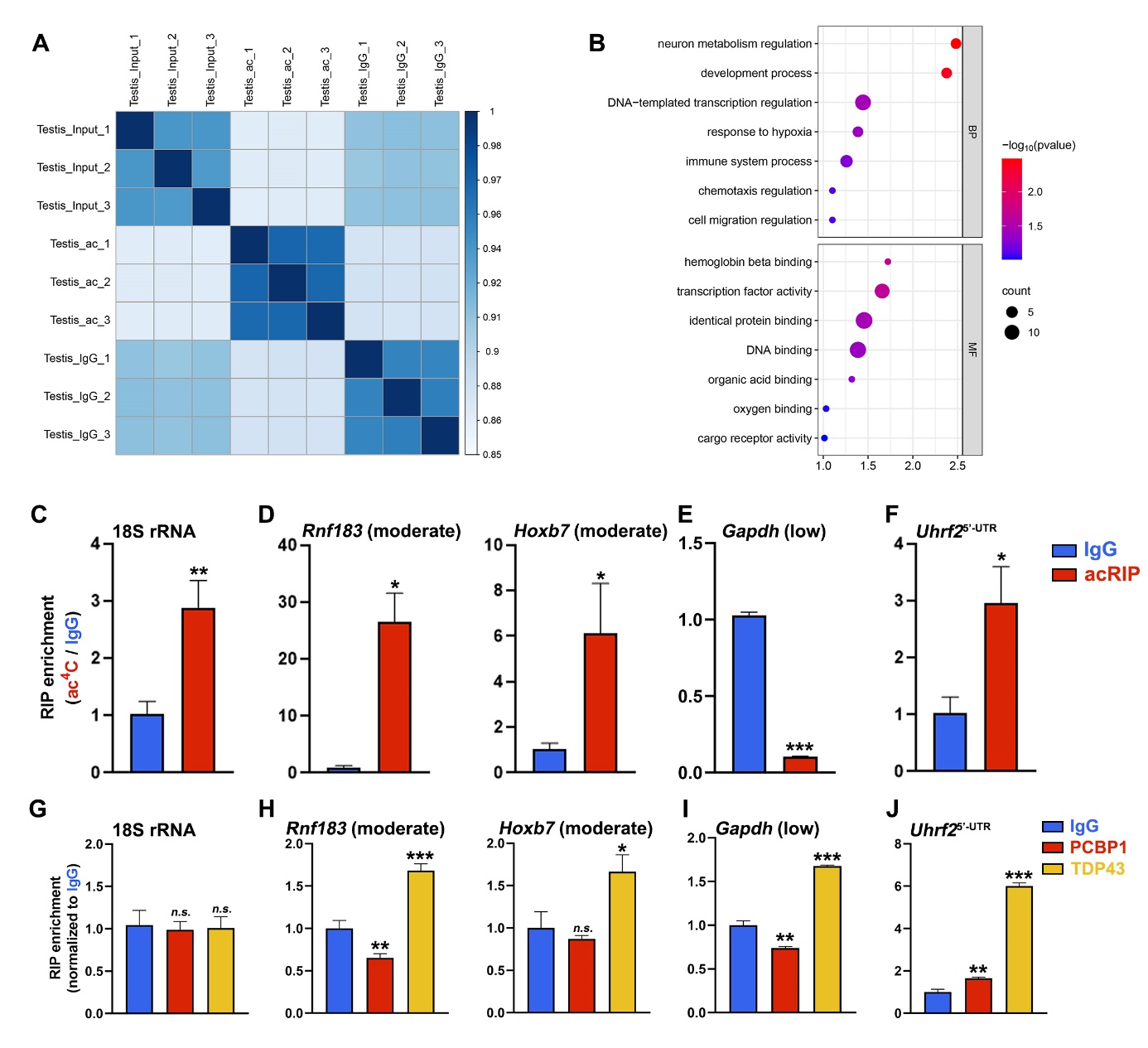


**Figure S7: Validation of NAT10/PCBP/TDP43-directed N-4-acetyltransferation effect in mouse testisticular mRNAs. A:** Heatmap of the Spearman correlation coefficients of the input, the IgG-enriched and the ac^4^C-enriched transcripts in mouse testis (triplicates in all groups). The blue color key from dark to light indicated the value of correlation coefficient from high to low. **B:** Bubble plot indicating the GO enrichment analysis of the defined WT mouse testis ac^4^C target mRNAs in terms of BP and MF. The color key from red to blue indicated the significance from high to low. **C:** RT-qPCR results showing 18S rRNA was successfully enriched by acRIP-seq in testis mRNA. Mean ± SEM. ***P* < 0.01. **D-F:** acRIP-qPCR results showing ac^4^C(+) and ac^4^C(–) mRNA varied in acetylation abundance, discarding the peak localization. Mean ± SEM. **P* < 0.05. **G-J:** RT-qPCR following endo-RIP results showing ac^4^C(+) and ac^4^C(–) mRNA presented divert affinity to PCBP1 and TDP43. Mean ± SEM. ****P* < 0.001.
