# Supplementary material for: PCBP1/2 and TDP43 Function as NAT10 Adaptors to Mediate mRNA ac4C Formation in Mammalian Cells
Source: Adv Sci (Weinh). 2024 Nov 18;11(47):2400133. doi: 10.1002/advs.202400133 (PMC11653668; doi:10.1002/advs.202400133)
Supplement: Supplementary file 2 — Supporting Table [file ADVS-11-2400133-s005.pdf]

ADVANCED SCIENCE
Open Access

Supporting Information

for *Adv. Sci.*, DOI 10.1002/advs.202400133

PCBP1/2 and TDP43 Function as NAT10 Adaptors to Mediate mRNA ac^{4}C Formation in Mammalian Cells

Zhi-Yan Jiang, Yu-Ke Wu, Zuo-Qi Deng, Lu Chen, Yi-Min Zhu, Yuan-Song Yu, Hong-Bo Wu and Heng-Yu Fan**

Table S1. ac4C(+) mRNAs in WT 293T cells

Gene Syı	FPKM+1_inpı	FPKM+1_IgG	FPKM+1_ac4(	p-value_ac4C ı	p-value_ac4C ı	Cluster
SUZ12P	5.776238782	0.186301921	10.27515609	6.28606E-06	0.000676	highly acetylated
CENPE	2.97314028	2.082916607	114.4983817	2.45769E-08	2.65301E-06	highly acetylated
YJEFN3	1.754191763	0.570063103	22.61321614	1.83261E-05	0.001673986	highly acetylated
SNORD6	5.393459991	0.487619694	19.20260386	0.000587242	0.008141538	highly acetylated
GOLGA4	4.663918267	2.991406604	114.0990268	7.92558E-06	0.000884329	highly acetylated
SNORD3	8.995916769	0.284694261	10.00470742	2.17204E-06	0.011310814	highly acetylated
CNFN	31.52643863	0.063761052	2.236730055	0.000206843	0.00138106	highly acetylated
EIF5B	12.76881705	13.02601755	444.554359	1.13382E-05	0.001349198	highly acetylated
TOMM4(	45.03613447	0.084854961	2.549840185	1.86768E-05	0.000685789	highly acetylated
LOC1001	3.005262375	0.407106514	12.16471371	0.000229476	0.00530047	highly acetylated
BOD1L	1.750756239	2.370271668	69.28217765	1.7489E-06	0.00032613	highly acetylated
NSUN5P	7.841370847	0.191350327	5.403298289	5.48831E-06	0.000371681	highly acetylated
NEXN	2.116214803	1.257922264	35.04392234	7.07197E-05	0.00276596	highly acetylated
CEP290	2.778168609	3.323790503	90.59314904	1.08669E-05	0.001367696	highly acetylated
AKAP9	2.50014365	2.408470262	65.37406546	1.10921E-06	0.000258434	highly acetylated
PCSK1N	45.50803451	0.11521233	2.959525899	0.000134227	0.001774707	highly acetylated
CENPF	5.785839563	3.08531474	74.06511041	1.73997E-06	0.00047292	highly acetylated
UACA	5.827518178	1.052611484	25.18030536	2.57708E-06	0.000428224	highly acetylated
ANKRD1	2.585704047	6.217440823	145.9774637	4.62298E-06	0.000848602	highly acetylated
TMSB4X	6.557436376	0.15249862	3.32168911	0.002048776	0.010155785	highly acetylated
GOLGB1	2.978196791	3.612258112	74.84060287	5.19138E-06	0.000925888	highly acetylated
GOLGA8	3.194206384	1.366919606	28.10854772	1.20305E-05	0.000602827	highly acetylated
ANKRD1	1.72495745	1.188057524	24.01197785	3.50693E-06	0.000347295	highly acetylated
HES7	2.250720729	0.557288048	11.12894174	1.48554E-05	0.001193599	highly acetylated
NSRP1	14.23506814	6.192857296	122.4911894	1.54815E-06	0.000541955	highly acetylated
NEAT1	3.444180148	1.447798502	28.59298605	6.60794E-07	0.000169826	highly acetylated
C10orf2	6.422286922	0.165125617	3.253037643	8.52275E-06	0.000524544	highly acetylated
HOXD9	1.787289696	0.755353565	14.87585892	0.000112092	0.003578083	highly acetylated
LOC1005	8.654867628	0.130971613	2.356122467	0.000644699	0.003427805	highly acetylated
LOC1001	6.076459659	0.19366107	3.45638678	6.87629E-05	0.00062863	highly acetylated
PCNT	1.936354367	1.403068205	24.92385048	2.34154E-07	3.52707E-05	highly acetylated
TMEM5:	11.83723042	0.127156269	2.228869071	0.002983051	0.007238483	highly acetylated
SLC6A8	5.583895614	0.233153765	4.070741974	0.000175667	0.002631202	highly acetylated
SPINK5	1.145632178	1.714687604	29.78581228	2.79823E-06	0.000429125	highly acetylated
PAQR6	1.926000949	0.519210544	9.005563751	0.000950446	0.011458986	highly acetylated
RBBP6	3.901807565	2.646953104	45.85147434	9.77162E-06	0.000895119	highly acetylated
NUMA1	2.927288447	1.452136091	24.47001444	7.53764E-05	0.003690044	highly acetylated
LINC001	1.614640851	1.054982665	17.67297645	8.81561E-06	0.001134773	highly acetylated
MIR324	1	1	16.5152755	0.015873209	0.056687908	highly acetylated
ANKRD1	1.663769063	1.973018266	32.28879495	1.79043E-06	0.000548008	highly acetylated
TRIM56	1.295492357	1.888082672	30.65170467	1.53882E-07	2.94787E-05	highly acetylated
ATRX	4.567042186	2.698085673	43.46543414	2.19892E-06	0.000596019	highly acetylated
KLHL17	3.557003237	0.281135533	4.386423718	3.07997E-05	0.001496224	highly acetylated
IRF2BP1	2.849265412	0.365732016	5.481524444	4.25741E-05	0.001920336	highly acetylated
FAM131(	2.944821283	0.42600869	6.364434454	0.000136053	0.003874534	highly acetylated
FXR2	4.467501747	0.45729749	6.811504154	6.40807E-05	0.001958922	highly acetylated
ZC3H13	5.128005663	18.71114569	272.0408044	5.67918E-07	0.000158444	highly acetylated
EXOSC6	3.938329437	0.402130563	5.796311412	4.79138E-07	0.012809621	highly acetylated
ATN1	3.470139837	0.550853999	7.880988321	1.3505E-05	0.000713706	highly acetylated
RRBP1	5.680146604	4.612757643	64.47927548	6.56611E-08	6.35998E-06	highly acetylated
ANKRD1	4.362739396	1.479230431	20.6384132	2.81292E-06	0.000348644	highly acetylated
LOC6456	24.15644187	0.170921144	2.377527157	0.000583037	0.003529649	highly acetylated
RP9P	4.5103994	0.361584456	4.896982491	3.64632E-06	5.75588E-05	highly acetylated

PPIG	17.51676968	3.736992161	50.20571968	7.37843E-06	0.001204403	highly acetylated
HEXDC	2.07347699	0.482281696	6.466462162	0.002325894	0.017094512	highly acetylated
B4GALN	1.652880966	1.004498054	13.44087071	3.99141E-06	0.000550133	highly acetylated
CACNG8	1.112352771	1.324725272	17.66671288	1.82691E-08	3.68905E-05	highly acetylated
9-Mar	2.330729947	0.444981349	5.913174953	7.26246E-06	0.000733902	highly acetylated
APOA1	2.343283696	0.479482793	6.341231924	0.00040336	0.006055148	highly acetylated
ASPHD1	1.906738573	0.638750372	8.412952267	4.15104E-06	0.000547637	highly acetylated
BDP1	2.588102002	2.031736276	26.6566838	2.45944E-05	0.001241125	highly acetylated
MAPK15	4.016487439	0.560235175	7.291598075	0.000272298	0.004518612	highly acetylated
FLT3LG	5.6746927	0.226419578	2.939897283	8.6736E-05	3.23604E-05	highly acetylated
ANKRD2	1.405667274	2.706034136	35.11560134	3.44116E-07	0.000169348	highly acetylated
SPHK1	5.403446403	0.201682946	2.611952411	0.000242219	0.002500704	highly acetylated
FAM89B	2.987400146	0.634126076	8.060326361	0.000117438	0.003628028	highly acetylated
CEP250	2.107306536	3.638073861	46.04630988	2.41709E-07	0.000135234	highly acetylated
SCARNA	2.945901815	0.339454626	4.294614192	0.004811256	0.019501711	highly acetylated
LOC1001	5.593714781	0.178772075	2.257611765	0.008442228	0.014995519	highly acetylated
CAHM	1.869616891	0.534868937	6.713888055	6.03783E-05	0.002250763	highly acetylated
PUS1	6.195869422	0.377933979	4.738201692	6.34311E-07	3.7034E-05	highly acetylated
C1QL1	2.222405972	0.516459554	6.264196796	1.46484E-06	0.000312193	highly acetylated
GPR137	5.836346367	0.191698837	2.300185141	5.84679E-05	0.000323301	highly acetylated
LENG8	4.26351073	0.272915564	3.273860628	0.000283109	0.003773411	highly acetylated
ARID4A	2.335204085	2.158840597	25.87447563	6.04113E-08	8.54025E-06	highly acetylated
LUC7L	9.187650434	0.414482074	4.945696695	4.7289E-06	0.000623002	highly acetylated
CHST14	1.748175977	0.572024792	6.792416366	0.00017461	0.004599165	highly acetylated
PNN	32.10118812	1.923235362	22.53427668	5.89993E-06	0.000848804	highly acetylated
KAT2A	7.035757337	0.961050763	11.23640854	5.68852E-05	0.002895608	highly acetylated
ATHL1	4.667645348	1.261209454	14.71133667	7.80623E-06	0.000306353	highly acetylated
AHNAK	1.58015546	1.442125836	16.71946308	3.36489E-08	2.21646E-05	highly acetylated
CCDC64	4.266899763	0.234362196	2.701203973	0.000503819	0.004309706	highly acetylated
FBXL6	2.77631056	0.382381715	4.380823683	0.000226017	0.00398756	highly acetylated
ERICH1	10.77962344	0.696547837	7.976340523	5.70022E-07	2.075E-05	highly acetylated
SNORD6	3.623269161	0.275993849	3.119810838	0.022387392	0.03863296	highly acetylated
UFSP1	3.959046752	0.252586055	2.849172259	6.33859E-05	0.001070155	highly acetylated
SNORA1	2.993284375	0.334081188	3.711840532	0.049738789	0.065965186	highly acetylated
CEP164	2.111408503	1.420446192	15.62025904	1.49093E-06	0.000526773	highly acetylated
VPREB3	2.288201407	0.437024467	4.787692514	0.000785949	0.006137398	highly acetylated
FHAD1	3.277290253	0.589992352	6.418618218	4.68271E-06	0.00065097	highly acetylated
PABPC1	3.965370968	0.761921301	7.928723054	5.47618E-05	0.001422525	highly acetylated
LOC4409	11.98038246	0.220871633	2.279717078	3.61483E-06	0.01434144	highly acetylated
HOXA6	2.602236063	0.38428489	3.954484466	1.07058E-05	0.000115076	highly acetylated
MIR940	1.680538633	0.595047314	6.108283932	0.003915377	0.02267422	highly acetylated
PITPNA	5.751848925	0.426227737	4.37395598	1.50309E-05	0.000719859	highly acetylated
DDX51	1.958748788	0.709956781	7.238065058	6.19853E-06	0.000826983	highly acetylated
CECR5-A	5.12975814	0.263971925	2.675845027	8.1725E-05	0.000575155	highly acetylated
CROCCF	8.734034924	0.281988206	2.846306388	0.000105695	0.000881254	highly acetylated
MKI67	4.039185695	3.032152939	30.54006274	1.46072E-07	0.000174296	highly acetylated
TBKBP1	2.091762384	0.730201428	7.339398672	5.79868E-05	0.001877955	highly acetylated
ZNF692	8.489576126	0.21306263	2.140140182	0.000104602	0.000250916	highly acetylated
EMILIN2	2.218463629	0.562922736	5.653304323	0.000115132	0.001557801	highly acetylated
ADRA2C	2.118091039	0.864221512	8.644349293	1.33295E-05	0.001180381	highly acetylated
LOC2027	9.46723944	0.261806637	2.561661214	0.000323038	0.002593323	highly acetylated
PWWP2I	1.796773605	0.626294004	6.003690699	7.67118E-06	0.00071101	highly acetylated
C2orf77	2.167367256	0.508543765	4.861556106	2.23594E-05	0.000502332	highly acetylated
TCF7L1	1.984258693	0.988189553	9.36237204	1.43054E-05	0.001060441	highly acetylated
C22orf46	1.707989147	3.447458711	32.56012623	2.68284E-07	0.000143071	highly acetylated

CCDC18	3.00102801	2.69371936	25.4367007	1.1861E-07	8.72527E-06	highly acetylated
PROCA1	2.12402931	1.13248466	10.65497052	0.000181695	0.001929353	highly acetylated
ZNF688	3.174777962	0.314982658	2.939051092	9.02194E-05	0.000803927	highly acetylated
CCDC88	3.240846254	1.561179699	14.5577942	4.53398E-07	0.000143588	highly acetylated
WDR85	25.01461795	0.240001889	2.225016084	4.75759E-06	0.000340521	highly acetylated
PAGE2B	1.398362216	0.715122297	6.590372455	0.00240185	0.018828949	highly acetylated
RRS1	9.477302034	0.520880364	4.794935352	1.27495E-05	0.00089367	highly acetylated
ZDHHC1	1.57494641	0.686415784	6.303423575	3.59202E-06	0.000209051	highly acetylated
H2AFX	15.58355503	0.52863305	4.849440139	6.0861E-05	0.000627033	highly acetylated
MAN2C1	8.423492681	0.267719397	2.440431592	0.000204024	0.00053023	highly acetylated
LINC001	1.700592409	0.58803038	5.262966562	0.010125844	0.033711069	highly acetylated
C11orf96	1.895748298	3.662802865	32.69040429	6.28849E-06	0.001187758	highly acetylated
NAGPA	4.075718382	0.245355519	2.18188637	0.001216256	0.004873243	highly acetylated
NEFM	20.70867245	4.864517364	43.0573414	3.84015E-05	0.0026038	highly acetylated
PANX2	1.572402986	0.658685683	5.82304593	2.13244E-05	0.001352753	highly acetylated
LOC3899	2.06228507	0.574923805	5.075615677	2.41755E-05	0.000220396	highly acetylated
C1orf216	1.86245359	0.576540226	5.083325083	0.001173977	0.011701536	highly acetylated
SLTM	6.177003474	4.535327248	39.95939915	3.09961E-06	0.00071196	highly acetylated
ZNF703	3.513669787	1.245381413	10.94796762	3.17341E-05	0.001805491	highly acetylated
MIR10B	2.016764065	0.495843821	4.349737498	0.016994449	0.044764165	highly acetylated
TCEA2	3.696960209	0.417379952	3.647156597	6.86581E-06	0.000110011	highly acetylated
CNTRL	2.598615707	1.518418528	13.18742619	2.52443E-06	0.000629529	highly acetylated
PLBD2	3.033993011	0.379379708	3.283188727	0.000752994	0.006582437	highly acetylated
PDIA2	1.429195296	0.958733645	8.261088191	0.000446496	0.00612773	highly acetylated
SNORD1	1.23977873	0.806595545	6.940983049	0.005205876	0.0283622	highly acetylated
MGC161	1.554983633	0.824464136	7.071035806	6.16988E-06	0.000294347	highly acetylated
ZNF605	1.7056434	0.586289021	5.02669455	5.12712E-07	0.000184294	highly acetylated
PALM3	1.247763591	11.25049443	96.24040391	2.77904E-08	4.43207E-06	highly acetylated
SYNE2	1.694365633	1.066779117	9.09945575	1.65713E-06	0.000407734	highly acetylated
ADCK2	6.690624981	0.492274719	4.197526367	0.000100674	0.00224537	highly acetylated
SHISA8	1.243677957	2.414543197	20.55596795	1.16537E-05	0.001575986	highly acetylated
FAM50B	2.736722314	0.365400609	3.102381877	0.000659857	0.006192446	highly acetylated
TMEM17	1.359678421	0.824557794	6.989684797	0.000203233	0.00411205	highly acetylated
CRYGD	3.342175172	0.527131868	4.409760618	0.001373805	0.01078288	highly acetylated
GCC2	3.879554661	2.96376997	24.74826154	8.26329E-06	0.001197144	highly acetylated
FUS	31.28651811	0.475048543	3.958579665	1.89506E-05	0.001267174	highly acetylated
LPAR2	1.320066319	0.89354844	7.433576471	1.57924E-05	0.001232507	highly acetylated
PDX1	1.378115244	0.72562872	6.024010883	5.81734E-05	0.002727629	highly acetylated
PRKCG	1.400816213	0.739164671	6.106130319	4.76088E-06	0.000593442	highly acetylated
SURF1	27.10776266	0.322736767	2.661202024	0.000230383	0.000887691	highly acetylated
PILRB	4.309399731	0.415805898	3.428363793	3.25443E-05	0.000130603	highly acetylated
FAM100	9.222634363	0.46536762	3.786814136	5.80643E-05	0.001918769	highly acetylated
PNISR	18.0768465	4.724781833	38.33725521	2.50226E-06	0.00079673	highly acetylated
TIGD5	1.622398131	0.725675808	5.871165647	1.77935E-05	0.000853222	highly acetylated
CPNE7	2.174234199	0.864774689	6.94392938	5.92829E-08	0.001236289	highly acetylated
TAF6L	1.917991658	0.521378701	4.172862487	0.000348448	0.005833996	highly acetylated
DBP	4.6551603	0.443276241	3.530019515	9.67909E-05	0.000441641	highly acetylated
LOC4404	10.92641572	0.258261955	2.055591405	2.98921E-05	0.008042574	highly acetylated
ATG16L	3.67724999	0.285994237	2.272205896	2.86237E-05	0.000263994	highly acetylated
CAMSAI	1.362354844	0.945125738	7.465805261	5.59692E-06	0.000146562	highly acetylated
CDR2L	3.393443215	0.303930811	2.391774684	0.000843126	0.003849134	highly acetylated
PPP1R16	6.347721493	0.406239581	3.188862096	0.000152829	0.00121527	highly acetylated
STX4	18.87286957	0.330314796	2.5848172	1.55356E-05	0.00049729	highly acetylated
BTBD6	5.571289598	1.407649193	10.93485793	1.93544E-06	7.0796E-05	highly acetylated
PRKAR1	3.069716379	0.466178839	3.586658249	1.16383E-05	0.000112268	highly acetylated

XCL1	3.581418714	0.279218958	2.142108043	0.000610229	0.003120058	highly acetylated
SMOC1	2.33034097	0.816358006	6.237178751	2.94776E-06	4.95857E-05	highly acetylated
SCARNA	1.132052054	0.883351606	6.742307894	0.000982636	0.012214863	highly acetylated
XYLT2	3.563933711	0.34121036	2.604304562	4.52781E-06	0.000240653	highly acetylated
U2AF2	5.175731783	2.147421711	16.34549562	7.62789E-07	0.000219761	highly acetylated
DPH3P1	1.199306682	0.833815082	6.317671161	0.000399962	0.00778121	highly acetylated
CCL27	1.689455432	0.591906706	4.484705287	0.001650155	0.012315061	highly acetylated
SPIRE2	2.005315229	0.531150583	4.021030875	0.0089242	0.030869057	highly acetylated
SLC25A2	6.26477171	0.45063048	3.403787021	9.43384E-05	0.000386466	highly acetylated
SHC2	2.286849923	1.391616786	10.46195994	9.23171E-06	0.000213227	highly acetylated
LIN7B	8.743452911	0.277541798	2.075411152	0.006139512	0.007800851	highly acetylated
GOLGA2	4.536498004	4.713466788	34.96990799	6.91321E-06	0.001105044	highly acetylated
CAMK2N	1.140804164	0.948013603	6.932017292	0.000321141	0.006362103	highly acetylated
MCOLN3	2.830569264	0.367025186	2.678060792	0.000777467	0.005314791	highly acetylated
C17orf56	2.678017167	0.408754608	2.977465604	0.000114536	0.002354617	highly acetylated
KIF7	2.427133434	0.55899747	4.065925452	0.000116067	0.002934675	highly acetylated
SNORA4	1.306546872	0.765376292	5.566951474	0.001378689	0.011838851	highly acetylated
FAM176	2.160252277	2.18105393	15.84263099	0.00031997	0.006146251	highly acetylated
IRX3	1.578932267	1.13896471	8.258814639	2.3665E-05	0.001766707	highly acetylated
MAST2	2.793290104	0.3922523	2.836814516	0.000226988	0.002981034	highly acetylated
HOXA4	3.426867342	0.581619602	4.202161026	2.18282E-05	0.000234264	highly acetylated
UCN	1.371378073	0.729193517	5.213422789	0.000107127	0.003569873	highly acetylated
HNRNPU	3.191008122	1.923830237	13.68275869	1.73646E-06	0.000453966	highly acetylated
CACNB3	3.722039411	0.286215544	2.034330835	3.12279E-06	1.06331E-05	highly acetylated
NIN	1.806635045	1.103390773	7.759540557	5.61275E-07	3.75191E-05	highly acetylated
NRN1L	3.047473337	0.3855975	2.708659789	0.014501351	0.026815265	highly acetylated
HOTAIR	3.763726479	0.313813662	2.195540058	0.001112161	0.00164742	highly acetylated
NANOS1	1.514899429	0.736944684	5.143835386	2.43442E-05	0.001686709	highly acetylated
SOX18	1.190067941	0.84028816	5.856506489	3.65857E-06	0.000591739	highly acetylated
FAM129	2.695459082	0.499741785	3.466683449	0.002350254	0.012806302	highly acetylated
SH2B1	3.22993069	0.369225201	2.548208249	0.000585377	0.003526898	highly acetylated
ENO3	3.516938717	0.304976844	2.100314275	0.00012012	0.000360896	highly acetylated
TWIST1	3.909728172	0.668791302	4.599270798	6.18292E-05	0.000755642	highly acetylated
LTB4R	1.503807327	1.070024935	7.348202143	3.22172E-05	0.002334794	highly acetylated
LLGL2	3.090361168	0.424373179	2.907657239	0.000224244	0.000980296	highly acetylated
GRASP	1.056210975	1.001096488	6.857242241	1.41938E-06	8.08713E-05	highly acetylated
CCAR1	17.32301539	2.385724524	16.28307156	1.63997E-06	7.58134E-05	highly acetylated
TFEB	2.054068711	0.5087024	3.469862695	0.000344114	0.004597409	highly acetylated
TRIM47	4.000029593	0.332258623	2.245769171	0.000265534	0.000623498	highly acetylated
KCNJ11	2.07735567	0.748366591	5.028325423	0.000155052	0.0011502	highly acetylated
USP27X	1.310597763	1.157105649	7.72532291	0.000124426	0.002600272	highly acetylated
D2HGDH	2.841154631	1.812246028	12.00633281	6.00555E-05	0.001927785	highly acetylated
CDC16	9.181704097	0.39005388	2.582799801	1.38836E-05	0.008773326	highly acetylated
DCAF15	4.00964774	0.310608835	2.045388362	0.002326792	0.003428591	highly acetylated
VWA1	1.522077814	0.664444549	4.359759071	4.62109E-07	0.000179721	highly acetylated
MIR326	1	1	6.531731249	0.001372107	0.01555136	highly acetylated
SCNN1D	1.455358534	0.807116832	5.270067638	1.52944E-05	0.000356746	highly acetylated
PHF19	5.402035445	0.321730925	2.097621155	5.02751E-06	6.93111E-05	highly acetylated
BLOC1S	2.186446215	0.704417492	4.589296858	3.52907E-05	0.001930126	highly acetylated
CCDC15	2.781657774	0.497019186	3.220837337	1.99998E-05	0.000895436	highly acetylated
LOC904	1.088112096	0.919022961	5.953338884	0.001541361	0.015978025	highly acetylated
ATP6AP	2.477063598	0.403703805	2.612893242	0.012548198	0.026817465	highly acetylated
TRNP1	1.331438294	1.341014901	8.659792398	9.34898E-06	0.000582885	highly acetylated
BREA2	1.150572721	0.869132373	5.61036975	0.00661087	0.033280901	highly acetylated
IQGAP3	4.129402937	0.375070078	2.416099877	5.56533E-07	0.009263685	highly acetylated

SSBP4	7.515384612	0.389402993	2.50228515	0.000456383	0.000584423	highly acetylated
CLDND2	6.050728012	1.571633491	10.02586755	0.000154707	0.001831283	highly acetylated
AVEN	2.04265848	1.220517221	7.781831722	3.64574E-06	0.000346771	highly acetylated
ZNF839	2.734411274	0.450625235	2.852990768	2.88155E-05	0.00016957	highly acetylated
BTN3A3	1.571794229	0.83146591	5.261478155	1.07346E-06	0.000212407	highly acetylated
ZNF331	1.206778527	0.851330349	5.377148659	4.12343E-06	0.000527263	highly acetylated
DUSP9	3.844836142	1.543768128	9.714149685	6.71545E-05	0.000914037	highly acetylated
COL27A	1.350979182	0.740203856	4.647237597	0.000500162	0.008073522	highly acetylated
FLJ2140	2.810361891	0.35582606	2.232065278	0.006846603	0.013559516	highly acetylated
SPRN	1.475243838	0.745552949	4.666310058	0.000395833	0.005393127	highly acetylated
WTIP	1.159455475	0.903809841	5.645693039	0.00114012	0.013185209	highly acetylated
TMEM1	1.615107599	1.380501763	8.613659771	2.5158E-05	0.000415808	highly acetylated
CGN	2.295714925	0.445058963	2.774251596	6.64095E-07	5.86817E-06	highly acetylated
SNORD8	1	1	6.225427727	0.001300388	0.015128162	highly acetylated
ZGLP1	1.441148606	0.771576685	4.789375095	5.93824E-07	0.00203766	highly acetylated
SEMA4A	2.343128008	0.440479043	2.733753203	6.14206E-06	0.000120453	highly acetylated
CTF1	1.384258319	0.768267436	4.739390428	0.00028586	0.005281896	highly acetylated
FGFRL1	9.238936933	0.341538983	2.106697806	0.001212793	0.005663353	highly acetylated
STAC3	1.286134695	0.829975428	5.112125651	0.000480443	0.007396914	highly acetylated
TRIP11	2.123821444	1.184523644	7.29393382	4.28963E-06	0.000157956	highly acetylated
FANCE	5.315717912	0.609145327	3.742825036	0.000150963	0.002377274	highly acetylated
UTP14A	15.2262583	0.363004961	2.229772156	2.59118E-05	0.000111574	highly acetylated
CEP135	3.194919401	0.552275726	3.392252036	6.99402E-06	0.00036105	highly acetylated
TNK2	1.637405609	0.731413503	4.487308599	2.3843E-05	0.000264829	highly acetylated
NES	1.315643084	8.869930016	54.24831652	6.28352E-06	0.000273186	highly acetylated
CALD1	7.619741656	2.662082146	16.21117826	7.14056E-07	8.00953E-05	highly acetylated
UNCX	1.210351899	1.905597165	11.56037988	0.000113585	0.002686107	highly acetylated
RNU6AT	2.752457443	0.363311703	2.188367982	0.041267781	0.038006734	highly acetylated
NPDC1	21.84486811	0.613309208	3.69047062	2.47371E-05	0.001279831	highly acetylated
SIX5	2.536675809	0.611666947	3.676527008	0.000211069	0.004493547	highly acetylated
SLMAP	4.60804249	0.376944875	2.258957638	4.04729E-05	0.000231954	highly acetylated
NXPH4	2.460017478	0.675364546	4.03827954	1.48343E-05	0.000560625	highly acetylated
LOC155(	1.622719562	0.678166493	4.049714822	0.000145121	0.001772945	highly acetylated
LOC728	1.369020036	0.730449499	4.354388331	0.000129167	0.003999357	highly acetylated
FBLL1	1	1	5.950186827	4.24928E-05	0.002674263	highly acetylated
RILP	7.352034143	0.440347711	2.615104447	0.010475646	0.025169584	highly acetylated
BCAM	4.146574306	0.510344889	3.019921869	0.000310345	0.004190449	highly acetylated
ZBTB47	1.315765914	0.969135568	5.697741521	4.99909E-07	0.000269454	highly acetylated
MATN4	1.281711683	0.780206667	4.568580224	0.001047836	0.012030847	highly acetylated
WHAMM	1.474543348	0.695816771	4.072183573	0.000519311	0.007660429	highly acetylated
SNORA4	2.345531449	0.42634261	2.494581224	0.028238545	0.035738018	highly acetylated
BPTF	2.718463461	3.012854339	17.5456609	3.86536E-08	3.9801E-05	highly acetylated
TNFRSF	1.090523046	0.916991166	5.339287523	0.002903129	0.022002944	highly acetylated
RBM41	5.372423856	1.189950721	6.90494028	8.4822E-08	5.03646E-05	highly acetylated
UPF2	3.690703532	1.749941715	10.14742168	2.69203E-05	0.00068732	highly acetylated
ACADS	11.89472425	0.363781846	2.108827535	0.001718957	0.00663029	highly acetylated
UTF1	1.113615991	1.070022301	6.198193126	0.00033954	0.003844001	highly acetylated
ENOSF1	4.04996859	0.352731461	2.041843647	0.00016033	0.000992617	highly acetylated
LSR	4.849562179	0.51783493	2.992162207	0.000278659	0.001646125	highly acetylated
RBM25	19.94800013	8.388133185	48.38829549	2.32707E-05	0.001535268	highly acetylated
C10orf47	1.591736743	0.690920385	3.981952921	0.00034914	0.00441332	highly acetylated
MTMR1	3.116397893	0.557746272	3.211202106	2.84376E-06	0.014405701	highly acetylated
CHD3	2.872357733	3.027624273	17.39901327	1.42574E-06	0.000632155	highly acetylated
ZNF37BI	1.836464006	1.745551716	10.01888632	1.16927E-07	0.005092308	highly acetylated
SMARC/	1.957124485	0.666989638	3.816473887	5.1294E-06	0.00097351	highly acetylated

GMFG	1.559536667	0.641216087	3.631650312	0.012122154	0.036993793	highly acetylated
IZUMO4	1.747983993	0.572087619	3.226946091	0.007531212	0.026590624	highly acetylated
SLC2A11	3.061634329	0.540995009	3.050805173	0.001753838	0.009765816	highly acetylated
LCAT	1.473089787	0.734414846	4.116486105	0.00292771	0.018390399	highly acetylated
TCOF1	15.06570781	6.411385568	35.87180109	6.0974E-07	0.000339024	highly acetylated
DDX12P	2.818548324	0.42190472	2.350442323	4.86161E-05	0.000580754	highly acetylated
ARHGAI	2.561330867	0.390422031	2.173049669	0.000267226	0.002607581	highly acetylated
LOC2853	1.240166208	0.806343532	4.485379344	0.01542762	0.050225274	highly acetylated
C1orf54	32.29459349	0.656645492	3.643193631	1.14825E-05	0.002468345	highly acetylated
PELI3	1.94564163	0.632420455	3.50618277	0.000390913	0.003064373	highly acetylated
SGSM2	2.593174519	0.747046637	4.134660002	0.000126572	0.002203666	highly acetylated
TAOK3	4.300714618	0.598258726	3.308252696	1.5962E-05	0.000154239	highly acetylated
C2CD4C	1.251079804	0.827668404	4.570202134	7.50109E-05	0.002825427	highly acetylated
HOXC8	1.562446037	3.632289542	20.02705236	3.73454E-06	0.00056082	highly acetylated
FBXL14	1.381148602	0.724035052	3.989569674	5.1323E-06	0.000357837	highly acetylated
GOLGA2	1.360814518	0.807462952	4.440968747	0.000100451	0.001806695	highly acetylated
LOC1001	1.7194177	3.621799221	19.91161545	4.73404E-08	0.025612985	highly acetylated
CSPG5	1.846426537	0.54158667	2.974233778	0.00033601	0.004928015	highly acetylated
PHF21B	1.978645618	0.866658096	4.758475767	2.23693E-05	0.000412788	highly acetylated
HNRNPU	1.691215248	5.506623553	30.22512245	1.88049E-09	0.001588792	highly acetylated
ODF3L2	1.907016899	0.596848864	3.274512669	0.007965938	0.025651547	highly acetylated
LOC1002	1.743499449	0.573559114	3.141381257	0.000117629	0.001705874	highly acetylated
KHDC1	2.014689349	0.496354438	2.714028507	0.000567399	0.003451542	highly acetylated
MRPL42	2.606572382	0.38364559	2.083765396	0.00077674	0.000437394	highly acetylated
CLDN15	2.467395817	0.445321859	2.415697095	0.003374174	0.00835573	highly acetylated
HMGN5	13.20415979	21.13913456	114.660302	0.00011796	0.004193961	highly acetylated
TMEM13	2.028076506	0.653548424	3.540446655	0.000267503	0.003191606	highly acetylated
RAB35	8.897063233	0.386816902	2.090826	9.19056E-08	0.028679913	highly acetylated
ATF5	2.502352828	0.928204379	5.014044476	1.12332E-05	0.000563858	highly acetylated
SHF	2.011731835	0.497084145	2.682676105	0.000277767	0.003021959	highly acetylated
GLIS2	1.654615834	0.638831769	3.4428154	0.000280425	0.004126387	highly acetylated
VTRNA1	2.650024742	0.377354967	2.031237685	0.035281154	0.027325675	highly acetylated
SLC12A9	1.679402831	1.145160499	6.146554333	4.67402E-08	0.001790039	highly acetylated
FAM98B	2.758559706	2.761380587	14.79709832	7.08304E-05	0.004414198	highly acetylated
RGS12	1.506450157	0.776083384	4.148666042	0.000306741	0.006280014	highly acetylated
REV1	2.954312523	0.799047294	4.253686392	8.38777E-05	0.003194805	highly acetylated
PRRC2C	8.800641816	6.767416969	35.99979421	4.41809E-05	0.003275869	highly acetylated
CORO6	3.782750841	0.709178613	3.765720179	1.59252E-05	0.0048199	highly acetylated
NT5M	2.297721536	0.640715951	3.392071535	0.001246902	0.006666399	highly acetylated
TAF1C	3.227006632	0.57156183	3.010540762	3.81569E-05	0.000276052	highly acetylated
TARBP1	2.974569865	0.44138261	2.320346752	0.000935488	0.003599525	highly acetylated
HES5	1.250885746	0.867279535	4.549030417	0.002708734	0.019114748	highly acetylated
CGNL1	1.257101349	0.936824515	4.91347554	4.35971E-05	0.002205094	highly acetylated
NKAPP1	2.11852727	0.472026022	2.460773864	2.23371E-05	0.000962798	highly acetylated
DSP	5.366437157	1.758092124	9.159667672	1.38908E-06	6.14067E-05	highly acetylated
PIF1	2.343934829	0.461404384	2.394247508	0.00395227	0.013691568	highly acetylated
GPR161	3.338692709	0.500717331	2.597746801	0.004172375	0.012467136	highly acetylated
SEC31B	1.842088127	1.044313139	5.415455207	0.000168565	0.001445543	highly acetylated
KLF16	4.913174905	0.942781057	4.877044218	7.88658E-07	3.48208E-05	highly acetylated
NAT8L	1.467324364	1.036929393	5.354132944	0.000160907	0.001675147	highly acetylated
RPS15AI	1	1	5.160206083	0.000188671	0.005665745	highly acetylated
SBK1	1.756007127	0.704400987	3.623076322	0.000182518	0.003426254	highly acetylated
UPF3B	26.5386879	7.468342512	38.29023489	1.19223E-05	0.001339821	highly acetylated
TAGLN	1.489093905	0.719354444	3.687530505	0.000120794	0.0021707	highly acetylated
CAMKK	2.485912037	0.724399195	3.70757644	3.50458E-05	0.001041264	highly acetylated

ZNF777	1.122317364	1.459101423	7.441868988	5.59138E-05	0.001370224	highly acetylated
FOXRED	5.8483165	0.525583473	2.676687291	1.77019E-06	0.000667851	highly acetylated
PLEKHO	10.64083667	0.679256571	3.437944696	1.53628E-05	0.000940388	highly acetylated
SNORA7	1.072892734	0.932059626	4.711996765	0.000614497	0.009635243	highly acetylated
C19orf29	1.567127589	0.772889578	3.903171912	0.000224199	0.00318654	highly acetylated
BTN2A3	1.942345603	0.514841436	2.59817297	0.000203646	0.002607686	highly acetylated
RRN3P3	1.284857689	0.778296312	3.922886046	7.97005E-05	0.003068663	highly acetylated
DMPK	1.697031933	0.607411456	3.057737385	0.000586192	0.006191744	highly acetylated
NKX3-2	1.231660173	0.811912264	4.087035522	0.004201679	0.024636141	highly acetylated
C9orf169	1.503344928	0.66518334	3.343873466	0.000496223	0.005678221	highly acetylated
MIA3	6.472390436	1.038295658	5.212249914	6.0743E-07	0.000418085	highly acetylated
SLC5A5	2.61453347	0.418041748	2.098396993	0.000646233	0.002390388	highly acetylated
MPHOSI	4.008079223	6.239521763	31.31196324	5.12799E-06	0.000912552	highly acetylated
GRIK5	1.018734864	1.072677317	5.379752867	2.67291E-05	0.001760337	highly acetylated
RRP12	6.408392292	0.576903088	2.887887437	2.98016E-06	0.000613029	highly acetylated
SLC39A1	11.16443162	0.411511917	2.055093751	0.000989449	0.001390037	moderately acetylated
SLC7A5I	1.956558516	1.187841225	5.928435773	1.96281E-05	0.001471417	moderately acetylated
C3orf78	82.80198564	0.480395853	2.393556864	7.4422E-05	0.005485392	moderately acetylated
TRIM45	2.412664663	0.775065439	3.856108509	7.44516E-06	0.001438818	moderately acetylated
KRBA1	3.048637098	0.568861384	2.828259545	3.04982E-05	0.00196267	moderately acetylated
CORT	1.219061157	0.82030339	4.078321676	0.008160183	0.035258953	moderately acetylated
ITIH4	1.233733508	0.865277494	4.297911961	0.000470006	0.006951382	moderately acetylated
NOVA2	1.234565574	0.810001527	4.006370917	2.9186E-05	0.001191858	moderately acetylated
KCNJ4	1.31705202	0.916191012	4.527871865	1.69379E-05	0.00017294	moderately acetylated
SOCS7	1.967013387	1.026825369	5.060437305	4.556E-06	0.000134694	moderately acetylated
GOLGA8	2.542450008	3.863270938	19.02124986	4.9728E-06	0.000184485	moderately acetylated
DOCK6	2.19283632	0.517728108	2.537838055	6.12783E-06	0.000252528	moderately acetylated
PDZD4	1.412746463	3.325397328	16.2758875	4.04924E-06	0.001106088	moderately acetylated
C3orf62	1.194086303	1.719335578	8.396952907	2.23035E-06	0.000305687	moderately acetylated
CDK11B	2.618387358	1.072877636	5.212326374	4.48877E-06	0.003692492	moderately acetylated
FAM155I	1.711585237	1.139117882	5.515208098	0.000191516	0.001811926	moderately acetylated
CHD2	3.312934421	3.377172397	16.34520981	2.25873E-06	0.000150995	moderately acetylated
AZI1	3.042620604	1.638107324	7.923526396	3.73105E-05	0.00128985	moderately acetylated
SRM	40.52437204	1.537265388	7.410351944	7.59764E-06	0.000732809	moderately acetylated
MSRB2	10.39878221	0.681837577	3.280746855	4.22609E-07	5.94468E-06	moderately acetylated
HSPB3	1.023243856	0.977284148	4.693251092	0.000273205	0.006713191	moderately acetylated
MTA1	5.331775859	2.637496236	12.63396053	1.53489E-05	0.001115234	moderately acetylated
WIPF2	3.909784001	0.664871493	3.16686221	9.53175E-05	0.002329623	moderately acetylated
NT5DC2	15.14731855	0.473670105	2.256095778	0.000468587	0.002094806	moderately acetylated
COL7A1	1.071944865	1.067677296	5.079020519	9.20604E-05	0.001354289	moderately acetylated
NKX2-2	1.425936555	0.925512703	4.390464574	0.000893933	0.004788793	moderately acetylated
PCM1	3.823335632	2.223052629	10.53746293	3.45763E-08	7.21217E-05	moderately acetylated
DST	1.502364811	1.591567308	7.541887828	8.0886E-07	0.000287823	moderately acetylated
SCARF2	1.454842444	1.514726825	7.144099273	7.73005E-06	0.000941583	moderately acetylated
PRR22	1.729392975	0.624279367	2.940326566	0.001851436	0.010678608	moderately acetylated
SCGB1B	1.214515357	0.823373698	3.873701403	0.005084588	0.027031851	moderately acetylated
PFN4	2.156176898	0.463783839	2.18154895	0.008497227	0.018044696	moderately acetylated
BAZ2B	1.749814742	1.316451344	6.16713592	5.68014E-05	0.002738332	moderately acetylated
PIGZ	1.292643445	0.773608533	3.618698982	0.000960578	0.010887058	moderately acetylated
LRRFIP1	6.137101459	4.09962364	19.16044054	7.23041E-09	6.71621E-06	moderately acetylated
LOC2864	2.883041584	0.436299015	2.035595226	0.000408391	0.002830154	moderately acetylated
CRTC2	9.830045171	0.621614172	2.896985439	0.000483945	0.002352644	moderately acetylated
SRRM2	5.633819714	3.192938131	14.87709189	8.44011E-07	0.000111519	moderately acetylated
NOVA1	1.557467835	0.642067834	2.991145366	0.000427417	0.005896219	moderately acetylated
C17orf96	2.128643972	0.945218044	4.401461128	4.13755E-06	0.019283157	moderately acetylated

NCKAP5	2.065313947	0.676184572	3.145420804	0.000135298	0.003113126	moderately acetylated
C10orf11	1.593574272	0.701831856	3.260880499	1.76632E-05	0.000328955	moderately acetylated
TMEM54	19.23917049	0.644663359	2.994742092	0.004213647	0.016629623	moderately acetylated
MUC1	1.124997134	0.888891153	4.124927186	2.64836E-05	0.001821767	moderately acetylated
IGFBP4	2.758303842	1.09791001	5.089419495	3.17181E-06	6.4116E-05	moderately acetylated
TMEM86	1.267488472	0.938784033	4.34850054	0.001547246	0.009926915	moderately acetylated
WBSCR1	3.735001969	0.476951421	2.205587324	0.000447208	0.001335722	moderately acetylated
DEXI	6.296140219	0.980121478	4.526155715	1.74761E-07	0.016035661	moderately acetylated
LOC2844	1.513381404	0.741401338	3.417457238	2.97023E-05	0.001515971	moderately acetylated
DFNB59	2.019067192	0.495278218	2.282634575	6.06536E-05	0.000643002	moderately acetylated
RAB32	1.482150351	2.318045195	10.6770451	4.64555E-05	0.001275473	moderately acetylated
ZHX1-C8	2.168453738	0.461158097	2.12080077	0.001360706	0.005623672	moderately acetylated
SPEN	2.970700532	3.479302493	15.9443768	2.76879E-06	0.000900404	moderately acetylated
C17orf59	5.488108013	0.646134464	2.959221961	0.000486468	0.005449465	moderately acetylated
ST3GAL	1.930816266	0.781542229	3.575875447	4.89702E-06	1.49783E-05	moderately acetylated
BAZ1B	5.589799281	6.790946361	31.0669623	8.26186E-08	0.000131312	moderately acetylated
TAF1	2.361699997	0.696919229	3.178042067	4.47805E-05	0.001711263	moderately acetylated
CCDC8	4.328550324	1.69874084	7.730144953	7.50583E-07	0.008519439	moderately acetylated
KIF26A	1.314485184	0.856482687	3.894812933	2.53562E-06	0.000636512	moderately acetylated
LOC2831	1.489465492	0.685406287	3.114954956	1.14182E-06	1.62245E-05	moderately acetylated
ABHD12	1.676716529	0.596403735	2.710211532	0.001404075	0.009950976	moderately acetylated
ANKRD3	2.105077504	1.094205636	4.969207341	4.38187E-06	0.004673778	moderately acetylated
DTX2	3.87445505	0.510678492	2.313691425	0.000663324	0.005693033	moderately acetylated
YLPM1	2.768202267	1.29644052	5.867290384	4.19635E-07	0.004555763	moderately acetylated
LOC3405	1.799852152	0.555601191	2.512933332	0.000886382	0.007354423	moderately acetylated
INPPL1	5.090526838	0.693378988	3.13589254	0.000372842	0.004351029	moderately acetylated
EGR1	1.570507577	0.63673682	2.877376015	7.8564E-06	0.000757314	moderately acetylated
GPATCH	28.02603837	2.41164645	10.89249381	5.03698E-08	0.000159695	moderately acetylated
SLC25A2	7.942172227	0.579927083	2.612151648	0.000141998	0.001863543	moderately acetylated
TMEM44	2.06810099	0.52607003	2.367175028	0.00035944	0.001820717	moderately acetylated
ATP1B2	2.387227289	0.458735781	2.063010981	0.00118194	0.003247578	moderately acetylated
C19orf81	10.40865207	1.093252333	4.913189926	2.81483E-07	0.029933578	moderately acetylated
AP3B2	1.315281671	0.937977418	4.213397238	0.001673323	0.016389337	moderately acetylated
TMEM91	3.285330243	0.468114912	2.102581318	0.005637156	0.005053132	moderately acetylated
THAP4	14.12716828	0.53197917	2.384967093	9.16922E-06	0.000129921	moderately acetylated
SNORA2	1.428903478	0.699837334	3.131235246	0.010546584	0.031185459	moderately acetylated
SYT5	2.052916687	0.487111828	2.163937059	0.004767478	0.014617177	moderately acetylated
TMEM18	2.298397678	0.483431461	2.145748851	5.09323E-05	0.000223558	moderately acetylated
RAD50	3.890197536	1.719923364	7.626914628	7.9512E-07	3.5931E-05	moderately acetylated
NASP	68.59143133	1.533553305	6.799675714	1.79558E-07	5.14205E-05	moderately acetylated
EXT1	4.968578805	0.550114283	2.434501822	0.000277442	0.002235044	moderately acetylated
ZNF234	1.565641812	0.638715696	2.822707327	0.000107107	0.00217482	moderately acetylated
TSPAN1	1.152153575	0.867939849	3.834739323	0.000263493	0.006120936	moderately acetylated
LIME1	1.519633554	1.605642016	7.093880303	2.70735E-05	0.001047023	moderately acetylated
TRAF3IP	1.902783628	2.829821699	12.49820526	1.18869E-05	0.000794216	moderately acetylated
ORAI3	2.077140127	0.554594469	2.447983956	0.000390704	0.001301155	moderately acetylated
CLASRP	4.704179519	0.946040249	4.173070041	5.3357E-07	0.012823486	moderately acetylated
MST1	1.684377598	0.593691107	2.61450047	0.000663028	0.004963357	moderately acetylated
LOC2021	2.603777996	1.06613926	4.689786436	0.000139575	0.003565774	moderately acetylated
SLC38A7	2.175727669	0.459616346	2.020858456	0.000277858	0.002439633	moderately acetylated
LOC1501	1.621783227	0.675656041	2.963343457	2.48565E-05	0.000178163	moderately acetylated
ZNF746	2.097343732	0.692773474	3.026635794	0.000599647	0.005467861	moderately acetylated
NAPRT1	1.356689502	0.833485653	3.641297367	0.000392502	0.002794273	moderately acetylated
CHD7	2.510782704	2.4238671	10.58630853	2.46362E-07	0.002335691	moderately acetylated
STAT2	2.712441797	0.738271654	3.222924129	0.000299471	0.002895213	moderately acetylated

THAP11	7.734724544	0.915404734	3.992628115	3.89752E-06	0.000724325	moderately acetylated
RING1	2.231903548	0.747014572	3.256487409	6.09429E-06	0.000179816	moderately acetylated
ZCCHC3	5.248105071	0.915177392	3.987933117	4.51096E-06	0.000367228	moderately acetylated
GDPD3	1.846221569	0.541646797	2.358652171	0.000143411	0.00238898	moderately acetylated
ADAM11	2.053601269	0.739243958	3.206561693	2.86136E-06	6.52912E-05	moderately acetylated
TATDN2	2.195970554	0.867440255	3.753690697	3.61031E-05	0.001707568	moderately acetylated
OR7E12I	1.3825843	0.786477276	3.400025327	0.000130046	0.00143497	moderately acetylated
PLK5	1.873681602	0.533708608	2.306951298	0.012694119	0.026216426	moderately acetylated
RBPMS	1.815973684	1.105048293	4.765596292	0.000133757	0.001285343	moderately acetylated
CYB561I	1.69077289	0.641098193	2.75496231	1.93332E-06	0.000272727	moderately acetylated
LTC4S	1.864114239	0.536447809	2.304146155	0.000375785	0.001902478	moderately acetylated
LAMB1	5.224969556	0.668983616	2.873069918	7.92079E-05	0.001921131	moderately acetylated
MEGF6	1.355305465	0.737841045	3.167585968	2.1523E-05	0.001352626	moderately acetylated
SFSWAP	8.297579469	1.39835476	6.000606215	6.25804E-08	0.018003866	moderately acetylated
DACT3	1.309740537	0.763510002	3.273555201	0.000103545	0.003364418	moderately acetylated
HIRIP3	21.92421122	2.914009105	12.48588632	1.94779E-06	9.39785E-05	moderately acetylated
CCDC88	1.720349156	1.667287259	7.14385575	7.04419E-05	0.004228696	moderately acetylated
CABP1	1.245663161	0.80278524	3.435927624	0.000119408	0.002745686	moderately acetylated
INTU	2.552922421	0.488643003	2.088677755	0.002058427	0.00832809	moderately acetylated
EHBP1L	1.105297003	2.628191584	11.23343154	1.01182E-05	0.000428753	moderately acetylated
ZNF721	3.236650821	0.834373185	3.563875817	0.000441281	0.004733269	moderately acetylated
SLK	2.843331495	3.74131311	15.96815895	4.42252E-07	0.000355478	moderately acetylated
LOC1005	1.136860494	0.879615402	3.75319077	0.002934932	0.020944193	moderately acetylated
CCDC16	1.774315644	0.563597578	2.398325216	1.74277E-06	0.000217604	moderately acetylated
PLEC	1.446817345	0.96417978	4.078856143	4.71346E-07	0.000156824	moderately acetylated
ZCCHC6	3.350959081	1.188610173	5.027416496	2.17964E-08	0.009085289	moderately acetylated
C2orf82	1.039189642	1.191658672	5.035872799	0.000563711	0.004152214	moderately acetylated
GRK5	3.127045163	0.850428999	3.576524059	3.41359E-05	0.002901771	moderately acetylated
LTB4R2	1.117058214	0.958812538	4.026593706	1.76826E-05	0.00033034	moderately acetylated
NGEF	3.618311523	0.492528661	2.067530788	0.004193048	0.00998721	moderately acetylated
MAP4K4	3.422442895	2.843383843	11.91580941	5.58262E-09	0.013323906	moderately acetylated
PPP1R13	1.922577034	0.749145122	3.138932655	3.58958E-06	7.95041E-05	moderately acetylated
LRRC73	1.117894687	0.894538647	3.746824141	0.000307181	0.006647567	moderately acetylated
LOC7289	2.066773472	0.483845963	2.026118696	0.009238683	0.012009986	moderately acetylated
LOC3897	1.908497016	0.523972525	2.19337517	0.022945325	0.033073547	moderately acetylated
C8orf42	1.148370913	0.897671111	3.753413365	0.001513582	0.01477801	moderately acetylated
VAC14	5.789672262	0.695289424	2.90513637	0.00101277	0.004239761	moderately acetylated
AHRR	2.342760483	0.549087476	2.287253334	0.001202868	0.007350968	moderately acetylated
FZD8	1.23196613	0.868187494	3.609426804	5.65518E-05	0.000960978	moderately acetylated
AZU1	1.122428243	0.89092555	3.700651906	0.005592229	0.029211969	moderately acetylated
DNLZ	4.151029922	3.086872951	12.81729871	2.56727E-09	0.001358607	moderately acetylated
PRPF4B	6.451451938	2.04279581	8.468492389	2.24711E-08	0.008984762	moderately acetylated
FAM193I	2.955901932	5.300668298	21.97376409	1.34047E-07	5.29805E-05	moderately acetylated
AKAP12	3.490314015	5.132852927	21.27766953	4.34135E-07	3.54699E-05	moderately acetylated
ANKRD1	1.601552113	3.22850693	13.37403017	2.83447E-06	0.00012913	moderately acetylated
GPR20	1.687932934	0.678501408	2.808974737	0.000316686	0.001080663	moderately acetylated
TSPYL4	3.667067105	0.6100503	2.519475099	0.000521771	0.001677303	moderately acetylated
BHLHE4	1.28841448	1.198933968	4.949798578	1.08751E-05	0.000811025	moderately acetylated
TBX6	1.449954689	0.689676724	2.838686245	0.000528901	0.00621754	moderately acetylated
SEMA3B	1.653370171	0.604825234	2.488766899	0.001132872	0.008550429	moderately acetylated
EPB41L3	4.061427498	1.270388741	5.223244434	5.2889E-07	0.035694323	moderately acetylated
FAM83H	1.121993672	0.993853691	4.083129592	7.45321E-07	3.09304E-05	moderately acetylated
IDUA	1.206773606	0.828655843	3.401786567	0.000776665	0.009708002	moderately acetylated
MYH10	8.663372519	2.703989238	11.09917339	9.22247E-07	5.67695E-05	moderately acetylated
CASP8A	5.812973158	1.025672219	4.206240879	2.24126E-06	0.001114706	moderately acetylated

RBM28	16.11353143	2.504426877	10.26711777	3.39291E-08	3.8583E-06	moderately acetylated
PRRT4	1.386990844	1.40391173	5.75437804	0.000262897	0.007433971	moderately acetylated
SUGP2	7.898936915	2.855978427	11.69456889	1.02911E-06	7.77198E-05	moderately acetylated
SMPD3	1.103646892	0.906086908	3.710051729	9.81409E-05	0.00377301	moderately acetylated
LRRIQ1	1.394325867	1.158872313	4.7427076	1.31408E-07	9.85852E-05	moderately acetylated
TP53BP1	2.618244712	0.812754007	3.326069605	8.09197E-06	0.00036379	moderately acetylated
MIR210H	2.167251656	0.504784142	2.06556313	0.01923379	0.030462487	moderately acetylated
FBXL19-	1.398467671	2.598410872	10.61112529	1.05132E-06	8.50539E-05	moderately acetylated
TLCD2	1.404915993	2.793555779	11.39468425	1.09307E-06	0.001717646	moderately acetylated
HIST2H2	1.475585764	0.677696969	2.762661785	0.005026973	0.020340914	moderately acetylated
GRK4	2.155751095	0.72111114	2.939230448	0.00083717	0.002911871	moderately acetylated
DEFB1	1.214382873	0.823463524	3.356280693	0.034733766	0.07712863	moderately acetylated
MLK7-A	1.191107323	0.839554909	3.418178383	0.000339404	0.00661391	moderately acetylated
ZIC2	3.408292067	1.674394098	6.807216982	2.8405E-05	0.001044605	moderately acetylated
DEM1	1.580536844	0.632696418	2.565844255	1.51687E-05	0.000993867	moderately acetylated
MAGI3	2.192460728	1.311613846	5.312290982	7.56488E-05	0.001233623	moderately acetylated
HOXD11	6.572590541	0.975730323	3.947124305	1.15629E-05	0.000506634	moderately acetylated
TRIOBP	1.70035003	0.688351012	2.783439336	0.000208592	0.002849221	moderately acetylated
LINGO1	1.130779267	1.312662683	5.30507419	2.59866E-05	0.000796501	moderately acetylated
RIIAD1	1.13296142	1.097652571	4.433359602	0.000790201	0.00486113	moderately acetylated
TAF15	9.130523345	12.78438378	51.42801173	8.94262E-08	0.00012788	moderately acetylated
SORBS3	8.201823626	0.615216391	2.473281875	2.45455E-05	0.020784796	moderately acetylated
TRIM54	1.065517544	0.93851106	3.765380321	0.000130629	0.004353684	moderately acetylated
AMH	1.130054469	1.835291646	7.361472251	3.03699E-05	0.001966771	moderately acetylated
LOC729(	1.751327087	0.570995565	2.287808174	0.01526323	0.022635409	moderately acetylated
DUSP7	2.356830774	0.606568291	2.427444286	6.26555E-05	0.004130945	moderately acetylated
LYPD1	1.627398026	0.614477825	2.454569726	6.51711E-05	0.001774759	moderately acetylated
MIR125E	1.501008085	0.66621893	2.650638346	0.042168183	0.065507787	moderately acetylated
PMEPA1	1.625298442	1.234646097	4.911025001	0.000381259	0.003645183	moderately acetylated
CBX6	6.199793303	2.775050649	11.02433837	3.78672E-06	0.000234638	moderately acetylated
ACAP1	1.977655885	0.941032122	3.737255508	0.000533219	0.008214567	moderately acetylated
EMX1	2.270151272	0.834820621	3.311933236	2.3809E-05	0.000724552	moderately acetylated
TRIM41	2.859826589	1.558951686	6.181434469	6.18437E-05	0.001689212	moderately acetylated
CENPJ	2.849615886	2.487960165	9.855696581	5.68662E-07	4.18698E-05	moderately acetylated
ZNRF3	1.619403362	0.677220421	2.680143138	0.001367154	0.009570944	moderately acetylated
FOXD2	1.369699747	0.847187147	3.34678646	3.74223E-05	0.000588265	moderately acetylated
SNORD1	1.877077642	0.532743014	2.104011254	0.030474653	0.020434246	moderately acetylated
TNIP1	8.054931457	0.675282703	2.665504625	0.00011528	0.003706868	moderately acetylated
SMC6	7.943367332	0.675295257	2.660681099	1.32182E-05	0.000427888	moderately acetylated
PVRIG	1.18990905	0.840400365	3.308056413	5.19661E-05	0.002578925	moderately acetylated
GFOD1	1.902878276	0.569141209	2.217323098	8.8818E-05	0.000859054	moderately acetylated
TCEAL7	1.609039628	0.621488733	2.413257153	0.003875553	0.015019305	moderately acetylated
NANOS3	1.196228864	0.835960433	3.24357476	0.002774956	0.018875731	moderately acetylated
NRG4	2.761684269	0.661961683	2.566707803	0.001951028	0.015121919	moderately acetylated
GRIPAP1	3.630358687	2.938709754	11.38543849	1.67358E-06	0.000131614	moderately acetylated
SAFB2	3.915327194	2.858627111	11.03352674	4.59227E-06	0.000206233	moderately acetylated
PRRT3	1.803332823	0.704789358	2.718177546	0.001129489	0.006833738	moderately acetylated
ODF3L1	1.913758769	0.522531897	2.015090693	0.000502392	0.003546126	moderately acetylated
CCNE1	6.859742567	0.654566939	2.520624145	0.000742091	0.003422345	moderately acetylated
PNMT	1.822590652	0.756444593	2.910990754	0.007609517	0.021835125	moderately acetylated
RPL13P5	1.913376668	0.522636246	2.008560992	0.000281806	0.000424052	moderately acetylated
VPS18	2.470739733	0.751496233	2.883885884	9.23702E-06	0.000901346	moderately acetylated
NEMF	6.876535661	1.275775186	4.895396273	5.44249E-07	0.000107134	moderately acetylated
GALNT6	3.287414162	1.155897917	4.433412856	7.39386E-06	0.001110616	moderately acetylated
SIM2	1.342081192	0.825251659	3.158134457	1.02252E-05	0.000122246	moderately acetylated

PPAPDC	3.570706445	0.555226532	2.122380295	0.001465438	0.009590462	moderately acetylated
ROCK2	1.780170807	3.841768436	14.68350837	1.02691E-06	0.000642941	moderately acetylated
PYY2	3.448241375	4.067849875	15.5352782	2.88288E-05	0.002193891	moderately acetylated
HOXD4	1.338974801	1.181942425	4.501270739	0.005486777	0.03414249	moderately acetylated
SYCP2	1.113170651	0.950283385	3.617194247	1.88096E-06	7.7169E-05	moderately acetylated
MBD6	2.158033456	1.100130123	4.181173956	5.68274E-06	0.003274537	moderately acetylated
CUEDC1	1.331391393	0.895807715	3.398703633	0.0004988	0.002656805	moderately acetylated
GPT	1	1.11201665	4.202263441	3.52421E-07	0.007689684	moderately acetylated
CAPS	1.180386686	0.847180006	3.201219898	0.001255248	0.012769107	moderately acetylated
HEG1	1.714986478	0.610666376	2.305930561	0.001481791	0.009621667	moderately acetylated
SPG7	5.425029768	1.134579896	4.275908975	8.68645E-05	0.001142607	moderately acetylated
ZBED3	1.443050224	0.744562405	2.802541234	0.001061633	0.007517515	moderately acetylated
ZNF137P	1.058360493	0.987017577	3.711140804	0.000406104	0.006879626	moderately acetylated
FAM207A	14.17788963	2.077521222	7.793048501	5.22729E-07	4.38516E-05	moderately acetylated
TUSC1	2.318292265	1.413329774	5.300542431	0.000163136	0.006297795	moderately acetylated
CC2D1A	4.075435449	0.914156138	3.421878113	0.000510119	0.004567577	moderately acetylated
CTDP1	1.406324134	0.752111551	2.814590703	0.000510539	0.005054563	moderately acetylated
MNS1	1.246175638	1.039102225	3.881780887	0.008804684	0.041718369	moderately acetylated
ASIP	1.275404003	0.784065282	2.92897825	0.001451584	0.007337669	moderately acetylated
GFI1	1.835526827	0.605105543	2.25563966	0.001110729	0.00697838	moderately acetylated
C17orf98	1	1.186628448	4.421915614	0.000411722	0.003380415	moderately acetylated
SEPT9	5.351701252	0.832708001	3.100821519	1.46564E-06	0.002781242	moderately acetylated
NLGN2	1.630615877	1.594744949	5.938448446	5.39827E-06	0.001913484	moderately acetylated
PNMA6C	1.28732721	0.814967563	3.033973179	0.003785637	0.020849153	moderately acetylated
GRIN2D	1.138114221	1.557728286	5.781618957	0.000226996	0.005873537	moderately acetylated
LOC1001	1.133638185	0.882115664	3.271826369	0.001495487	0.014609891	moderately acetylated
VGF	1.016558661	1.024412618	3.78880627	5.59346E-06	0.000182432	moderately acetylated
ZNF579	1.411423164	4.452739906	16.45493367	7.32554E-06	0.001134169	moderately acetylated
NAIF1	1.689182787	0.629715609	2.326337347	0.001509635	0.010050539	moderately acetylated
CDH24	1.470111828	1.100628395	4.06333991	0.000440078	0.005660879	moderately acetylated
ANKDD1	1.101231605	0.908074192	3.347298236	0.000119624	0.004137099	moderately acetylated
TMPRSS	1.164717034	0.858577638	3.160109334	0.001773224	0.015507347	moderately acetylated
KCTD15	3.498469906	0.642938509	2.365318196	4.90886E-05	0.001289955	moderately acetylated
GIPC1	16.84838359	1.306669335	4.800240445	1.84603E-05	0.001407112	moderately acetylated
KTN1	11.83756409	4.016819547	14.75320888	9.721E-07	7.61027E-05	moderately acetylated
CHTF18	5.700035487	2.144808352	7.872618352	0.000346007	0.006291917	moderately acetylated
UTP14C	2.423998697	2.785973669	10.21762169	1.62032E-07	0.013137811	moderately acetylated
CEBPD	1.08400253	1.638911533	6.009976444	0.000504036	0.01118535	moderately acetylated
CELF5	1.796171813	0.579660259	2.125023188	0.000485242	0.004667966	moderately acetylated
CHKA	3.832217671	0.829922196	3.040481521	5.9623E-05	0.000903721	moderately acetylated
LOC7299	1.944613769	0.574111703	2.103252994	0.022934708	0.032086094	moderately acetylated
TUBB4A	11.9519718	0.691307678	2.530768543	5.52935E-05	0.001796434	moderately acetylated
SUV420H	3.433546294	1.881642051	6.885252794	7.0758E-05	0.001311821	moderately acetylated
ESF1	7.12766061	3.222801251	11.78956792	1.80445E-05	0.002553317	moderately acetylated
C6orf141	1.157523195	0.863913574	3.160313686	0.000534779	0.008428401	moderately acetylated
ZMYND	1.306503254	0.765401844	2.799479928	0.000518621	0.007050941	moderately acetylated
PRKCD	7.178905716	0.54891622	2.00692221	0.001587297	0.007321543	moderately acetylated
CEP152	3.015573824	2.891786911	10.55734264	6.68433E-06	0.000265386	moderately acetylated
MGC129	1.457721667	1.132388774	4.129438372	0.000811489	0.008803228	moderately acetylated
CATSPE	1.288744205	1.421877118	5.182113969	0.000100578	0.004481116	moderately acetylated
SFXN5	2.366009181	0.57575348	2.097990593	0.000671423	0.001547344	moderately acetylated
H1FX-AS	1.580641478	0.632654535	2.299814603	0.004937259	0.017227528	moderately acetylated
ZNF385A	4.466228581	0.804184988	2.922857751	9.97977E-05	0.000765833	moderately acetylated
FGF9	1.568802702	0.836516971	3.036304285	3.01544E-05	0.000265583	moderately acetylated
HSPBP1	25.20904923	0.916507637	3.320608078	0.000155005	0.004514843	moderately acetylated

KIAA022	1.513128393	0.70058837	2.537697767	4.5045E-06	0.000509678	moderately acetylated
FAM211	2.406993771	0.963767279	3.488183777	0.000286543	0.004221036	moderately acetylated
CACNA2	1.791905699	0.558065082	2.019788985	0.001229748	0.007223722	moderately acetylated
ANKRD2	1.124412517	1.301074287	4.707174544	1.92108E-05	0.000805835	moderately acetylated
WDTC1	3.303260441	0.77905601	2.808190268	6.34542E-05	0.000813299	moderately acetylated
C9orf40	4.499825875	1.77055569	6.379016626	2.56438E-06	0.000127743	moderately acetylated
ZFC3H1	3.386030114	0.744395443	2.676836884	0.002042876	0.013870468	moderately acetylated
LIX1L	4.330566757	1.088928119	3.912162342	5.81046E-05	0.000705977	moderately acetylated
SEMA6B	1.201044307	0.856019186	3.07021747	0.000539709	0.007809886	moderately acetylated
ASPM	3.663533764	0.732414896	2.622256986	2.83263E-06	0.004388856	moderately acetylated
MYADM	6.967411291	0.567704534	2.031441294	0.004410292	0.012700199	moderately acetylated
TRIM28	31.31043979	1.380345469	4.933212787	2.90202E-05	0.002463214	moderately acetylated
CSPP1	2.942126313	2.459209805	8.786309725	3.79629E-06	0.00014541	moderately acetylated
ALDH16	2.617204781	1.264048374	4.513566449	1.34466E-05	0.001704798	moderately acetylated
LOC729	1.281825598	0.78013733	2.784733046	0.000783352	0.008723482	moderately acetylated
CDK10	16.4541451	0.874064005	3.118754948	1.52232E-05	0.000750053	moderately acetylated
EZH1	5.17190009	0.582029868	2.071529784	1.7158E-06	0.023221216	moderately acetylated
NKD2	6.786385929	0.901540489	3.207877984	2.663E-06	3.00471E-05	moderately acetylated
RBFOX3	2.91527732	0.76010519	2.704254134	4.68478E-05	0.000887648	moderately acetylated
COLQ	1.142989778	0.902354498	3.202961532	0.00021597	0.003965322	moderately acetylated
GNG11	1.928666703	0.693552468	2.45928797	0.000548617	0.003151414	moderately acetylated
LOC1001	9.371338326	1.140187269	4.03793335	7.20974E-05	0.001309009	moderately acetylated
CHDH	2.060679663	1.556259572	5.506909078	0.000653971	0.006196205	moderately acetylated
PLEKHF	2.967059982	0.651181662	2.304224623	0.00137816	0.00622505	moderately acetylated
SCARNA	1.118038163	1.450788381	5.130447917	0.003429326	0.03039821	moderately acetylated
CD3EAP	1.821032834	0.995546247	3.519973773	0.000519909	0.003368788	moderately acetylated
HOXC11	2.408817201	0.815372028	2.881454862	0.000102934	0.008861054	moderately acetylated
TEX22	1.126977753	0.925582158	3.267163241	0.002522283	0.018924204	moderately acetylated
PRKACA	3.6872031	1.575734929	5.558230315	5.33095E-05	0.00348062	moderately acetylated
TPR	7.128503006	2.92445553	10.31420744	2.98395E-06	0.000211813	moderately acetylated
TCHH	1.030286572	1.016644946	3.584659429	3.93275E-05	0.002295179	moderately acetylated
PHLDA3	1.911231122	1.933743763	6.81734271	3.30914E-05	0.004739589	moderately acetylated
PPP1R12	2.204367647	2.056562435	7.24840028	0.000106789	0.002915183	moderately acetylated
RFNG	10.87884161	1.136300735	4.00301456	0.000300422	0.004270395	moderately acetylated
GNG8	1.353090098	0.739049086	2.599644491	0.029768819	0.054817329	moderately acetylated
CLSPN	3.357681306	5.294152457	18.60776115	5.35081E-06	0.001065348	moderately acetylated
PCF11	2.129525866	0.591186774	2.076299389	0.001948503	0.005074981	moderately acetylated
CCDC13	1.601873798	3.880146852	13.60865212	2.08179E-06	0.000139194	moderately acetylated
GNA12	2.467184415	0.713845236	2.499838829	5.82978E-06	0.007681124	moderately acetylated
ZC3H4	1.389211158	0.719832974	2.520724614	0.000168116	0.003812042	moderately acetylated
KDM4B	2.025728479	1.773758466	6.209776499	3.13175E-06	0.000246228	moderately acetylated
ARHGEF	1.569588247	0.900181677	3.148031934	8.48938E-06	0.022870321	moderately acetylated
COL18A	1.886946203	1.516135354	5.298995717	0.000172066	0.006726406	moderately acetylated
PHF7	4.13912171	0.73791822	2.575369745	8.27856E-07	0.040978516	moderately acetylated
ZMYND	3.759040719	0.82336902	2.869422141	3.76846E-07	2.46036E-05	moderately acetylated
RLF	3.267661691	0.825905911	2.875729679	0.000275653	0.00424416	moderately acetylated
FAM57A	6.104511718	1.210788764	4.207484673	1.04441E-07	0.021416234	moderately acetylated
FOXC1	4.880613825	3.325266237	11.55468018	4.43033E-06	0.003489114	moderately acetylated
ZNF503-	2.025010697	1.254961486	4.359988918	0.000354502	0.008755917	moderately acetylated
C11orf95	1.795494977	0.752893069	2.614876999	0.00020218	0.000917178	moderately acetylated
C1orf162	1.502691885	0.665472417	2.302542959	0.005382976	0.018848396	moderately acetylated
GSDMB	1.997882655	1.095153826	3.784413409	0.001288604	0.007757605	moderately acetylated
KCNC4	2.125288953	1.295820398	4.47006922	0.000231861	0.006979704	moderately acetylated
SRRM3	2.766479396	1.174462683	4.045138578	1.97362E-08	0.00900315	moderately acetylated
PTPN23	3.016855544	0.661673575	2.277811677	5.68484E-05	0.001229675	moderately acetylated

SLC9A3I	4.237959884	2.18986886	7.524696764	4.53494E-05	0.003420746	moderately acetylated
C8orf58	1.917004847	0.631225274	2.165529556	5.48907E-05	0.001097083	moderately acetylated
ZFAND3	6.455026125	0.738967937	2.534888655	3.31537E-05	0.000406609	moderately acetylated
RPS6KL1	2.014682187	0.744546303	2.553161221	0.000169936	0.003597189	moderately acetylated
INHA	1.430903896	0.698858954	2.394976626	0.000172615	0.002239132	moderately acetylated
CLCN6	1.434412603	0.738148785	2.529578456	0.000287421	0.002461746	moderately acetylated
DLG5	1.96617601	0.735179855	2.514144635	8.17174E-06	0.003019345	moderately acetylated
C11orf84	4.874443952	1.199944495	4.102527003	3.84729E-05	0.001180784	moderately acetylated
SEMA6C	1.208613341	2.494642983	8.52770696	4.91358E-06	0.000606564	moderately acetylated
SOCS1	1.301266531	0.97204166	3.32034398	0.000833653	0.008027826	moderately acetylated
FUK	2.195932013	0.730500675	2.490513808	0.000383437	0.001446926	moderately acetylated
STAC2	1.702164959	1.153464096	3.928432236	0.000106556	0.00149175	moderately acetylated
ESRRA	6.932805723	0.76612449	2.609095661	0.002767481	0.007415043	moderately acetylated
EDARAI	3.008116505	0.589560212	2.006208893	3.00365E-05	0.000183455	moderately acetylated
PRR15	1.334941854	0.749096297	2.548926292	3.42506E-05	0.001282888	moderately acetylated
GAK	5.124563365	0.653074598	2.220117707	0.0001828	0.000483886	moderately acetylated
CBX4	4.539288727	2.523281522	8.577824322	3.33914E-07	0.000355349	moderately acetylated
LOC4013	1.328681911	0.76976314	2.616208192	2.9266E-05	0.00103618	moderately acetylated
PHLDA2	36.19657493	0.609021831	2.069330315	0.000564932	0.00467485	moderately acetylated
NCOR1	4.614929242	1.565528975	5.299454719	1.35959E-06	0.000276018	moderately acetylated
NECAB2	1.657469218	0.684650752	2.315677091	0.000364097	0.004461522	moderately acetylated
ATRIP	2.82836625	0.642250179	2.170769085	0.001101283	0.005291744	moderately acetylated
ASPDH	1	1	3.377069332	1.92371E-08	5.66291E-05	moderately acetylated
ANXA9	1.279855859	0.923774227	3.118930661	4.39041E-05	0.004963238	moderately acetylated
WNT3A	1.964583764	0.914159203	3.081715836	0.000909344	0.005544342	moderately acetylated
ZBTB45	3.171698035	0.844793102	2.84617502	0.002622229	0.022964448	moderately acetylated
MC1R	1.697168843	0.777889165	2.620158168	0.000189732	0.0149165	moderately acetylated
CDC42E	1.099273122	1.804485697	6.074947928	8.62964E-05	0.001474617	moderately acetylated
ATG4B	14.72227077	0.813339822	2.736248596	2.63132E-05	0.001313355	moderately acetylated
C20orf16	1.206131438	0.829097036	2.782498645	0.002300772	0.016024988	moderately acetylated
PLCB4	1.697328627	0.748029711	2.51020392	3.35303E-05	0.00021645	moderately acetylated
CATSPE	1.229747268	0.813175216	2.723956027	0.000295195	0.005733809	moderately acetylated
KIF20B	4.851607231	1.652680364	5.536072872	5.0548E-08	0.030113494	moderately acetylated
LOC3387	3.187813881	0.71013202	2.3785077	0.000907963	0.028514319	moderately acetylated
RSF1	4.279566473	5.429255632	18.18226101	5.73169E-06	0.000292405	moderately acetylated
RFPL3-A	1.048504172	0.953739648	3.19248037	9.1319E-05	0.003766763	moderately acetylated
N4BP1	2.576119424	0.700550152	2.342059154	3.10931E-05	9.99763E-05	moderately acetylated
NOL8	6.312292618	1.518530093	5.076463888	1.70229E-05	0.000591578	moderately acetylated
HERC2P	2.453207925	0.873352275	2.917988159	6.50096E-07	0.028168777	moderately acetylated
TYSND1	4.629369817	0.631125914	2.106918266	0.000918611	0.00872138	moderately acetylated
GPR143	1.014181061	0.98601723	3.286512488	0.000884888	0.012236259	moderately acetylated
CCNY	3.487085478	0.627271739	2.090707214	0.000248508	0.000647616	moderately acetylated
XIST	35.03935767	1.471256872	4.902220848	1.09505E-06	4.80194E-05	moderately acetylated
ARFGAF	9.879938204	1.596974505	5.319823359	6.24575E-05	0.001081991	moderately acetylated
PI4K2A	3.135196521	0.679141328	2.259094765	0.000355504	0.004090596	moderately acetylated
KIAA152	5.168259003	0.759692908	2.509794	0.000389874	0.002536929	moderately acetylated
LOC3881	1.48858846	2.022637169	6.676793552	1.43644E-05	0.044564848	moderately acetylated
LOC1002	1.184763745	0.872069763	2.877897589	0.000176267	0.002820107	moderately acetylated
PDXDC2	2.417272547	0.732830708	2.414802026	1.66751E-05	0.004320316	moderately acetylated
RHBDL1	1.177210347	0.84946586	2.799067034	0.01082418	0.039404831	moderately acetylated
C2CD2L	2.840920762	0.854388528	2.811970316	7.66077E-06	0.000303523	moderately acetylated
REPIN1	5.228061434	1.298183273	4.261203714	1.73181E-05	0.000412712	moderately acetylated
SNRNP7	12.71025803	6.500439877	21.33551047	3.41852E-07	0.000440708	moderately acetylated
HIST1H2	1.925797118	0.629407864	2.065708161	0.009917146	0.00455434	moderately acetylated
TRIM7	4.086658823	1.00017598	3.281828914	1.35981E-05	0.001975253	moderately acetylated

CELSR3	1.537119534	0.786663724	2.578642769	0.000139021	0.001946212	moderately acetylated
BRPF1	3.143914893	0.660827567	2.163875952	0.000255203	0.000714607	moderately acetylated
FLJ2735	1.677968527	0.759465035	2.486008835	0.0032842	0.015837529	moderately acetylated
NAT14	10.27496351	4.106844541	13.41684757	1.55466E-08	1.20325E-05	moderately acetylated
MICALL	5.230619643	0.95410287	3.111536966	0.000953808	0.008620876	moderately acetylated
MXD4	4.302327005	1.863563247	6.076556669	6.64491E-07	8.1533E-05	moderately acetylated
EIF3A	19.95051687	5.108147739	16.55081443	1.3992E-07	0.000256424	moderately acetylated
KIF15	3.757297478	1.058937926	3.430726312	9.29126E-05	0.002089467	moderately acetylated
WDR60	3.380343103	4.339229267	14.05659044	2.98042E-07	0.000289699	moderately acetylated
LAMB2P	1.49755417	0.715721828	2.318292609	0.001044865	0.004138786	moderately acetylated
LUC7L3	30.19700603	5.70845143	18.48334875	3.47384E-05	0.001148428	moderately acetylated
NTN1	2.550637225	0.684780729	2.216756341	7.16213E-07	0.004413612	moderately acetylated
MSI2	2.337569329	1.014836845	3.283141125	2.56878E-06	0.013160524	moderately acetylated
WNT5B	1.739154763	1.005862811	3.250561156	4.09476E-05	0.001346481	moderately acetylated
HTATSF	22.32717757	2.531667391	8.17623763	5.79397E-08	0.004401859	moderately acetylated
SLC25A4	1.545452192	0.678432613	2.18988822	0.002065364	0.004732867	moderately acetylated
FER1L4	1.233447627	0.945396766	3.05124789	1.57546E-05	0.000225881	moderately acetylated
PYROXI	1.213485738	0.824072314	2.659418111	0.000219998	0.004921621	moderately acetylated
MAP7D1	6.173798191	2.705454089	8.729500198	3.45872E-05	0.002386568	moderately acetylated
C3orf19	8.443469382	0.623007036	2.008162755	2.91499E-05	0.000287303	moderately acetylated
C12orf53	1.24238333	0.834299376	2.688399968	0.004985545	0.024240126	moderately acetylated
ARHGEF	2.042514171	0.640326952	2.061091308	8.49471E-05	0.000140167	moderately acetylated
LOC1515	1.442237995	0.693366839	2.231538226	3.2185E-05	0.000749528	moderately acetylated
SMC3	27.93253447	1.173795017	3.775634251	1.55916E-07	0.011332276	moderately acetylated
BBC3	8.544366327	1.19563303	3.84028405	5.04365E-05	0.001023872	moderately acetylated
MAMDC	1.137560509	1.087043645	3.48369897	0.000756198	0.012254744	moderately acetylated
MAL2	1.40997634	0.788959348	2.527808328	0.000158446	0.000933433	moderately acetylated
PAQR4	4.21763104	0.715116637	2.288972231	0.000286161	0.000901736	moderately acetylated
CEACAM	1.459945034	0.684957294	2.190643003	0.012039253	0.02970988	moderately acetylated
FADS3	5.720210713	0.78677098	2.514367856	0.000188493	0.002198223	moderately acetylated
KIAA091	1.784829772	1.002764668	3.196548259	2.33324E-05	0.002034507	moderately acetylated
PTPN18	5.002050976	0.647562313	2.060636549	0.000240884	0.000602555	moderately acetylated
TRIM11	9.416809005	0.658099652	2.086799964	0.000286028	0.000863111	moderately acetylated
DOC2A	3.261252864	1.568224833	4.963952609	5.00381E-07	6.56083E-05	moderately acetylated
CBLN1	1.877613066	0.890685191	2.814474984	0.000138106	0.002945305	moderately acetylated
RAB11B	18.05521016	1.016051176	3.205649559	9.22825E-05	0.003864994	moderately acetylated
LRP3	3.07766425	1.843631894	5.811750392	3.61976E-06	0.025263474	moderately acetylated
LOC7283	3.689669719	1.432620428	4.513475078	1.49018E-05	0.000688201	moderately acetylated
EXOC3	5.461286244	1.034047505	3.257389392	0.000456991	0.003517674	moderately acetylated
TMEM22	1.06025154	0.94317241	2.968302218	0.002781759	0.021022905	moderately acetylated
RTF1	5.32143143	2.878969579	9.052354715	9.44953E-07	0.001675596	moderately acetylated
ADAMTS	1.713145525	1.297694803	4.078318001	1.22277E-06	0.019088156	moderately acetylated
MAPK8I	1.798740463	2.962422692	9.304620647	2.88809E-08	0.005010679	moderately acetylated
RPS28	2750.509194	0.977179378	3.06794551	8.15299E-05	0.003403564	moderately acetylated
RARRES	1	1	3.13879653	0.004707875	0.029513259	moderately acetylated
SAP30	4.609209951	3.79692641	11.90735703	6.59388E-06	0.000491772	moderately acetylated
NISCH	4.562933351	0.854475435	2.675476742	1.41091E-05	0.000472778	moderately acetylated
LGALS9	1.080234429	0.925724985	2.897229635	0.001392935	0.014399234	moderately acetylated
EPB41L4	1.685537052	0.767022101	2.39820434	0.001864563	0.006645811	moderately acetylated
LOC1005	1.149591034	0.869874565	2.717057646	0.001296898	0.012905669	moderately acetylated
LINC000	1.328460077	0.894336727	2.785311503	0.001101422	0.0049922	moderately acetylated
ACCN3	1.480607756	1.43720423	4.47556183	1.84358E-05	0.041221397	moderately acetylated
E2F4	15.7461242	0.770518568	2.397446693	0.003046953	0.016645707	moderately acetylated
PRRG2	1.201816645	0.832073681	2.581042232	0.000601948	0.007640715	moderately acetylated
RASGEF	1.478491421	0.744024257	2.306052197	0.002030785	0.006980235	moderately acetylated

DOK3	1.019245969	0.981117444	3.039832953	5.41209E-06	0.000908779	moderately acetylated
COL5A1	1.546025875	0.763373502	2.364283312	7.27685E-05	0.001175183	moderately acetylated
MAP7	2.80579842	1.247114245	3.858097394	1.0239E-05	0.000954184	moderately acetylated
HAP1	1.396933318	0.973630971	3.011563542	3.41104E-06	0.023665792	moderately acetylated
KCNQ2	5.552684275	0.827544411	2.556108875	5.2262E-05	0.018610073	moderately acetylated
FAM149A	1.254669555	0.797022607	2.460717048	0.001011518	0.01021103	moderately acetylated
RASL10I	1.024646409	0.975946425	2.992596045	0.001256369	0.014468645	moderately acetylated
KDM5C	4.747281742	1.126284946	3.438755254	7.97128E-06	0.000351582	moderately acetylated
MPPED1	1.486399601	0.672766596	2.051171622	0.001270655	0.007373862	moderately acetylated
CNNM1	1.493154337	0.888095558	2.706431633	0.000271993	0.005325059	moderately acetylated
ZNRF1	1.608463315	2.173950944	6.619345516	3.8893E-06	0.008154918	moderately acetylated
PVRL2	14.95847745	0.853636702	2.59555862	2.79655E-05	0.001237054	moderately acetylated
TSC22D4	6.00288863	1.411561616	4.287581568	4.09239E-05	0.000705267	moderately acetylated
MTSS1L	1.804131631	2.642395377	8.025943389	0.000196114	0.003564545	moderately acetylated
HSPB9	1.170596048	0.854265655	2.593154618	0.010728634	0.038530677	moderately acetylated
C7orf43	2.157895347	0.82253663	2.49632241	0.000910708	0.008351349	moderately acetylated
RP1L1	1.033191096	0.99414002	3.016242778	8.12939E-06	0.001132246	moderately acetylated
LOC1001	1.026213827	1.164942294	3.531105359	0.000523	0.004897738	moderately acetylated
MALAT1	14.40946161	3.89572744	11.799624	4.12966E-05	0.002607414	moderately acetylated
LOC1001	2.470287119	1.394795827	4.213920823	8.40584E-05	0.003109701	moderately acetylated
SMPD1	1.886531244	1.110851692	3.353787714	0.000198849	0.004660571	moderately acetylated
TOP1	17.37813109	2.262988461	6.832127698	1.87E-05	0.002216902	moderately acetylated
SHANK3	1.313023434	0.785229763	2.3698268	0.000702543	0.006621512	moderately acetylated
EVL	4.601598628	2.518859655	7.601890577	2.26235E-06	0.000886117	moderately acetylated
PKDCC	2.266275079	0.747704693	2.255810872	0.000768629	0.004206735	moderately acetylated
LOC5414	1.111957224	0.89931517	2.709863065	0.009905473	0.038671196	moderately acetylated
NSUN5P	9.679078534	1.7480755	5.266623327	6.91414E-06	0.043456941	moderately acetylated
CERS1	1.346745486	2.071869024	6.240179224	1.835E-07	0.030219476	moderately acetylated
DHFRL1	2.332040633	0.900269924	2.704138697	3.56845E-06	0.044697646	moderately acetylated
RPS6KA	5.834728487	0.837939175	2.516815034	0.000307368	0.003422642	moderately acetylated
KIAA066	4.493259023	2.50070548	7.509753861	1.03976E-05	0.000458429	moderately acetylated
OLIG2	1.672367052	1.436968128	4.313919541	0.000204828	0.039062513	moderately acetylated
LGI3	1.153125922	0.926068245	2.772334518	0.003123281	0.018219475	moderately acetylated
ZNF638	6.711577721	1.662869244	4.972988754	7.17485E-06	0.001475832	moderately acetylated
PHF2	1.645722597	1.035307347	3.095980964	0.0002562	0.001905081	moderately acetylated
PFKFB1	1.227427791	0.814711877	2.436171234	0.000325259	0.005378328	moderately acetylated
NIPAL2	1.490108516	0.928778082	2.77579371	0.001441411	0.014709279	moderately acetylated
ZBTB4	1.392586569	1.115044681	3.330065047	6.46787E-06	0.008401656	moderately acetylated
CRIPAK	1.078569696	0.927153808	2.764044983	2.23946E-05	0.001646532	moderately acetylated
VIPR1	1.655657588	0.699610358	2.082263815	0.000920458	0.002453839	moderately acetylated
ATG2A	1.881629711	1.134665616	3.376796102	3.74811E-05	0.000939927	moderately acetylated
OGFRL1	2.648286457	1.247233611	3.711719666	8.63758E-05	0.002870046	moderately acetylated
RHPN1	2.366588312	1.297499076	3.85742287	1.47044E-06	0.010577686	moderately acetylated
C15orf37	1.447736062	0.69073364	2.051964777	0.000854615	0.006933771	moderately acetylated
CHD1	4.349673696	1.242525704	3.686370329	0.000422193	0.005265866	moderately acetylated
JRKL	3.574409745	0.860206487	2.549781608	0.000205129	0.014144901	moderately acetylated
MPP6	3.469014727	2.353687222	6.975077736	9.82606E-06	0.001163887	moderately acetylated
LOC2193	1.353417773	0.799592515	2.367827082	4.04684E-05	0.00157843	moderately acetylated
FLYWCH	2.886860967	1.099709715	3.25542549	0.000241167	0.004346808	moderately acetylated
PHC2	6.441142227	0.979661589	2.893073607	1.42431E-05	0.000625645	moderately acetylated
ACTA1	1.078741591	0.995871122	2.940598074	0.000151662	0.001440942	moderately acetylated
CDC42B	1.834015511	5.396155128	15.93297858	6.4081E-06	0.001913628	moderately acetylated
ARID4B	5.39406626	2.190582824	6.46802745	2.62731E-07	0.00383065	moderately acetylated
CD37	1.071745325	0.933057487	2.753956686	0.008345537	0.036961346	moderately acetylated
DPP9	4.701268477	0.736173027	2.171940518	0.000315659	0.013662762	moderately acetylated

PPP1R35	11.18053281	2.818419501	8.314889739	1.10667E-05	0.000458242	moderately acetylated
LENG9	1.100084373	1.009252348	2.972114697	1.50786E-06	0.02847768	moderately acetylated
HOXC9	6.850602136	1.683369175	4.956587041	7.69328E-05	0.004818359	moderately acetylated
RDH10	2.50770078	0.825351725	2.422873094	4.51429E-05	0.0032519	moderately acetylated
RASEF	2.073543782	0.692650952	2.0324388	0.001031462	0.003613709	moderately acetylated
MDN1	1.824931443	2.942734164	8.633564094	4.94274E-06	0.000259521	moderately acetylated
PDCD7	3.096762413	4.212899403	12.35628904	2.12391E-05	0.000984507	moderately acetylated
NYNRIN	1.549299817	0.752578007	2.206752505	2.05914E-06	3.17504E-05	moderately acetylated
FLJ1282	1.358469006	0.777517879	2.279645405	0.001538883	0.007669296	moderately acetylated
HMHA1	1.520652026	0.707288386	2.072905207	4.16888E-05	0.000779479	moderately acetylated
TNRC18	1.154923146	1.107590969	3.244772009	9.94591E-05	0.001225752	moderately acetylated
SPTBN1	3.097573521	1.774090251	5.1938183	2.03533E-05	0.002279449	moderately acetylated
HOXD13	9.745933643	1.081771706	3.1645287	3.5677E-06	0.002739937	moderately acetylated
AGAP11	1.513939679	0.798667102	2.335393077	0.007156239	0.019527284	moderately acetylated
MEGF8	1.425267674	1.064936022	3.112513482	6.62403E-06	0.000565463	moderately acetylated
GUSBP1	3.126048288	1.433839683	4.187667165	7.80878E-05	0.004675725	moderately acetylated
NOX1	1.437777941	0.695517695	2.028619452	0.003954476	0.015976656	moderately acetylated
FLJ2218	1.205331592	1.424124471	4.151375774	3.79992E-06	0.000140759	moderately acetylated
TF	1.186144545	0.84306757	2.456271198	0.000144788	0.003950886	moderately acetylated
MAP1A	1.377830482	0.862656551	2.506890745	1.2481E-05	0.000203472	moderately acetylated
GPR153	1.491775954	0.93567188	2.718483398	8.74903E-05	0.001116159	moderately acetylated
ZNF837	1.101653661	0.907726299	2.630661029	0.002059217	0.017218058	moderately acetylated
APOLD1	1.296250939	2.092899759	6.064360954	0.000103725	0.003474154	moderately acetylated
LOC1001	1.347879667	0.771978436	2.236416292	2.32261E-05	0.001381684	moderately acetylated
C12orf65	15.83727271	1.290596041	3.736964776	2.6497E-06	0.004501556	moderately acetylated
SUPT6H	6.016495178	1.977385126	5.723913359	1.87757E-06	0.008621032	moderately acetylated
OGFR	1.913110917	5.609022416	16.20848018	5.71872E-05	0.003229752	moderately acetylated
CCDC78	1.289897639	0.828593023	2.393472826	0.000149878	0.001038469	moderately acetylated
ZNF594	1.249783982	0.800138275	2.310893923	0.004619536	0.022207623	moderately acetylated
GPR135	1.036316813	1.020961335	2.94700306	3.52437E-06	0.000495086	moderately acetylated
YTHDF1	5.635116983	0.900376705	2.598924814	0.000545281	0.004049494	moderately acetylated
TEX29	1.046773866	0.95531617	2.756376155	0.048073464	0.101804378	moderately acetylated
LOC2541	3.959996521	0.847762744	2.445910038	0.000386784	0.002258509	moderately acetylated
DNM1	2.287523034	3.610251111	10.39889924	9.13463E-06	0.004994194	moderately acetylated
PHF12	2.496723196	1.124675374	3.238599773	8.6741E-06	0.037873307	moderately acetylated
FAM100	3.108818127	6.323258059	18.20813424	2.22387E-08	0.040271301	moderately acetylated
LOC643	1.059425641	0.943907681	2.717077044	0.008020601	0.036843787	moderately acetylated
ARHGEF	1.180072083	0.954470227	2.74641288	8.918E-05	0.000891017	moderately acetylated
NFATC2	5.688697668	2.734164905	7.852161183	4.7683E-07	0.00837115	moderately acetylated
PPP6R2	8.114280948	0.934640591	2.683182816	9.09665E-05	0.002936791	moderately acetylated
TP73	1.644836266	0.763736522	2.190693987	0.00095579	0.005007601	moderately acetylated
PRR14L	2.030383281	0.925514252	2.654159273	6.12044E-06	0.000737711	moderately acetylated
LOC1001	1.065901633	1.027172244	2.945423666	1.10274E-05	0.002113811	moderately acetylated
LOC1001	1.831260816	0.740062308	2.118195838	0.00266203	0.02329556	moderately acetylated
PVT1	6.766104747	0.824884459	2.359930842	3.32547E-05	0.000955351	moderately acetylated
FOXF1	3.802806929	0.94516488	2.702811776	0.000695778	0.014834913	moderately acetylated
IRAK1	16.11366263	1.87306085	5.348752996	2.23484E-07	0.020152459	moderately acetylated
F8A1	1.085569404	0.921175556	2.628805328	0.00013095	0.004145071	moderately acetylated
ORAI1	6.47100201	2.500721299	7.134947017	0.000188267	0.007926115	moderately acetylated
CIRBP-A	1.332851115	1.054020631	3.0068546	0.000103645	0.017609657	moderately acetylated
TPRN	2.512362987	3.202594433	9.130750615	0.000393328	0.006771118	moderately acetylated
BEGAIN	1.412073937	0.736141313	2.09764756	0.002286941	0.010009866	moderately acetylated
CCDC11	1.40707678	0.83822293	2.387925486	0.000388214	0.002633729	moderately acetylated
LOC100	1.193428348	1.202349528	3.42401864	0.000396529	0.00381036	moderately acetylated
PLCB3	1.520717161	1.838903309	5.235383054	7.56689E-05	0.003536983	moderately acetylated

ENDOG	1.353835091	0.738642399	2.099163119	0.001321337	0.009293549	moderately acetylated
MMP9	1.314905561	0.760510891	2.161196341	0.001911762	0.008797169	moderately acetylated
VSTM2L	1.319129818	0.929463342	2.639770783	0.000180859	0.014749517	moderately acetylated
ZNF320	1.408694494	0.875126757	2.483976206	1.41322E-05	0.022601435	moderately acetylated
RPS6KA·	1.805006185	0.968107439	2.746826381	0.0006753	0.030861989	moderately acetylated
KCNG1	3.880226391	1.463332576	4.145503793	0.000894691	0.016705238	moderately acetylated
SAMD10	1.655143306	0.724109004	2.048375105	0.000112011	0.001088567	moderately acetylated
DNASE1	1.015489171	0.984747084	2.78387235	0.000455982	0.008641171	moderately acetylated
LOC100[illegible]	1.164665037	1.083170098	3.059977219	0.000247428	0.005611913	moderately acetylated
GUCY1E	1.405050837	1.677906175	4.738740868	1.45622E-06	0.000208045	moderately acetylated
RSAD1	19.29428466	0.735703253	2.07688989	0.000180079	0.011294002	moderately acetylated
PAX2	1.226592807	0.835726918	2.358256586	0.003814713	0.018790398	moderately acetylated
LSM14B	2.338435663	3.030152114	8.544187425	6.4342E-05	0.005524865	moderately acetylated
GPR35	1	1	2.818358005	0.004401159	0.028485561	moderately acetylated
ZNF883	1.256761174	0.832767439	2.345866499	1.48678E-05	0.000158799	moderately acetylated
GAS2L1	1.303718977	0.85008495	2.387754245	0.001167444	0.00991733	moderately acetylated
FAM133I	1.601562226	0.790003513	2.218570855	0.000540787	0.002264342	moderately acetylated
LOC1001	1.191288431	0.839427274	2.351946716	0.000547872	0.007487772	moderately acetylated
CTU1	1.184184017	2.016744995	5.638696059	0.000142969	0.006076572	moderately acetylated
FAM90A	1.176983098	0.849629873	2.372479458	0.021139787	0.054951229	moderately acetylated
SUN2	5.965309079	0.926694194	2.584551145	0.000147585	0.001176875	moderately acetylated
COLEC1	2.482815216	0.848552233	2.365531429	0.000110865	0.000824538	moderately acetylated
PGRMC[illegible]	11.32580763	1.324828727	3.691501252	2.02983E-05	0.001068298	moderately acetylated
PSD	1.009820053	1.068993532	2.972081708	0.000225992	0.002280253	moderately acetylated
PBX1	2.901278167	1.188263614	3.293513176	7.04025E-06	0.000109494	moderately acetylated
SOLH	2.273759694	1.485271247	4.114572229	3.39832E-06	0.001562974	moderately acetylated
RP1-177[illegible]	1.320299834	0.797995214	2.210402047	0.000163753	0.00026823	moderately acetylated
MAP3K1	2.039671749	2.303340981	6.378267413	8.59973E-05	0.002011417	moderately acetylated
SCN4A	1.451122328	0.775358001	2.14601205	0.001076348	0.009130879	moderately acetylated
TMEM1[illegible]	1.664417421	0.727893744	2.014584173	7.68526E-06	6.61287E-05	moderately acetylated
DUSP8	1.352517653	2.328608542	6.425282783	6.75405E-06	0.000393664	moderately acetylated
PARD6G	1.479738299	0.733487334	2.02377188	0.000218351	0.001315621	moderately acetylated
CREG2	1.380612051	0.860252812	2.371456527	0.000487866	0.003579212	moderately acetylated
ZNF426	1.725681003	0.822516844	2.266271092	0.002327116	0.005873528	moderately acetylated
ANKRD[illegible]	1.563780831	1.425727553	3.927460905	0.00037795	0.010510588	moderately acetylated
MIR631	1	1	2.753208588	0.016508483	0.057926159	moderately acetylated
LOC100[illegible]	1.207113425	0.828422565	2.279085004	0.001307051	0.011175586	moderately acetylated
ARHGEI	2.141317766	0.963999331	2.651854105	1.02359E-06	0.037574814	moderately acetylated
SNRNP3	16.82481795	1.815409741	4.990994717	7.40135E-06	0.011524829	moderately acetylated
OBSCN	1.183282084	1.140742825	3.132159651	9.05438E-07	0.000233995	moderately acetylated
PKD2	2.070425256	0.862583377	2.361190803	5.60867E-05	0.00022607	moderately acetylated
SOX2	1.613468496	2.108815712	5.770071485	0.000348413	0.005421861	moderately acetylated
SFRP5	1.482284196	0.752190123	2.057206399	0.001713829	0.009561845	moderately acetylated
SOBP	1.359274658	1.481454138	4.048188172	0.000418805	0.009927149	moderately acetylated
ZFP36L2	3.334954012	1.014016812	2.76963072	5.18693E-05	0.029399701	moderately acetylated
LAMP1	4.915870417	3.335516349	9.092315833	5.0621E-06	0.007666472	moderately acetylated
VSTM2B	1.195518707	0.836457007	2.279352245	0.02017532	0.048910886	moderately acetylated
ZNF518E	2.109527196	0.826618192	2.252481819	5.98079E-05	0.006383223	moderately acetylated
MXRA7	5.721805272	0.969015124	2.637680585	6.8804E-06	0.000120606	moderately acetylated
GRAMD	8.651529676	1.008532266	2.743723998	6.43795E-05	0.002880093	moderately acetylated
MAPKAI	2.672691931	3.719443094	10.11567455	2.95998E-07	0.015697132	moderately acetylated
EXOSC4	82.70596654	0.841497177	2.28779684	0.005222706	0.019193156	moderately acetylated
ZDHHC[illegible]	3.237939781	1.780799919	4.83992492	2.97949E-07	0.002372418	moderately acetylated
CDK5RA	4.978067475	3.122335845	8.481884707	6.57489E-06	0.00124417	moderately acetylated
C19orf71	1.188888646	0.841121667	2.283258203	0.000528518	0.001130043	moderately acetylated

HCG11	2.461963774	0.866078898	2.347455788	0.000277494	0.011809763	moderately acetylated
ZNF598	3.739852631	1.67828488	4.548357137	1.0542E-05	0.000486245	moderately acetylated
PACS1	2.646980933	1.305410773	3.530525103	2.49368E-06	0.000103176	moderately acetylated
ZNF503	2.828277578	9.114093117	24.63478277	2.48733E-05	0.004601909	moderately acetylated
AHSA2	2.186665779	2.003600737	5.41122912	1.15829E-05	0.001812439	moderately acetylated
DNAJC2	14.15777985	2.069630905	5.580743656	2.48763E-06	0.000325419	moderately acetylated
APBA2	2.742927239	1.039327078	2.801427164	0.000200774	0.001875005	moderately acetylated
ARL8A	5.989810927	1.923299581	5.18041266	5.29522E-07	0.017521958	moderately acetylated
ADRBK1	5.175834399	1.269823017	3.415812964	0.000112011	0.001470889	moderately acetylated
ODZ1	1.33602595	1.138967385	3.060454746	1.65996E-05	0.00026867	moderately acetylated
C8orf84	1.097816484	0.910899057	2.44630241	0.000934285	0.010897902	moderately acetylated
ZNF414	2.834980787	1.08787428	2.920727608	2.41377E-05	1.30289E-05	moderately acetylated
EVC	1.187173404	0.885896267	2.377914991	0.000758543	0.006367951	moderately acetylated
LIMS2	1.20741207	0.828217661	2.222099486	0.0040337	0.020264891	moderately acetylated
LHX2	1.889276273	1.241811361	3.329504128	0.00028011	0.004227232	moderately acetylated
KIAA173	4.993155951	1.742298885	4.667088917	9.36678E-07	0.004330988	moderately acetylated
FAM84B	6.180033087	0.791059468	2.118824663	5.7356E-06	0.000213577	moderately acetylated
CTIF	1.974402507	1.002851241	2.683000181	0.000113226	0.001221994	moderately acetylated
LRRC4B	1.163800658	2.132643757	5.696522196	3.33648E-07	0.000102113	moderately acetylated
CCM2	7.384419207	1.051330001	2.806386243	0.000119753	0.026345453	moderately acetylated
C20orf16	1.195439936	0.91223003	2.434222224	0.000556041	0.002779572	moderately acetylated
KIAA201	4.873497985	1.810186782	4.826693475	0.000135894	0.007943183	moderately acetylated
PALM2	1.119423685	0.903675952	2.408522126	0.000419163	0.007115205	moderately acetylated
LRRC47	5.937395563	1.713174385	4.555505678	1.75561E-05	0.002539169	moderately acetylated
WNK2	2.047814379	0.983296473	2.61386265	0.001067721	0.013016391	moderately acetylated
GAR1	62.22149645	1.144559298	3.041779566	4.07334E-07	0.016863771	moderately acetylated
TAZ	9.053178706	1.350581789	3.58638347	2.91727E-05	0.002914366	moderately acetylated
GRIP2	1.243374196	0.85856996	2.279251514	0.000163936	0.003585696	moderately acetylated
MECOM	1.825253425	1.054646699	2.799349018	4.37874E-05	0.01099357	moderately acetylated
FOXA3	1.482262316	0.893149502	2.370120963	6.90308E-05	0.000948126	moderately acetylated
MAFB	1.580849831	1.568922735	4.155751747	0.000131498	0.006352049	moderately acetylated
LPCAT4	9.129863168	1.652908346	4.375271285	0.000187219	0.003490374	moderately acetylated
PATL1	4.868240731	0.785335391	2.075268859	0.000225752	0.035838858	moderately acetylated
LOC2835	1.148928405	0.870376253	2.298930561	0.0192871	0.049150669	moderately acetylated
POM121	3.416515217	1.323057378	3.493129135	0.000188185	0.005898768	moderately acetylated
POM121(	2.940162311	1.7934687	4.734581337	0.000127316	0.003372513	moderately acetylated
LOC1468	1.737979969	0.822986447	2.172164755	0.00041829	0.012454123	moderately acetylated
NAV1	1.874890665	1.532769696	4.043102529	1.12674E-05	0.002223345	moderately acetylated
MYO9B	1.631196573	1.798294802	4.734199518	0.002222808	0.028787931	moderately acetylated
MLL2	1.325838898	1.75233071	4.612059256	9.48706E-06	0.000347549	moderately acetylated
ANKMY	1.324993956	0.801547092	2.107820888	7.7634E-05	0.002514999	moderately acetylated
CHMP4E	3.107494149	8.269237611	21.73682189	0.000508165	0.021702077	moderately acetylated
HOXC6	1.935703278	1.325892896	3.478745758	0.001121789	0.012719032	moderately acetylated
ZNF814	1.300339494	1.154806966	3.027200852	3.60437E-08	0.024939208	moderately acetylated
LMOD1	2.827864857	1.531627139	4.009542338	8.85891E-05	0.002674514	moderately acetylated
NR2F6	22.85610688	1.109279606	2.9014664	2.57032E-05	0.00197828	moderately acetylated
ARHGDI	12.00952649	3.23922423	8.463620113	0.000955712	0.015719353	moderately acetylated
ZFHX3	1.22654783	1.33349471	3.482945934	1.30639E-05	0.002555457	moderately acetylated
KLF2	1.196522644	2.096122898	5.473245141	4.97061E-05	0.004380869	moderately acetylated
SREBF1	1.921478062	1.002866854	2.617934933	0.000469323	0.00373694	moderately acetylated
CCDC13	18.97693337	1.405538352	3.664545431	1.45777E-06	0.025665159	moderately acetylated
PXN	7.621668187	0.869807603	2.267418239	1.10978E-07	0.035442862	moderately acetylated
SPPL2B	6.602611336	0.864513882	2.251515852	0.000287995	0.005144678	moderately acetylated
PHF23	7.924272933	2.575061289	6.706236618	4.79702E-08	0.008559631	moderately acetylated
IL20RB	1.37704889	0.883549107	2.300256921	0.00027149	0.033224945	moderately acetylated

KDM2A	2.437220047	1.476344647	3.842593596	6.39898E-08	0.005079597	moderately acetylated
ADARB1	2.301762509	1.242508276	3.232131254	1.09109E-07	0.005285322	moderately acetylated
NKTR	4.404398728	4.166545008	10.82814298	8.12385E-08	0.001963732	moderately acetylated
DLX1	3.675218203	0.854662353	2.220849019	0.000169007	0.024953501	moderately acetylated
EPHB1	1.705910988	0.788137554	2.046624378	0.003338572	0.010155444	moderately acetylated
MSL1	4.380648186	1.027406179	2.665798848	4.77457E-05	0.028131543	moderately acetylated
SLC5A1(	1.588856944	1.010782519	2.620015841	0.004403143	0.026146454	moderately acetylated
CLIP1	3.473927098	1.758318661	4.553588698	1.30681E-06	6.24448E-05	moderately acetylated
SFPQ	8.779430883	4.873750448	12.61664629	8.99363E-06	0.010493992	moderately acetylated
C1orf51	1.041844279	1.018039065	2.635288527	0.001950185	0.014058392	moderately acetylated
MACF1	1.546790128	2.448939314	6.334220704	2.40016E-07	0.000674885	moderately acetylated
CYP27C	1.100386082	0.950761438	2.459106587	0.002092186	0.018098473	moderately acetylated
ARHGAI	1.967529775	1.650742522	4.257909922	6.90653E-05	0.017202633	moderately acetylated
OGT	18.42294868	1.12520392	2.900418982	5.40887E-06	0.014862827	moderately acetylated
MMD2	1	1	2.576452499	0.001798452	0.017877312	moderately acetylated
CCDC14	1.117488198	0.894864037	2.30462979	0.000555726	0.008391939	moderately acetylated
GLTSCR	1.168291757	1.111129263	2.861463671	0.000618632	0.004640242	moderately acetylated
FGF2	1.814412379	1.131922382	2.910093978	5.0376E-05	0.003167141	moderately acetylated
DAG1	2.219548587	1.676557925	4.309556656	0.000216788	0.007971204	moderately acetylated
GNAO1	1.434891091	0.800954961	2.058591178	2.46684E-05	0.00016011	moderately acetylated
AXIN1	4.684245153	1.052368566	2.703901283	9.82883E-05	0.002853967	moderately acetylated
ZCCHC1	1.780488467	1.109533862	2.845665088	7.34777E-06	0.020682607	moderately acetylated
DHX38	5.133849306	1.591020855	4.074873038	1.16301E-05	0.001257578	moderately acetylated
ZNF771	12.8256145	0.861938312	2.200578344	6.928E-05	0.000831708	moderately acetylated
PCYT1B	1.264126507	0.865396977	2.206810999	3.95634E-05	0.00038662	moderately acetylated
PKD1	1.630876593	0.851552513	2.169270933	0.001922816	0.011436959	moderately acetylated
USP22	11.74289777	1.149554108	2.92697845	1.47206E-05	0.000493747	moderately acetylated
SDF4	23.42033289	0.906781469	2.307685011	2.52876E-05	0.000292343	moderately acetylated
FUT3	1.088616212	0.99352634	2.520615832	0.008513102	0.034342049	moderately acetylated
ZNF853	1.151610405	1.106652034	2.805405041	3.62119E-05	0.001265098	moderately acetylated
B7H6	1.775389671	1.631752911	4.133740653	1.95037E-05	0.001614275	moderately acetylated
PFDN6	12.98888733	1.004196138	2.543294494	5.01017E-05	0.000950182	moderately acetylated
ZNF830	12.61398011	1.048571483	2.655508978	0.000227838	0.00998647	moderately acetylated
ZNF500	2.049555384	2.180655431	5.521430865	1.83126E-05	0.003619832	moderately acetylated
SSR4P1	1.145835107	0.872725921	2.209439096	0.000158924	0.004009394	moderately acetylated
OSBPL7	1.353661499	0.803821257	2.031445	0.007100802	0.024444627	moderately acetylated
NEURL	1.252226649	1.016509532	2.568221876	0.001585589	0.008773081	moderately acetylated
ACTL6B	1.064051135	1.007841817	2.545318351	0.000691971	0.005060825	moderately acetylated
C10orf26	1.81617615	0.943421775	2.378494964	0.005397206	0.029070112	moderately acetylated
NOXA1	2.996316948	1.35074579	3.401812403	0.00044518	0.011838196	moderately acetylated
KPNB1	39.25989237	2.579218899	6.492966388	2.98485E-08	0.01188204	moderately acetylated
LAT	1.022912879	1.09505769	2.756156318	0.000220368	0.003132247	moderately acetylated
CACNB1	2.267972388	0.800510111	2.014310094	0.008931959	0.029282253	moderately acetylated
SNORD1	1	1	2.514049043	0.01204068	0.048740228	moderately acetylated
LOC1002	1.213309094	0.824192289	2.07025666	0.0004827	0.006435503	moderately acetylated
HPS6	2.423796439	1.741511596	4.373343346	3.19344E-05	0.030227117	moderately acetylated
C12orf71	1.024897597	0.975707234	2.449691058	0.000285637	0.006495536	moderately acetylated
LMTK3	1.070450953	0.934185725	2.343890277	0.000113826	0.003900783	moderately acetylated
RRAGC	3.375872469	2.728553707	6.844491588	1.5261E-05	0.004573691	moderately acetylated
SMC4	32.20568026	2.016991092	5.057775213	6.9222E-07	5.70816E-05	moderately acetylated
FOXP4	1.748180499	1.604090717	4.016570717	9.88609E-05	0.002145243	moderately acetylated
LOC6415	1.220328274	0.865138578	2.163610113	0.01412186	0.038422766	moderately acetylated
UBL4B	1.04011767	0.961429681	2.404118653	0.0008714	0.011489468	moderately acetylated
CHGA	1.216127407	0.864797721	2.162338399	0.002838758	0.013156601	moderately acetylated
WNK4	1.076788271	0.953324598	2.383175765	0.000688065	0.008732524	moderately acetylated

WBP4	9.008568312	1.669393102	4.171688334	9.59544E-07	0.000376451	moderately acetylated
SOX12	1.905825249	8.207801721	20.49400554	9.65412E-07	0.000155796	moderately acetylated
TMEM2(	1	1	2.496668736	0.006008086	0.033571425	moderately acetylated
HSPA1B	11.06297012	1.183893332	2.954100915	5.67263E-06	0.000278361	moderately acetylated
HOXD10	1.716493454	4.949208335	12.34862299	6.23622E-09	3.10313E-05	moderately acetylated
ANPEP	1.02316398	1.036718138	2.586194996	4.32381E-05	0.000603872	moderately acetylated
ZHX3	2.011433846	1.522106414	3.794588909	1.1356E-05	0.023533466	moderately acetylated
ABCD1	1.536994854	0.822207421	2.047580222	0.000138639	0.000843881	moderately acetylated
CLEC4F	1.205943938	0.829225944	2.062606708	0.027154873	0.058354878	moderately acetylated
MSX1	3.886043065	4.944878664	12.29202634	4.77639E-06	0.000884604	moderately acetylated
LOC1002	1.368655946	0.984107898	2.445854361	0.003105196	0.019369454	moderately acetylated
ST7-AS1	1.071877986	0.987711385	2.453134287	0.000241391	0.001949596	moderately acetylated
METTL1	9.417283197	3.061225566	7.586584189	0.000164699	0.004834957	moderately acetylated
MCOLN2	3.777674958	0.847823437	2.098105466	0.003825875	0.018079664	moderately acetylated
NRL	1.261071934	0.837500582	2.07236605	0.018406492	0.042579901	moderately acetylated
LRRC37A	1.045123171	2.136860543	5.275143993	4.29887E-07	0.003308054	moderately acetylated
FABP7	1.083434978	0.922990322	2.277660766	0.000622859	0.00905959	moderately acetylated
KIF13B	1.304194609	0.842494316	2.0776783	0.000132988	0.003090755	moderately acetylated
SAV1	5.599982445	1.242092808	3.061819479	0.00023395	0.006317133	moderately acetylated
LOC1477	12.09129939	2.716977348	6.697422226	1.66241E-06	0.000698106	moderately acetylated
BEX1	7.020187251	2.542285536	6.263461139	8.44625E-06	0.001433541	moderately acetylated
LOC2833	1.59036753	1.131976653	2.787865784	0.000967989	0.010009172	moderately acetylated
MARK4	2.460273999	1.529897169	3.766530644	0.000100794	0.047346463	moderately acetylated
BRD7	9.254723391	4.025416264	9.893667192	5.15281E-07	0.009074856	moderately acetylated
LPCAT1	3.56722597	4.624507478	11.36571735	2.39188E-06	0.000482128	moderately acetylated
MPHOSI	15.63028242	1.4802052	3.636138873	4.08498E-06	0.000173879	moderately acetylated
CDC25B	12.37388825	1.274372801	3.126028203	2.68463E-06	0.012733436	moderately acetylated
HNRNPI	29.51882764	4.689920449	11.5036394	3.94414E-07	0.000112309	moderately acetylated
ACCN2	4.809490563	1.28229118	3.141175904	2.06426E-06	8.41625E-06	moderately acetylated
H19	1.116238221	0.93686779	2.291667497	0.001035097	0.008468294	moderately acetylated
HES6	16.68855475	1.63319493	3.99369981	1.19067E-05	0.000889925	moderately acetylated
HMX1	2.074757735	6.246769163	15.27041623	8.40299E-09	0.02551255	moderately acetylated
AKT2	5.293098302	1.583110259	3.869073403	3.60411E-05	0.000898142	moderately acetylated
CBX7	1.795384715	1.31630754	3.216809197	9.69396E-05	0.002999172	moderately acetylated
SLC25A4	1.114274921	0.89744459	2.19157096	0.000176285	0.002945441	moderately acetylated
CARD9	1.209326303	0.954392047	2.324366211	0.011098312	0.040087313	moderately acetylated
LOC4403	1.01628524	0.983975719	2.396054613	0.00183537	0.0175468	moderately acetylated
EFNB1	4.200077231	0.899037508	2.186304214	6.00317E-05	0.040646076	moderately acetylated
PRR7	9.402292019	1.801118555	4.374606505	0.000154094	0.006338605	moderately acetylated
ZNF580	15.11551068	2.871204228	6.972214327	2.5641E-05	0.00095071	moderately acetylated
FRAT2	3.151848615	2.189112226	5.314525979	2.9235E-05	0.00148697	moderately acetylated
DDX17	16.08256519	1.450789695	3.515754869	2.71539E-06	0.000486457	moderately acetylated
CIR1	16.08434268	1.687142764	4.083878186	0.000113105	0.00570189	moderately acetylated
PPIP5K1	2.928008808	0.970390478	2.348356558	0.000739727	0.007928093	moderately acetylated
ECM1	1.153356695	0.867034461	2.094606186	0.001164626	0.009894713	moderately acetylated
DDA1	11.3181471	0.833908012	2.014265272	2.71044E-05	0.000293089	moderately acetylated
CENPV	35.21985411	2.0463759	4.941516753	6.55566E-08	0.000268467	moderately acetylated
SLITRK3	1.340977823	0.973292683	2.349805667	9.87275E-06	0.004619745	moderately acetylated
HAR1A	1.020395493	1.096642488	2.647128563	0.000611535	0.004764798	moderately acetylated
RTN4RL	1.094612443	1.006567833	2.423913805	0.002416471	0.019241021	moderately acetylated
SIN3B	3.612881639	0.918440031	2.210881201	2.40515E-05	9.23206E-05	moderately acetylated
COBRA1	8.037020828	3.043417521	7.317857282	2.499E-05	0.001325438	moderately acetylated
SNAPC4	2.548213506	2.108806624	5.069214517	1.09741E-05	0.002396714	moderately acetylated
RBM10	13.68905751	1.608256333	3.863283848	4.73723E-06	0.000186417	moderately acetylated
TMEM74	3.312654387	4.168821741	10.009646	7.99585E-07	0.003032586	moderately acetylated

ZRSR2	3.971554008	4.674515491	11.22335952	0.00024065	0.010006263	moderately acetylated
LECT2	1.07524242	0.930022831	2.230697768	0.001361856	0.013814554	moderately acetylated
SNF8	47.23017864	1.288002153	3.087558379	1.79794E-05	0.000674403	moderately acetylated
MMP24	2.118990216	2.452707745	5.856943838	3.04659E-07	0.016380289	moderately acetylated
TRAF5	4.278154275	1.213146267	2.89526435	3.79751E-05	0.017599943	moderately acetylated
NEK8	1.598547899	0.935255575	2.229898448	0.000231656	0.002029375	moderately acetylated
MAP9	2.576337195	2.948286825	7.024535768	8.99106E-05	0.002880061	moderately acetylated
TRIM33	3.020551369	1.0919137	2.599709261	3.11463E-06	0.03187457	moderately acetylated
ZNF784	1.283824203	1.576358803	3.750209007	9.96073E-05	0.007010627	moderately acetylated
TUBA8	1.17979194	0.978222331	2.326803625	3.13286E-06	0.003098366	moderately acetylated
PCLO	1.210960089	2.138761548	5.085246287	1.6846E-06	0.016086385	moderately acetylated
FGFBP3	1.241515671	1.95660437	4.649673239	6.75089E-05	0.002519141	moderately acetylated
RSRC2	50.50705675	1.387875466	3.290812628	1.50841E-05	0.00044995	moderately acetylated
TCAP	1.018923347	0.981428095	2.326661547	0.001611707	0.016261209	moderately acetylated
BMS1	6.834597851	0.978681628	2.31989464	0.00013026	0.006199181	moderately acetylated
HIGD2B	1.117684603	0.983486288	2.330460855	0.001382059	0.006449123	moderately acetylated
MAP4	17.07050395	1.432554672	3.391885696	2.16591E-05	0.002509888	moderately acetylated
GIPR	1.230265246	1.156385001	2.736052	0.004178926	0.019977755	moderately acetylated
UPF3A	7.625751687	2.156036597	5.099033331	0.000386523	0.007776663	moderately acetylated
CCDC85	4.094108786	1.247149564	2.949068245	9.05077E-05	0.00709862	moderately acetylated
PLCE1	1.633811569	0.851655422	2.012951433	8.34415E-08	1.31363E-06	moderately acetylated
WAS	1.097256776	0.96396966	2.275704474	0.002733203	0.015096004	moderately acetylated
GP1BA	1.083557517	0.922885942	2.178633701	0.017983923	0.054033875	moderately acetylated
ETS2	3.524648869	0.863419124	2.036557305	0.00042557	0.001751082	moderately acetylated
FKBP2	93.34841386	1.035242981	2.439284555	9.01605E-05	0.010141932	moderately acetylated
LOC849	1.128181616	0.886382109	2.088336505	0.009558221	0.031701907	moderately acetylated
IL17B	1	1	2.355714614	0.036483917	0.090578566	moderately acetylated
PITX1	3.527015249	5.656169028	13.32071333	1.5168E-05	0.003181407	moderately acetylated
PTOV1	16.98747499	3.736894201	8.799150764	1.25255E-06	0.047414438	moderately acetylated
NRARP	4.149367367	2.727937789	6.421278821	6.30525E-05	0.018784478	moderately acetylated
MYH9	5.225043762	2.308241171	5.431628894	2.07128E-06	0.00132596	moderately acetylated
WRNIP1	8.527936001	1.513542935	3.56116056	1.22787E-06	8.62256E-05	moderately acetylated
CYHR1	10.62301837	1.194727295	2.80934327	1.16662E-05	0.009274971	moderately acetylated
CCKBR	1.144243023	0.873940221	2.054297752	0.001577838	0.00939407	moderately acetylated
ACSS3	1.099093555	0.941528099	2.212240345	0.008658682	0.034944671	moderately acetylated
HRCT1	1.140809641	0.876570432	2.0591948	0.032267229	0.067146785	moderately acetylated
ZNF260	2.372095665	1.217525467	2.859494033	0.001044148	0.012602064	moderately acetylated
MNT	1.566925772	2.383849867	5.580382002	2.67078E-05	0.003348767	moderately acetylated
BAP1	10.12016944	1.354216711	3.169550487	0.000303753	0.004000007	moderately acetylated
CMTM4	3.589684302	0.997067049	2.333251095	1.50815E-05	0.004960131	moderately acetylated
PPM1N	7.838007269	1.985488853	4.63903023	7.18718E-05	0.002581353	moderately acetylated
SELO	3.880135136	1.701919273	3.973772954	0.000208262	0.008768288	moderately acetylated
ZNF566	2.353566948	0.857623561	2.001113242	0.000927839	0.002688065	moderately acetylated
OXTR	1.040546541	0.984316813	2.292506347	0.002477119	0.019282188	moderately acetylated
GLI4	3.235545496	2.373450028	5.524612268	8.55035E-05	0.005037986	moderately acetylated
USF2	12.14729632	4.69982817	10.93188689	1.88064E-05	0.001611337	moderately acetylated
ENTPD1	2.708492318	1.282467123	2.978869303	2.5726E-07	0.033572886	moderately acetylated
MYLPF	2.729461794	2.191326601	5.087946218	0.000443841	0.020632786	moderately acetylated
ENTPD2	1.677787686	1.032000482	2.395997704	0.005066379	0.04053708	moderately acetylated
IWS1	17.47916675	0.986288303	2.289497638	2.31333E-05	0.00016233	moderately acetylated
EEA1	2.143996024	2.404629208	5.566771772	1.15807E-06	0.001198487	moderately acetylated
TEAD3	4.042268513	1.40620447	3.254042208	7.2848E-05	0.012175467	moderately acetylated
ANKS6	2.769074825	1.114892479	2.579037473	1.12089E-06	0.024082148	moderately acetylated
SHOX2	1.886653622	1.093511919	2.528945231	0.000158357	0.013452824	moderately acetylated
TACC3	23.21178962	1.034100899	2.391167321	7.92348E-05	0.013911578	moderately acetylated

PRPF38E	18.63467707	6.120596576	14.15145911	1.16937E-06	0.000230155	moderately acetylated
SPEG	1.32425862	1.354560319	3.131818619	6.79391E-05	0.011971691	moderately acetylated
BRCA1	6.450845877	2.00268203	4.630083769	6.84834E-07	9.76665E-05	moderately acetylated
LCN2	1.087777661	0.919305512	2.124225741	0.006863533	0.027485952	moderately acetylated
ZKSCAN	3.952952442	2.228882283	5.14809837	0.000186629	0.009078947	moderately acetylated
ZAP70	1.04032362	0.96123935	2.218037083	0.002092515	0.017722597	moderately acetylated
HBA1	3.345617892	0.975580765	2.246775777	0.000683992	0.009799401	moderately acetylated
ZBTB43	3.794695048	1.000874675	2.298602153	8.95144E-05	0.007971056	moderately acetylated
C22orf24	1.230528658	2.157919886	4.953069775	0.001421289	0.018702872	moderately acetylated
FAM110	2.924340114	1.03540671	2.374838842	0.000459321	0.003639731	moderately acetylated
PTAFR	1.028651608	0.972146441	2.229086811	0.00020964	0.00542595	moderately acetylated
RHOU	3.976098196	0.900057082	2.063000084	4.55737E-05	0.030004394	moderately acetylated
MBL1P	1.200409074	0.89788978	2.056670325	0.003761873	0.013325847	moderately acetylated
C1orf122	18.23893027	3.229964864	7.38887769	1.59566E-06	0.001002473	moderately acetylated
SLC25A2	2.743669226	0.911312501	2.084446593	0.001115978	0.00479912	moderately acetylated
C9orf78	33.24150523	2.832054892	6.474174083	4.34278E-06	0.001782104	moderately acetylated
ARID1A	2.249130609	1.909160381	4.360889302	1.67752E-05	0.002728326	moderately acetylated
TNKS	2.766609926	0.887303402	2.025367441	2.81057E-05	0.000670115	moderately acetylated
ZNF467	2.161222591	1.340629256	3.057488151	7.90416E-06	0.000484497	moderately acetylated
TSPYL2	12.45773551	1.072850756	2.446499407	1.36467E-05	0.015158164	moderately acetylated
MDC1	1.532534766	1.063301844	2.424110844	1.02569E-05	0.000231244	moderately acetylated
BAHCC1	1.157397148	1.020881708	2.32382487	9.50693E-06	0.000176007	moderately acetylated
EPHA8	1.945915217	1.0937075	2.488702301	0.000109196	0.040459897	moderately acetylated
TNKS1B	1.929393259	1.397048103	3.16491063	3.17795E-05	0.001308276	moderately acetylated
SETD5	5.746566224	3.07121154	6.930157326	3.02428E-07	0.005720795	moderately acetylated
C2orf68	7.077518265	0.953825297	2.151004016	8.97088E-06	0.000492958	moderately acetylated
MBD2	2.193770145	1.957102486	4.409814889	5.86168E-05	0.003010837	moderately acetylated
VAMP2	16.2776817	1.922827908	4.327483981	6.47811E-05	0.005742198	moderately acetylated
MSI1	2.734986317	2.328082129	5.225751783	1.6841E-06	0.01977329	moderately acetylated
LOC9078	1.31500504	1.418357107	3.183217527	0.000123404	0.044343049	moderately acetylated
FLJ3130	7.454710095	3.220427792	7.226634477	6.23841E-06	0.001018691	moderately acetylated
SMARC	10.34017223	3.13387973	7.023741573	1.42178E-06	0.001510876	moderately acetylated
INO80B	1.043354124	3.912097517	8.765747137	0.001005674	0.025947145	moderately acetylated
MYH3	1.054130305	0.948649323	2.125441108	0.004032513	0.024659305	moderately acetylated
SCAND1	40.70034837	1.076499719	2.401737106	0.000226995	0.002366243	moderately acetylated
CRYBB3	1.046799006	0.955293226	2.129490926	0.012100506	0.044870991	moderately acetylated
SLC24A1	1.402537054	1.607593508	3.582756579	0.000224841	0.007826491	moderately acetylated
LINC002	1	1.173862113	2.611139052	7.2212E-05	0.031759428	moderately acetylated
KCNC1	1.01543608	1.225953621	2.726102216	0.001463091	0.01934349	moderately acetylated
HOXB5	2.613284857	1.742930318	3.873779171	1.41253E-05	0.012068616	moderately acetylated
PHF3	4.762999988	1.582747913	3.515932671	2.44678E-05	0.000811	moderately acetylated
SLC2A4I	6.339728392	2.83731139	6.300329614	1.91786E-06	0.024927924	moderately acetylated
SLX4	1.207759883	0.989243808	2.196324134	0.000860846	0.005229432	moderately acetylated
STX2	5.014490303	1.28609434	2.855337199	0.00015253	0.003647851	moderately acetylated
IMPA2	5.66607435	1.271368445	2.822301973	7.66848E-05	0.001242864	moderately acetylated
CEP350	2.21225297	3.079699024	6.836502333	2.32515E-06	0.001540516	moderately acetylated
VPS53	1.564806945	1.146326254	2.543298541	1.45899E-07	0.006724108	moderately acetylated
AMT	1.459671016	2.357442791	5.229336638	0.005336432	0.049389445	moderately acetylated
RHOV	2.597825014	1.754531507	3.891266502	0.002507103	0.028339724	moderately acetylated
TLN1	4.893188158	1.707286918	3.784427964	2.91416E-05	0.00311871	moderately acetylated
MAGI2-	1.183446115	0.912587927	2.022828912	0.000515085	0.003130297	moderately acetylated
TTF1	5.543307666	1.520252725	3.362020665	0.000107149	0.006130331	moderately acetylated
TRIM65	4.009963653	1.404689479	3.104795922	2.02334E-05	0.000674673	moderately acetylated
ZNF529	1.730537561	1.192708574	2.636207747	8.73227E-07	5.66662E-05	moderately acetylated
VEGFA	3.776463531	3.320992305	7.336437611	8.80272E-06	0.002202229	moderately acetylated

KREMEN	2.408821533	2.583599775	5.703467438	3.38255E-06	0.002765505	moderately acetylated
TCAM1F	1.122344647	0.920999493	2.030563895	0.000123174	0.000652328	moderately acetylated
MIS18BF	4.026998334	1.322555516	2.913299812	2.50279E-05	0.002714163	moderately acetylated
SOX3	1.040765923	0.960830843	2.115343811	1.52161E-05	0.001187905	moderately acetylated
TERF2	5.628184043	1.430644616	3.147109578	7.04265E-06	0.000260449	moderately acetylated
RMI2	20.63444803	1.069779683	2.349833346	0.000244433	0.002165028	moderately acetylated
RAI1	1.556248386	1.02434283	2.247578253	0.00055576	0.007577535	moderately acetylated
CHCHD1	137.4993181	1.877257986	4.11669377	0.001584763	0.020554817	moderately acetylated
DUSP15	2.567668802	1.120335958	2.456222362	0.000471714	0.001141713	moderately acetylated
AARS2	4.733792727	1.164221594	2.550798004	2.82304E-05	0.003853825	moderately acetylated
MIER2	4.422233799	1.951749533	4.274612854	7.31428E-06	0.000419224	moderately acetylated
IQSEC1	1.584998642	1.145787676	2.50726683	4.80092E-05	0.001887204	moderately acetylated
SEC63	10.28582366	1.747059195	3.821998224	9.86419E-07	0.000154829	moderately acetylated
NHLH1	1.016519837	0.983748633	2.149596441	0.001281037	0.014556423	moderately acetylated
GIPC3	1.325863551	1.647073368	3.598409801	5.50669E-05	0.013197826	moderately acetylated
HTR7P1	1.071113877	0.933607548	2.038934085	0.004189552	0.024506878	moderately acetylated
EBF4	1.856396756	2.321712003	5.065481942	4.46808E-06	0.000209584	moderately acetylated
KIAA028	1.701050685	1.94107975	4.234049999	5.87156E-07	5.54319E-05	moderately acetylated
KCNQ1C	1.045396148	1.002738284	2.183467651	7.79294E-08	7.77867E-05	moderately acetylated
ZNF238	1.420964927	1.262364205	2.748484317	1.46263E-05	0.017393804	moderately acetylated
ZNF213	1.689778495	1.094715588	2.382974932	0.001398772	0.030784993	moderately acetylated
ZNF37A	2.403857906	2.592993597	5.640194665	1.88182E-08	9.1065E-06	moderately acetylated
NPB	1	1	2.171212979	0.000417966	0.008475662	moderately acetylated
ANKRD6	1.044481181	0.957413133	2.070960537	0.020216023	0.06003586	moderately acetylated
WIPF3	2.310396401	1.53617641	3.320700877	5.82807E-06	0.04077491	moderately acetylated
FBXO31	3.171785388	1.695989129	3.658190835	0.00056433	0.020989021	moderately acetylated
TAF4	1.832486487	1.590993012	3.42997033	9.65861E-05	0.005581134	moderately acetylated
FAM160I	2.641300327	1.13649163	2.449917068	0.000117233	0.002836624	moderately acetylated
FZD2	2.128532012	1.175172909	2.529354525	2.74666E-05	0.002576625	moderately acetylated
C16orf42	7.060259464	1.121744282	2.414309395	0.000228401	0.005909465	moderately acetylated
NCOA1	1.728684156	0.939206298	2.020332115	0.001383684	0.011340937	moderately acetylated
PBRM1	3.10337026	1.49086967	3.206990697	9.05575E-07	0.039664654	moderately acetylated
SUPT16I	38.47344208	2.507779763	5.390736162	7.57004E-06	0.000582289	moderately acetylated
PNPLA2	19.02863001	1.297364726	2.784865885	0.000302508	0.008663395	moderately acetylated
LOC4407	1.09166534	0.996000227	2.137856834	0.005114131	0.019518423	moderately acetylated
NPTX1	2.768881052	4.077044832	8.746279716	2.23102E-06	0.004140361	moderately acetylated
SIPA1L2	1.953331961	0.98187334	2.104188946	3.66868E-06	7.06565E-05	moderately acetylated
PPP1R14	25.18618301	9.271803789	19.86393252	6.92995E-06	0.0009794	moderately acetylated
GLYATL	1	1	2.141171241	0.000286936	0.007003999	moderately acetylated
HRASLS	1	1	2.141049255	0.035015962	0.088455667	moderately acetylated
RECQL4	6.261042524	1.797630735	3.848064105	2.98379E-07	0.000710264	moderately acetylated
NCLN	15.34090579	1.665690146	3.564197388	3.75782E-05	0.004050384	moderately acetylated
RNF166	5.722300248	1.169269997	2.501093971	3.78364E-05	0.01071115	moderately acetylated
FLJ4598	1.060179883	0.943236158	2.0136264	1.0321E-05	0.000456292	moderately acetylated
CAPNS1	35.93898088	4.393527009	9.378066195	0.000127787	0.012192266	moderately acetylated
NINL	2.153133418	2.213274989	4.714518518	0.000199146	0.011453997	moderately acetylated
FLJ9075	1.340429134	2.984183063	6.344434622	2.92815E-05	0.007708034	moderately acetylated
DENND4	3.665354661	1.594775252	3.387226937	5.77672E-05	0.002535734	moderately acetylated
MAFA	1.215598321	5.259172215	11.16492075	1.69538E-06	0.020970694	moderately acetylated
PRRT2	1.775346253	2.743796797	5.824159273	4.66841E-05	0.005549153	moderately acetylated
FAM115	5.041710474	1.746676558	3.705956025	1.86368E-05	0.002743835	moderately acetylated
CRTC1	1.448212569	1.846445954	3.912953842	1.83745E-05	0.00088759	moderately acetylated
NCKAP5	1.079557079	1.083463343	2.294839698	1.4597E-06	0.008644952	moderately acetylated
NIPBL	2.96932347	2.699271799	5.712442124	3.01837E-05	0.004673738	moderately acetylated
LTBP4	6.28953062	0.969767394	2.049332212	0.000361383	0.00316663	moderately acetylated

LOC1001	1.070258272	1.037110175	2.190371388	0.001054653	0.006622241	moderately acetylated
MRPL41	285.3939904	1.304306349	2.7505393	4.7147E-07	0.000771387	moderately acetylated
C14orf10	2.89874852	1.840737418	3.880255411	8.19186E-06	0.001675456	moderately acetylated
MZT2B	61.42688694	5.415968148	11.40505129	8.59807E-05	0.009201974	moderately acetylated
ZNF324	2.936691273	1.195767258	2.515447567	0.000271153	0.008094391	moderately acetylated
TCF7	2.374824083	1.988158473	4.18124058	2.4993E-07	0.000972025	moderately acetylated
C14orf80	2.384095225	4.076509038	8.569997177	2.2549E-05	0.004241194	moderately acetylated
COL13A	2.928406239	1.855046266	3.897267911	6.53916E-06	0.00123163	moderately acetylated
ECI1	51.36097819	2.906556451	6.102950326	4.18991E-06	0.00170663	moderately acetylated
TPTE2P1	1.063963567	1.004216389	2.104552123	6.47944E-07	0.027535735	moderately acetylated
POU4F1	3.345441731	2.871194606	6.013569128	5.48555E-05	0.002511512	moderately acetylated
RNF44	5.501020615	1.612612005	3.37726836	0.000766599	0.010422758	moderately acetylated
DNAJC5	1	1.132942149	2.372575896	0.000802823	0.006563869	moderately acetylated
BTNL9	2.213550679	1.465888575	3.068883226	0.000180904	0.026684506	moderately acetylated
MLL	2.751487678	2.836693527	5.938455851	1.8414E-06	0.011175237	moderately acetylated
SMAD9	2.320643924	1.013543222	2.115388284	5.24311E-05	0.000625418	moderately acetylated
CSAD	2.063994644	2.999074107	6.252783209	9.31069E-05	0.003936066	moderately acetylated
MLLT1	3.709773438	3.087191096	6.428682904	3.93237E-05	0.003809914	moderately acetylated
LOC914	1.024220623	0.976352142	2.029808867	0.024524485	0.068926112	moderately acetylated
TBX3	2.458693379	1.536335741	3.191716126	4.55317E-05	0.001210955	moderately acetylated
ALMS1	1.389649592	2.258128033	4.687723071	6.60504E-07	0.022976374	moderately acetylated
HES1	13.56378874	6.421209175	13.32068017	4.08138E-06	0.021101205	moderately acetylated
BAZ1A	5.988180152	3.448062796	7.150174355	4.45749E-06	0.009912835	moderately acetylated
MAP2K7	3.119395827	4.26018919	8.823207858	7.57806E-06	0.023095172	moderately acetylated
CHML	2.609371449	1.266675742	2.62281031	0.0001133	0.004024129	moderately acetylated
SMC1A	18.08367765	1.851544393	3.829702756	1.73082E-09	0.006595325	moderately acetylated
BMP7	5.969572455	1.470111763	3.039367899	0.000238542	0.006473934	moderately acetylated
HLX	3.885179351	1.003079745	2.071651506	0.000336947	0.002872293	moderately acetylated
BTBD2	13.9279438	2.110759678	4.35924272	3.1244E-05	0.004371926	moderately acetylated
ZFHX2	1.112137529	1.025848975	2.105785532	0.001197721	0.013701127	moderately acetylated
SRCAP	3.316357612	1.853088245	3.802107218	7.37745E-05	0.00479374	moderately acetylated
STRN4	13.61166636	1.270610802	2.601588112	4.8603E-05	0.000869205	moderately acetylated
C2orf72	1.440032127	1.553938018	3.174845203	0.00098012	0.01988078	moderately acetylated
GNB3	1.851606553	1.788560352	3.652982889	1.70706E-05	0.004594259	moderately acetylated
TSPAN1	2.779683509	1.021011318	2.080928601	8.87527E-06	8.56096E-05	moderately acetylated
SPTAN1	4.970198961	1.184829851	2.407088173	6.82221E-06	0.00051073	moderately acetylated
WDR52	1.1094798	1.085117639	2.204500923	0.002873059	0.020247655	moderately acetylated
MAML3	1.162644662	0.991447292	2.013307291	0.000505693	0.008242859	moderately acetylated
GLT25D	9.973989023	1.063443115	2.154093983	8.17862E-06	0.002422391	moderately acetylated
RBM26	8.097766652	2.016111697	4.083702605	2.07903E-05	0.003878414	moderately acetylated
DPY19L	2.124045474	1.030486436	2.086754773	0.000101297	0.045074498	moderately acetylated
RCCD1	5.482807621	2.07801095	4.206851398	5.18646E-05	0.002033465	moderately acetylated
FRG1B	17.49924072	1.287575565	2.605138384	0.000166049	0.00217441	moderately acetylated
EMD	114.9075057	2.021368769	4.088093706	8.45887E-06	0.005316807	moderately acetylated
ELFN2	1.055321662	1.190254529	2.406395638	3.19164E-06	0.033329324	moderately acetylated
RPUSD1	20.03230274	1.177507603	2.379273575	0.000188365	0.027938048	moderately acetylated
LOC645	1.041046283	1.01180742	2.042916474	0.003470703	0.01891722	moderately acetylated
USP35	1.34607201	1.342448586	2.703445085	0.000386677	0.006696149	moderately acetylated
RBM5	13.05573021	1.400437185	2.808976242	2.47663E-06	7.96157E-05	moderately acetylated
TTC14	3.776345149	1.024760047	2.055404074	2.61998E-05	0.001879918	moderately acetylated
WSB1	12.96673051	1.736970824	3.479760223	1.83853E-05	0.024290079	moderately acetylated
SAFB	12.38940421	1.801082667	3.602342351	1.35331E-05	0.001229039	moderately acetylated
