# Supplementary material for: PCBP1/2 and TDP43 Function as NAT10 Adaptors to Mediate mRNA ac4C Formation in Mammalian Cells
Source: Adv Sci (Weinh). 2024 Nov 18;11(47):2400133. doi: 10.1002/advs.202400133 (PMC11653668; doi:10.1002/advs.202400133)
Supplement: Supplementary file 3 — Supporting Table [file ADVS-11-2400133-s002.pdf]

ADVANCED SCIENCE
Open Access

Supporting Information

for *Adv. Sci.*, DOI 10.1002/advs.202400133

PCBP1/2 and TDP43 Function as NAT10 Adaptors to Mediate mRNA ac^{4}C Formation in Mammalian Cells

Zhi-Yan Jiang, Yu-Ke Wu, Zuo-Qi Deng, Lu Chen, Yi-Min Zhu, Yuan-Song Yu, Hong-Bo Wu and Heng-Yu Fan**

Table S2. ac4C(+) mRNAs in *siTDP43* 293T cells

Gene Syı	FPKM+1_inpı	FPKM+1_IgG	FPKM+1_ac4(	p-value_ac4C ı	p-value_ac4C ı	Cluster
RPPH1	1.959689105	3.369962128	34.61661092	0.002070347	0.002494746	highly acetylated
SCARNA	1	1	11.518739	0.001902636	0.001902636	highly acetylated
SNORD1	1.715351207	1	19.5016669	0.002224517	0.00187432	highly acetylated
PCSK1N	3.48477139	3.769841401	38.53771316	0.000329578	0.000380049	highly acetylated
SCARNA	1.345561542	1	13.40566312	0.000108236	6.37594E-05	highly acetylated
MPND	1.966493373	1	19.00096452	2.03978E-06	1.55234E-06	highly acetylated
VPS37D	1.04456775	1.258672326	10.05010138	0.002988981	0.003313523	highly acetylated
OR51B6	1	1	8.257412701	0.000472558	0.000472558	highly acetylated
HIATL2	2.274788872	3.097402239	17.66210298	0.00023676	0.000310285	highly acetylated
SNORA2	1.397850501	1	10.69573863	0.038217422	0.032822003	highly acetylated
HP07349	3.659551442	3.242458302	27.37783601	2.66753E-05	0.000110079	highly acetylated
BTBD6	3.082561435	3.759560714	22.83859291	0.000468613	0.000553288	highly acetylated
SNORA7	2.123964697	1	14.84191372	0.047312758	0.035655224	highly acetylated
SNORA2	8.423152676	1	55.9528974	0.002713871	0.001423951	highly acetylated
LOC389(	1.267157421	1	8.052041502	0.001425334	0.001140715	highly acetylated
FZD9	1.240416819	1.169948529	7.615939872	9.22906E-05	0.000104392	highly acetylated
FKBP1B	4.358013163	4.033634979	26.60743508	0.000726359	0.000726526	highly acetylated
WFDC3	1.413189693	1	8.586121831	0.008651745	0.006977254	highly acetylated
HPS6	2.588217092	2.223766386	15.58451432	0.001329463	0.001191185	highly acetylated
C19orf6	2.796145581	1.99446101	16.73332891	0.000558224	0.000467012	highly acetylated
VAX2	1.492820276	1.677062039	8.807297535	1.86892E-05	0.000643436	highly acetylated
LOC1002	6.952883255	2.936077915	40.76338948	3.98358E-05	1.58973E-05	highly acetylated
SNORA5	3.164934314	1	18.41712967	0.001403733	0.000712464	highly acetylated
NPPC	2.124688461	1	12.09139817	0.000603562	0.000396551	highly acetylated
PRSS45	1	1	5.666169805	0.00100767	0.00100767	highly acetylated
UNC93B	1.168232203	1	6.494165415	0.00094533	0.000787668	highly acetylated
FAM110	1.154246392	1	6.350952541	0.000580813	0.000506498	highly acetylated
C8ORFK	1.120017533	1	5.9781068	0.000344578	0.000296036	highly acetylated
RCAN1	2.620604297	1.65087913	13.76484876	3.59342E-06	7.6728E-06	highly acetylated
SNORA6	2.882708233	1	14.83921664	0.044279966	0.028311071	highly acetylated
DPH3P1	1.010058018	1	5.175796633	0.001850067	0.00183225	highly acetylated
FAM131(	1.059973643	1	5.413804225	0.008125231	0.00772711	highly acetylated
SENP3	1.294327514	1.0797954	6.540416659	0.003324433	0.002862613	highly acetylated
IGFBP6	1.481814686	1.200805667	7.444001012	0.009218687	0.008112557	highly acetylated
CRLF1	1.698371354	1.453558754	8.52035001	0.001256934	0.00135145	highly acetylated
INSM1	1.130117971	1	5.668052752	2.05073E-06	1.34923E-07	highly acetylated
CHMP4E	2.036401029	28.1705036	68.95284615	5.84994E-05	0.000482269	moderately acetylated
SAP30	1.856914118	13.73131284	43.96265039	6.47907E-07	2.67692E-05	moderately acetylated
TUSC1	1.993859428	9.865410543	29.79505571	0.002159993	0.008050081	moderately acetylated
RILP	1.819650496	8.793015998	23.60348721	1.00878E-05	0.000451516	moderately acetylated
HMX1	1.123775367	4.343218656	13.8469929	0.000403269	0.003105277	moderately acetylated
HOXB2	1.880537899	11.10287669	23.16396541	3.33524E-05	0.000499293	moderately acetylated
BRI3BP	2.435452663	11.03592467	27.10994397	0.000591699	0.003975588	moderately acetylated
CNO	5.892892998	22.85936303	54.63140884	2.03361E-07	0.000781998	moderately acetylated
SDCCAC	4.525208383	18.24000442	41.89849291	8.70053E-06	0.000145279	moderately acetylated
AES	8.445633049	36.65414443	75.87292005	1.62388E-06	5.21259E-05	moderately acetylated
CMTM8	2.932778718	7.679130447	26.23863624	0.000182715	0.001328041	moderately acetylated
LOC936:	3.672173668	14.10673493	31.69951889	1.79607E-05	0.000112181	moderately acetylated
SFT2D3	1.549001631	3.261809843	12.53809688	0.000364066	0.000752728	moderately acetylated
MESDC1	1.578322694	5.633966187	12.16912543	0.000129012	0.001348743	moderately acetylated
ZNF238	1.4547477	3.209052321	11.10249527	6.9132E-05	0.000223216	moderately acetylated
VEGFB	2.379111315	7.46225261	16.50797115	0.002909828	0.014482983	moderately acetylated
STK39	2.329901845	7.432792761	15.28086131	0.00010021	0.001039809	moderately acetylated

CRLS1	2.99528258	8.487231595	19.45165	1.83887E-05	0.000522526	moderately acetylated
C9orf40	3.726249325	11.40676367	23.64441235	6.81266E-06	0.000432653	moderately acetylated
FBLL1	1.185246317	2.720562715	7.475724162	0.000633803	0.002594866	moderately acetylated
KAT8	3.431869176	7.651700547	21.52172681	2.31403E-05	0.001253162	moderately acetylated
CEBPA	1.60974831	3.319305917	9.940957137	0.001121567	0.004411683	moderately acetylated
RCCD1	3.343255489	5.840081843	20.37103549	3.43643E-05	0.000250285	moderately acetylated
CTSF	3.383382363	9.260928574	20.40422919	5.39176E-05	0.00054966	moderately acetylated
MMP24	1.293469933	3.869893285	7.795984214	0.000497874	0.00483169	moderately acetylated
FOXF2	1.995519883	4.745704735	11.91508829	1.834E-06	0.00230193	moderately acetylated
ZNF205	1.178814126	2.385793544	7.00959118	3.65396E-05	0.000167566	moderately acetylated
PUS1	3.039342964	7.836417808	17.98466086	2.73875E-05	0.000244411	moderately acetylated
PEX7	5.118143458	11.29149071	29.75836603	0.00149813	0.005924239	moderately acetylated
KRT10	4.308018352	10.48907078	24.80752411	1.12158E-05	0.000229511	moderately acetylated
CITED4	1.018469207	1.664253062	5.784689071	0.001196728	0.005066239	moderately acetylated
SLC30A5	4.903410488	12.74860833	27.33381618	1.88986E-05	0.000121006	moderately acetylated
DNAJB2	1.208827977	2.097466496	6.5761072	0.000668946	0.002934459	moderately acetylated
CAHM	1.249699659	1.748953079	6.78529404	0.000679499	0.005709211	moderately acetylated
SSBP4	2.028906074	2.360548857	10.76353347	3.24011E-06	0.000217589	moderately acetylated
RBM38	3.527775893	7.138464768	18.05822482	0.000205186	0.000658552	moderately acetylated
D2HGDH	1.160373922	1.301989385	5.784839825	8.81561E-05	0.000209812	moderately acetylated
LOC1001	4.317822625	6.84995732	21.49185706	0.000211984	0.000776696	moderately acetylated
CAPN10	2.193708336	1.922679885	10.85283216	2.48752E-07	3.63232E-06	moderately acetylated
RRAS2	2.145561065	3.77469327	10.54262863	0.000275993	0.002280146	moderately acetylated
TTC19	4.208187433	3.897682525	20.63205505	5.04686E-06	1.35303E-05	moderately acetylated
SLC12A9	1.463028982	1.179720015	7.138070356	0.000887464	0.000744388	moderately acetylated
FAM184A	1.453883418	1.094490984	7.068108738	7.11626E-05	5.76118E-05	moderately acetylated
EMID1	1.190827823	1.78461853	5.780301322	6.93681E-06	5.71154E-05	moderately acetylated
NECAB3	3.000350979	5.6818115	14.5319502	0.003553006	0.009778661	moderately acetylated
SUZ12P	2.397779362	1	11.59960227	0.001844549	0.001073525	moderately acetylated
MAP2K1	7.062697285	15.48175295	34.05975174	1.37819E-05	0.000110057	moderately acetylated
CNKSR3	2.010233862	1	9.649332215	1.52442E-06	2.73508E-07	moderately acetylated
SNORD1	4.819812177	1	22.98822949	0.026825861	0.01374033	moderately acetylated
ASPSCR	4.178817424	8.57226008	19.74684272	0.000389089	0.001369632	moderately acetylated
DNAJC2	1.762097033	3.347655181	8.14631529	5.37876E-06	2.65634E-05	moderately acetylated
LOC2855	1.322446358	1.197491794	6.085709984	0.000215255	0.000291941	moderately acetylated
C11orf85	1.497664087	1	6.888291546	0.004364975	0.003019557	moderately acetylated
DMWD	1.325748554	2.115586908	5.962206993	3.11894E-05	0.001532182	moderately acetylated
ACBD3	7.322306843	16.18447663	32.85833156	0.000174381	0.001898759	moderately acetylated
BRD7	6.556462196	10.09022549	29.38793534	8.79683E-06	1.25793E-05	moderately acetylated
KLRG2	2.061467088	2.652789045	9.217943231	0.003740849	0.010413614	moderately acetylated
EID2B	2.856736436	3.821477924	12.76797951	1.585E-06	2.88087E-05	moderately acetylated
PPP2R5A	2.688308225	4.408244762	12.00650955	0.000524928	0.004739986	moderately acetylated
SLC25A2	3.960941641	1.394970297	17.55648272	0.000121817	5.70263E-05	moderately acetylated
PURA	2.458542116	2.925474272	10.88758245	0.000719709	0.002669735	moderately acetylated
HMX2	1.387975734	1	6.138612797	0.000876251	0.00059573	moderately acetylated
IL18	1.249909285	1	5.503989268	0.000526993	0.000417704	moderately acetylated
LOC3899	1.342677888	1.219423787	5.898857333	0.001884807	0.001750858	moderately acetylated
SGPP2	1.230903247	1	5.367534916	0.002996593	0.002420799	moderately acetylated
APOC1P	1.109007009	1	4.797186182	0.040375467	0.036845307	moderately acetylated
ZCCHC3	4.409640874	3.606274308	19.0430978	4.94046E-06	2.44822E-06	moderately acetylated
CYTH3	1.989688014	3.256408073	8.539772762	4.50422E-05	0.002733299	moderately acetylated
MANEAL	1.902984668	3.104862429	8.090523776	0.000286356	0.000728487	moderately acetylated
PIP5K1B	1.321710122	1	5.606447368	0.000318027	0.000235415	moderately acetylated
ANKRD1	1.300956286	1.150879248	5.513422445	0.002352087	0.002230314	moderately acetylated
MGEA5	5.114495654	8.442967457	21.38970093	0.000178652	0.000717768	moderately acetylated

NXN	2.253437114	3.606855471	9.325205602	1.01743E-05	0.000211785	moderately acetylated
LOC6531	1	1	4.12739727	0.001212922	0.001212922	moderately acetylated
SPHK1	4.157894714	1.970943184	17.12812155	0.000117058	6.34546E-05	moderately acetylated
TMEM13	1.159339474	1	4.709756509	0.004290872	0.003583515	moderately acetylated
CSNK2A	2.236352701	3.791165081	9.084762965	0.000445177	0.005181125	moderately acetylated
SPRY2	2.146302767	1.485458029	8.718428646	6.8726E-06	1.2558E-05	moderately acetylated
TNIP1	4.360347655	5.949801494	17.60086041	1.0088E-05	3.40779E-05	moderately acetylated
MTTP	1.102587853	1	4.441415334	0.000398577	0.000335272	moderately acetylated
KREMEN	1.182742707	1.290242675	4.728801242	0.00014792	0.000850167	moderately acetylated
C13orf15	1.506292247	1.350141289	5.979541638	0.027246829	0.026651048	moderately acetylated
WT1	1.264731441	1	5.018420674	0.00019124	0.000145863	moderately acetylated
RHOV	3.282562856	3.743193737	12.90530596	0.001073689	0.00168049	moderately acetylated
OLIG2	1.063956658	2.080024462	4.170685924	0.006127926	0.044964334	moderately acetylated
SLIT2-IT	1.14161923	1	4.467399229	0.02740601	0.023548667	moderately acetylated
PHF23	10.43471807	17.96338246	40.81688953	1.11121E-05	4.9377E-05	moderately acetylated
LEMD2	2.709695204	3.562483505	10.49724705	0.000563478	0.001472161	moderately acetylated
TFPT	10.64125008	19.2215947	41.04213642	3.45118E-07	0.002545721	moderately acetylated
SOX2	1.29907743	1.323721552	5.007627278	3.43652E-05	3.52161E-05	moderately acetylated
HRK	2.930469347	1	11.29385775	0.000182415	7.79713E-05	moderately acetylated
MBD2	1.292459377	1.978494717	4.962747421	0.000805705	0.002180348	moderately acetylated
SLC25A2	1.723104014	1.259147178	6.592149228	0.000366271	0.000253791	moderately acetylated
ZNF554	1.270990204	1.488172253	4.853557644	7.96862E-06	0.000214998	moderately acetylated
ACAP3	1.225511965	1.596051048	4.638709934	6.21139E-06	1.97503E-05	moderately acetylated
LPCAT1	3.498049754	5.942987017	13.22329444	0.000856552	0.006373795	moderately acetylated
BAMBI	2.371125455	1	8.938680595	0.000277561	0.000131206	moderately acetylated
TXNRD2	8.372600926	14.50212637	31.49019528	0.000135397	0.000769974	moderately acetylated
SNORA6	5.970567104	2.436303958	22.42160608	0.011199577	0.006060344	moderately acetylated
LEPREL1	2.976609951	3.31876207	11.00722704	0.000731023	0.000841385	moderately acetylated
C17orf90	44.70052792	79.24840996	164.598186	2.14538E-05	4.15564E-05	moderately acetylated
MBTD1	1.170659622	1.115052074	4.256964549	0.000166848	0.000178209	moderately acetylated
RIPPLY2	2.135373307	1.299713445	7.763261953	0.002864168	0.001238751	moderately acetylated
LSM14B	1.665094083	1.959506258	6.038567667	4.53968E-06	0.000138007	moderately acetylated
MLYCD	1.503023663	1.962697431	5.42113168	8.8044E-07	0.001303712	moderately acetylated
LOC2836	4.166994984	1.671205101	15.01291067	0.000499791	0.000393018	moderately acetylated
CTCF	3.582114429	4.263891226	12.84934816	1.37357E-05	1.41541E-05	moderately acetylated
C11orf68	3.328205849	1.820122747	11.93350887	0.001481088	0.001030929	moderately acetylated
AGAP3	4.670247997	7.73594207	16.66882264	0.000701782	0.001894247	moderately acetylated
HOOK1	2.340525241	2.439600072	8.345018293	0.00011659	0.000126708	moderately acetylated
HIST1H4	2.003099345	1	7.090947927	0.000906064	0.000368166	moderately acetylated
FOXD3	1.902253312	3.050936209	6.713960169	0.000413936	0.030916445	moderately acetylated
PMS2P3	1.528800241	1	5.375153436	0.000835824	0.000477818	moderately acetylated
NCS1	1.891087196	2.761708759	6.634632616	2.42198E-05	0.0011916	moderately acetylated
ZNRF2	1.251336035	1.177508327	4.383459893	0.015330505	0.014108909	moderately acetylated
SOCS1	1.074552095	1	3.752513039	0.012284191	0.011111378	moderately acetylated
HIST1H3	1.288099619	1	4.496366979	0.002547406	0.001538187	moderately acetylated
MPP6	2.033200337	1.179692731	7.093030591	0.002376271	0.001275481	moderately acetylated
PLCL2	1.681395554	1.480344539	5.849209395	0.000127805	0.000149455	moderately acetylated
ZBED3	1.285712054	1.88128168	4.468034866	0.021987216	0.048183751	moderately acetylated
FBXW4P	1.108929162	1	3.823783266	0.003624683	0.003044982	moderately acetylated
ING2	4.569251848	3.027542089	15.71451223	0.00711574	0.004405419	moderately acetylated
AUH	4.430472593	1	15.22858381	2.48167E-05	6.46742E-06	moderately acetylated
RASL10A	1.051267833	1	3.609364479	0.00080935	0.000731968	moderately acetylated
CRIP1	1.089890908	1	3.732035468	0.006854823	0.005755177	moderately acetylated
TTLL12	8.315386455	13.22593522	28.41670877	0.000230419	0.004586512	moderately acetylated
TMEM74	1.724382319	2.496562423	5.875539288	0.000374478	0.008131953	moderately acetylated

C9orf30	3.564425242	4.122860406	12.10640295	0.001264838	0.006659555	moderately acetylated
C10orf11	1.92126833	1	6.511562482	0.028119448	0.014586242	moderately acetylated
RNF19B	2.225166038	2.275644227	7.523735892	8.23425E-05	0.000249978	moderately acetylated
TCEAL3	4.281669881	2.681715601	14.47014287	0.003251719	0.002245064	moderately acetylated
CCDC3	1.572773009	1.781714169	5.301438378	0.000230022	0.002818439	moderately acetylated
LPHN1	1.284979697	1.20481449	4.326846295	0.000721508	0.000838639	moderately acetylated
LOC4404	1	1	3.34745321	0.005030595	0.005030595	moderately acetylated
SLC6A8	2.222329112	2.199814625	7.401801247	0.000167402	0.00026641	moderately acetylated
SUZ12	2.271235383	2.694965459	7.535278715	3.58034E-05	0.000648601	moderately acetylated
FIZ1	1.140429297	1.698357441	3.780453765	0.000909112	0.013115938	moderately acetylated
C2orf29	3.845227882	4.840982291	12.73557527	0.000104329	0.001619234	moderately acetylated
CHMP3	1.340097064	1.478665366	4.433166198	0.005366405	0.007040323	moderately acetylated
MLLT4	2.258815167	3.222927234	7.443972247	0.000132278	0.000273665	moderately acetylated
MAP2K4	3.077972829	4.554530184	10.13753357	0.000192438	0.001135635	moderately acetylated
SNORD5	22.97755627	1	75.63791762	0.012224733	0.000197149	moderately acetylated
RP9P	2.459371048	3.496268154	8.087284823	0.003946831	0.009046483	moderately acetylated
PKP4	3.673088146	5.440855693	12.06344234	8.4654E-06	3.81487E-05	moderately acetylated
PVRL3	1.383692394	1.327518768	4.536601042	0.000747198	0.000724918	moderately acetylated
NRL	1.059685503	1	3.468102169	0.007858888	0.007202415	moderately acetylated
MYEOV	1.118306293	1	3.641375508	0.0045469	0.003731561	moderately acetylated
SNX12	6.991342938	8.605621488	22.71761971	0.000359481	0.002851812	moderately acetylated
INSL3	1.02895408	1	3.328455432	0.001622106	0.001520463	moderately acetylated
PALM3	1.098121633	1	3.548767058	2.28619E-05	1.14802E-05	moderately acetylated
UBTD2	4.71676622	3.013337782	15.22864465	0.000110574	6.8867E-05	moderately acetylated
SNHG12	33.05404735	39.24312486	106.589805	8.9537E-06	0.002022577	moderately acetylated
HIST1H2	1.957332918	1	6.254290741	0.002743129	0.001064366	moderately acetylated
SULT4A	2.17274817	2.566694326	6.91646419	0.000247967	0.00050823	moderately acetylated
CERK	3.71775522	5.747965276	11.81655623	0.000736317	0.002864969	moderately acetylated
PFN4	1.399551229	1	4.446528641	0.000348167	0.000119648	moderately acetylated
TUSC3	4.553560466	4.796981414	14.4584835	0.00036292	0.001482893	moderately acetylated
NT5M	1.287210688	1.620268093	4.082179693	0.002067347	0.008383123	moderately acetylated
FEZ2	7.337182418	11.06978726	23.17057833	0.002469554	0.006756257	moderately acetylated
LOC1001	1.039647745	1	3.280724404	0.018683481	0.017582458	moderately acetylated
AHSA2	1.647670208	1.463604839	5.194975064	0.001798871	0.001748817	moderately acetylated
ZCCHC2	1.982652556	1.209770705	6.244875959	0.001870483	0.001002321	moderately acetylated
SNORA7	1.510937423	1	4.748071853	0.018928898	0.005249151	moderately acetylated
TRIM65	4.838043252	3.781453488	15.07223246	3.2679E-05	0.000236125	moderately acetylated
DKFZp77	1.268885479	1	3.951986372	0.000225119	0.000108839	moderately acetylated
EXOSC6	7.088790321	9.164264848	22.07446971	9.36127E-05	0.002569322	moderately acetylated
CLK2	6.847070744	2.745844778	21.303955	0.025940188	0.012214233	moderately acetylated
ACADS	8.952791608	7.89683026	27.85348646	8.90953E-05	0.000109806	moderately acetylated
RGS7	1.15792665	1	3.599144451	9.34224E-07	8.89124E-08	moderately acetylated
SNORA6	3.951677173	1	12.25965452	0.006681285	0.001965392	moderately acetylated
MAPK9	2.718993346	2.819979843	8.416444886	0.000472581	0.000587039	moderately acetylated
EFNB1	2.035265336	3.085559683	6.296198759	0.001266094	0.004151499	moderately acetylated
QKI	2.675923218	2.911921999	8.27478508	2.95118E-05	5.34132E-05	moderately acetylated
NUP153	3.845968262	2.278954417	11.88067903	8.43142E-05	5.80852E-05	moderately acetylated
HEXDC	1.885848403	1	5.812967056	1.26051E-06	3.00908E-07	moderately acetylated
NPFF	1.8155412	1	5.593569375	0.038794576	0.018438987	moderately acetylated
HOXB4	1.436903982	1.097889684	4.412316139	0.001062447	0.000698481	moderately acetylated
GPR64	1.368124566	1	4.195908108	3.97001E-07	1.31335E-07	moderately acetylated
SMPD1	1.605432025	1	4.890366571	0.000450867	5.03666E-05	moderately acetylated
STXBP6	1.750727721	1.897224004	5.317597684	0.018922633	0.034900742	moderately acetylated
MAFG-A	1.333397536	1.841231182	4.03296316	0.000403044	0.002494586	moderately acetylated
HOXC6	1.368502309	1.086674832	4.122378023	0.001666187	0.001179997	moderately acetylated

ZNF362	2.763263749	1	8.290105388	3.32328E-05	1.87964E-07	moderately acetylated
C10orf47	1.226481575	1.305199436	3.67099338	0.001333616	0.003146221	moderately acetylated
OGFRL1	1.620015327	1.228746813	4.8418637	0.00184611	0.001512793	moderately acetylated
LRFN3	1.062284054	1	3.174025044	0.007340727	0.006423602	moderately acetylated
PSMG4	33.73841683	47.7788632	100.7276123	3.9842E-05	0.000408266	moderately acetylated
CCM2	6.311802638	7.568455156	18.83463186	0.000104566	0.000201967	moderately acetylated
E2F4	11.97181784	17.15952216	35.66748175	0.001527844	0.00494318	moderately acetylated
SIAH2	1.832527345	1.22403293	5.457296189	0.00022421	9.71156E-05	moderately acetylated
LRCH2	1.951771609	1	5.786372803	7.74347E-05	6.17868E-06	moderately acetylated
CHPF	2.81087505	3.830976497	8.327155799	0.000170293	0.001587076	moderately acetylated
SLC30A1	1.571856132	1	4.65502365	0.004135425	0.002082883	moderately acetylated
ZNF217	1.942829311	1	5.725157873	0.000535617	0.000209249	moderately acetylated
ZNRF1	1.051370211	1.138439747	3.097420285	0.002034648	0.002771023	moderately acetylated
HNRNPA	19.4255434	27.45976146	57.19123568	8.16147E-05	0.000207571	moderately acetylated
CCDC92	2.36329748	1	6.948763652	0.000131665	3.76359E-05	moderately acetylated
ATP6V1	1.204448378	1	3.539331853	0.0011255	0.000782123	moderately acetylated
SLC9A3I	2.43716318	2.14478146	7.151185689	0.000771953	0.000905936	moderately acetylated
NFE2	1.192670311	1	3.487895708	3.7481E-06	2.27751E-06	moderately acetylated
CSPG5	1.778225238	1.312029763	5.195575747	0.000196531	0.000110051	moderately acetylated
USP7	3.80866959	4.54232977	11.11636509	0.000130946	0.000693622	moderately acetylated
STK17A	2.417897457	2.062000535	7.05118265	0.000912878	0.001216311	moderately acetylated
PAOX	1.313570913	1	3.828048583	0.002758653	0.001761426	moderately acetylated
TRAPPC	1.672652026	1.572312591	4.87156459	0.014454281	0.013705659	moderately acetylated
LOC439	1.845660466	1.086097769	5.368153113	0.000539624	0.000145499	moderately acetylated
CD74	1.014184263	1	2.946391905	0.001265396	0.00122236	moderately acetylated
DUSP15	1.139248861	1	3.302972331	0.031564056	0.025974825	moderately acetylated
FAM171	2.835067849	1.976943121	8.212920554	0.000348403	0.000413406	moderately acetylated
LOC284	1.053643559	1.510478372	3.048632246	0.001010421	0.013566774	moderately acetylated
ROCK2	1.219678577	1.665829751	3.528734761	0.002796789	0.007637992	moderately acetylated
FTH1P3	1.584080128	1.440461779	4.581948776	0.000931468	0.001213788	moderately acetylated
SRSF9	12.5453195	16.5522458	36.23392087	0.000294443	0.002242011	moderately acetylated
PPP4R2	6.119412524	7.17471215	17.65225511	0.002280804	0.014494117	moderately acetylated
C9orf129	1.32429957	1.336184184	3.815921436	0.007934787	0.014849719	moderately acetylated
ATF5	1.265398656	1.787262724	3.633823309	0.000109908	0.001518641	moderately acetylated
CBX3P2	1.310757058	1	3.762176979	0.005967541	0.003211428	moderately acetylated
FZD8	1.084269339	1.26377937	3.098611588	0.002308497	0.004591463	moderately acetylated
APOBEC	1.068628687	1	3.047052586	0.0110151	0.009681057	moderately acetylated
LRP11	2.805088201	2.130508304	7.993884639	0.001652067	0.00109439	moderately acetylated
FBXO2	1.245614823	1	3.543894407	0.007972213	0.005453405	moderately acetylated
PPP3CB	3.166422094	1.372677001	9.005955721	7.42772E-05	2.55292E-05	moderately acetylated
UBE3A	3.408567283	4.143935139	9.626914415	0.001506581	0.002695775	moderately acetylated
PPP1R14	3.020678054	3.383149277	8.530615728	0.00158103	0.003200595	moderately acetylated
C6orf120	6.198253036	4.465841506	17.44962884	3.18207E-05	0.000231516	moderately acetylated
LONRF1	2.074523185	1.888179332	5.837860185	0.000454413	0.001079302	moderately acetylated
ZC3H18	2.381754863	3.10810022	6.69231526	0.000765321	0.003228488	moderately acetylated
CIB3	1	1	2.809208665	0.008661824	0.008661824	moderately acetylated
VWA1	1.103665801	1.516703505	3.096659795	0.000180917	0.001105438	moderately acetylated
NPY	4.562616016	1	12.79510898	0.005223201	0.001213999	moderately acetylated
TMEM65	1.889028352	1.808226535	5.272813302	1.91554E-05	3.23622E-05	moderately acetylated
PWWP2	1.478157903	1.615488889	4.121959455	0.001406917	0.006766377	moderately acetylated
UBE2H	2.528080978	2.106271924	7.026198744	0.000667258	0.000536947	moderately acetylated
DIAPH2	1.412906386	1.116425348	3.920046749	0.000227525	0.000177715	moderately acetylated
CD68	2.16254281	1.428900275	5.994241998	0.001522542	0.00200364	moderately acetylated
MED26	1.236847506	1.138938951	3.416099772	0.000255516	0.000479399	moderately acetylated
DYRK1A	1.310927098	1.228481946	3.617044754	7.0822E-05	0.000431629	moderately acetylated

IRF2BP1	1.733660214	1.72495235	4.750574457	0.01100449	0.010994694	moderately acetylated
C11orf91	1.080278527	1	2.958970499	0.001564486	0.001333532	moderately acetylated
E2F3	4.148687229	3.411963215	11.35602364	4.72613E-05	0.000134691	moderately acetylated
CNNM2	1.227254179	1.191759996	3.359089559	0.002708405	0.003353981	moderately acetylated
LINC001	1.49020995	1	4.077493517	0.000790238	2.80005E-06	moderately acetylated
C14orf28	2.071229211	2.423784375	5.665931473	0.000779757	0.002362986	moderately acetylated
CHGA	1.047623851	1	2.863914466	0.000134503	0.000112725	moderately acetylated
CHPT1	9.535132922	3.864198149	26.05339113	0.001723276	0.000542293	moderately acetylated
CWC25	7.936595856	9.107264824	21.66548973	0.000605263	0.001206638	moderately acetylated
DONSOI	5.553303292	3.618551551	15.14809436	0.000643034	0.000392815	moderately acetylated
RHOQ	2.41334366	1.883657616	6.582219174	2.05168E-06	7.52642E-05	moderately acetylated
SH3BP5I	2.058561577	1.123503458	5.61045072	0.000128567	4.80499E-05	moderately acetylated
MTCP1N	25.21198206	22.4123882	68.33957184	0.000564061	0.001916246	moderately acetylated
GXYLT1	1.981711347	1.91266137	5.371480749	0.004282934	0.004847267	moderately acetylated
C20orf16	1.089509624	1	2.948654975	0.000209223	0.000142348	moderately acetylated
ALDH5A	2.102301452	2.352168504	5.686339329	0.00090558	0.00323933	moderately acetylated
DIRC2	1.896745936	1.059637317	5.128435373	0.002206664	0.000902812	moderately acetylated
IRX4	1.03869235	1	2.807008621	0.013030156	0.011925241	moderately acetylated
ZCWPW	1.275125369	1	3.439546577	0.036024903	0.024878923	moderately acetylated
LOC5414	1.28612634	1	3.466707085	3.97044E-05	1.16607E-05	moderately acetylated
TMEM17	1.332197596	1.026451903	3.588131962	0.004183571	0.002567501	moderately acetylated
CCDC64	1.418874322	1	3.820018797	0.000228107	8.87104E-05	moderately acetylated
CHST8	1.168169838	1.066935222	3.141359442	0.000827034	0.00075006	moderately acetylated
OVGP1	1.629209942	1	4.375205811	8.11652E-06	2.05204E-06	moderately acetylated
ZNF497	1.191804906	1	3.199110331	0.000375023	0.000253116	moderately acetylated
IRX5	1.061502999	1	2.844969135	9.6381E-05	7.41228E-05	moderately acetylated
C21orf2	4.070641244	3.444975726	10.90735526	1.73048E-05	0.001231248	moderately acetylated
CRTC2	4.441997757	5.868300314	11.86649956	0.001233076	0.002832243	moderately acetylated
B4GALT	2.424512525	2.983544437	6.429909623	0.000623813	0.002299213	moderately acetylated
FLJ90757	1.319861037	1.220520883	3.494258513	0.001668297	0.002204631	moderately acetylated
SAFB2	3.186587949	3.105388702	8.403684738	0.000284704	0.001811655	moderately acetylated
GPR153	1.269715994	1.146258986	3.345569236	0.000635111	0.000997996	moderately acetylated
C10orf54	1.219838314	1	3.211154884	0.000113478	6.19064E-05	moderately acetylated
EFCAB4	3.819973217	2.632237123	10.04287254	0.004907836	0.005568565	moderately acetylated
SH2D3A	1.751205811	1	4.591013648	0.000468014	0.000119838	moderately acetylated
KLF2	1.07547566	1.235267767	2.819472684	0.009613707	0.022782972	moderately acetylated
NIM1	1.620590459	1	4.237435476	3.32234E-05	4.02062E-06	moderately acetylated
SNORD3	8.576769219	1	22.30928889	0.02926925	0.002700248	moderately acetylated
PSCA	1.172706302	1.172266986	3.039824759	0.016978206	0.019657792	moderately acetylated
RP9	12.24849846	8.041986969	31.73246159	1.21909E-05	9.30229E-05	moderately acetylated
DLEU2L	1.12534269	1	2.911154967	0.026300735	0.020929997	moderately acetylated
ASAP2	1.602251441	1	4.12314088	0.002904292	0.001222758	moderately acetylated
RNF166	2.591323708	2.325478552	6.666282642	0.004886463	0.007885938	moderately acetylated
LOH12C	1.839484748	1	4.725269302	0.001396763	0.000518775	moderately acetylated
DUSP22	6.136151112	1.814433856	15.70542951	0.000218109	4.79932E-05	moderately acetylated
CLMP	1.114520205	1	2.851196654	0.001183535	0.000915288	moderately acetylated
LOC1444	3.547605056	1	9.074512612	0.027619951	0.004896083	moderately acetylated
CCNJL	1.158460718	1	2.962933101	7.10051E-06	2.00763E-06	moderately acetylated
LOC1005	3.132116294	1.584484553	8.007383254	0.000412924	0.000149495	moderately acetylated
MTSS1L	1.096466324	1.291249434	2.800839113	0.006434207	0.010060063	moderately acetylated
CEP55	4.040869901	4.816891685	10.31994742	0.00019107	0.001802483	moderately acetylated
SEPP1	3.255019892	2.512217692	8.295839949	0.002404063	0.002870224	moderately acetylated
C1orf133	4.015808282	1.491235002	10.22971615	0.02695767	0.008616072	moderately acetylated
STYK1	2.060027611	1	5.245368966	0.001417742	0.000417718	moderately acetylated
NMB	18.8077867	23.16432035	47.7649612	0.000404672	0.00634522	moderately acetylated

NOG	1.841531692	1.933366956	4.667674012	0.001844895	0.010693408	moderately acetylated
KLF3	1.929323778	1.144824317	4.86152076	0.001352353	0.00054604	moderately acetylated
CTDP1	1.073106536	1	2.68684681	0.003156732	0.002569572	moderately acetylated
YAP1	1.766856717	2.059536666	4.418122591	0.002253496	0.007470025	moderately acetylated
UAP1L1	1.741408057	1	4.353872024	0.003476175	0.001254867	moderately acetylated
MMP17	1.167852863	1	2.91431725	0.000443292	0.000172645	moderately acetylated
LOC1005	1.288582256	1	3.198495353	0.013425976	0.008092129	moderately acetylated
SREBF1	1.172309672	1	2.908208601	0.000194474	0.000125756	moderately acetylated
LRP10	2.704979149	1	6.704955075	0.00049347	8.01655E-05	moderately acetylated
LOC1001	1.580691819	1.693895249	3.912260586	0.000630698	0.014479175	moderately acetylated
USP12	3.018550607	1.842851048	7.468361746	9.08087E-06	9.65673E-05	moderately acetylated
TIMP3	2.455489551	2.481212727	6.068192221	2.02949E-05	0.000678379	moderately acetylated
SNTB2	1.730009347	1.065127407	4.273817175	0.001081424	0.000450656	moderately acetylated
GGA2	6.374274399	5.507350609	15.74537231	8.12444E-06	0.000308523	moderately acetylated
CHML	2.139333803	2.173314958	5.25707878	0.00257696	0.003115947	moderately acetylated
FAM150I	1.097956575	1	2.696591946	0.036731627	0.030325331	moderately acetylated
CRK	9.403422877	10.24629344	23.09107826	5.41137E-05	0.000266036	moderately acetylated
HOXA3	1.528535279	1.232496285	3.748342079	0.001494249	0.0008309	moderately acetylated
TEAD3	1.378610794	1	3.371434487	0.001479185	0.000710667	moderately acetylated
ITPKA	1.211847359	1	2.962516149	0.001176001	0.000613025	moderately acetylated
PLEKHH	1.690506282	1	4.130729623	0.009871819	0.004044044	moderately acetylated
KCNS3	2.461367685	1	5.997219669	0.00083533	0.000149887	moderately acetylated
CRIPAK	1.100328156	1	2.68068787	0.017898824	0.013949656	moderately acetylated
FLVCR1-	3.54548189	1	8.63077008	0.00049822	0.000101001	moderately acetylated
AGFG1	1.982374323	2.129348473	4.823874561	0.000398761	0.002020018	moderately acetylated
ERO1L	2.98345233	3.452885071	7.255879643	0.004302389	0.022331616	moderately acetylated
DNAJC2	1.850384251	1.077405536	4.49979775	0.00789716	0.003044216	moderately acetylated
NKX6-1	1.203941432	1	2.927286944	0.001535338	0.000776131	moderately acetylated
SPATA2	4.787796543	1	11.61751641	0.001397637	0.000169596	moderately acetylated
JAG2	1.942085713	1.600494972	4.698968566	0.00028758	6.51457E-05	moderately acetylated
FAM168I	6.484180512	6.547752017	15.67788794	0.000580506	0.001694621	moderately acetylated
RNF103	1.872850855	1	4.527043711	0.000108698	3.08443E-05	moderately acetylated
FKBP11	1.814565263	1.46313408	4.38362723	0.034479467	0.025028744	moderately acetylated
TMEM21	3.27709166	3.586630339	7.91390392	0.003684152	0.007502849	moderately acetylated
HECTD1	3.131259717	1.739324583	7.548783435	2.076E-05	3.0791E-05	moderately acetylated
SNORA3	1	1	2.405313295	0.003974376	0.003974376	moderately acetylated
MOB2	5.0597709	5.139845663	12.16059844	0.005725456	0.008603717	moderately acetylated
IGDCC3	1.751879723	1.906655781	4.195255377	0.001624856	0.002507955	moderately acetylated
SPNS1	5.048007282	3.735229908	12.06600025	0.004783346	0.003626278	moderately acetylated
SRSF12	4.720182166	2.918750496	11.24060415	4.96147E-05	1.95955E-05	moderately acetylated
RDM1	10.56297986	5.900854183	25.14514955	7.71571E-05	0.000225854	moderately acetylated
SMAD4	2.105859694	1.743088187	5.006155253	0.001841641	0.001207165	moderately acetylated
ESRRA	9.300228492	4.447016894	22.04373846	0.001944874	0.000540836	moderately acetylated
SPIRE1	2.133065888	2.030655569	5.050723359	7.95252E-06	0.0001516	moderately acetylated
GBAP1	2.488570856	1	5.892354239	0.000562417	0.000119089	moderately acetylated
HIST1H4	1.335116838	1	3.148202051	0.000947835	0.000175014	moderately acetylated
PILRA	1.099484017	1	2.589531379	0.030525965	0.024744735	moderately acetylated
FAM116	2.029686522	1.476250495	4.761727823	0.007054243	0.003833156	moderately acetylated
COL23A	1.120188528	1	2.6240652	0.002582878	0.001902296	moderately acetylated
RBM26	3.901378318	1.87769625	9.125786231	0.018699086	0.006262349	moderately acetylated
SLC25A2	2.993985771	3.289634929	6.989182576	0.004132941	0.00673438	moderately acetylated
REM2	1.70453505	1	3.977029553	0.001232438	0.000344694	moderately acetylated
STOX2	1.311434689	1	3.051695682	0.002669692	0.001357455	moderately acetylated
GPR33	1	1	2.325017874	0.013337064	0.013337064	moderately acetylated
RCC2	7.462956846	8.286269362	17.33262466	7.65355E-05	0.000289435	moderately acetylated

FAM122.	2.405243791	1.273478855	5.579847045	3.3146E-06	2.76982E-05	moderately acetylated
FADS3	2.502703007	2.840885462	5.802306597	9.47111E-05	0.001162812	moderately acetylated
PRKCD	4.830863627	4.471589186	11.19396388	0.00029337	0.000554753	moderately acetylated
LOC729:	1.357986339	1	3.145739779	0.003194971	0.001277974	moderately acetylated
NPTX2	1.39033288	1.142999096	3.219800149	0.001378754	0.001474346	moderately acetylated
EBF4	1.065857177	1	2.460218633	0.001577114	0.00120052	moderately acetylated
FGF8	1.009677445	1	2.328330058	0.035568008	0.034763713	moderately acetylated
RADIL	1.100997579	1.107081199	2.53838513	0.004265813	0.004451561	moderately acetylated
KIAA066	4.292341358	1	9.889922689	0.00047167	7.0695E-05	moderately acetylated
IFI27L1	19.36986879	15.9413623	44.597627	0.001935197	0.00231925	moderately acetylated
IL17D	1.280499128	1.104233355	2.946328301	0.007698683	0.005178038	moderately acetylated
UBA2	20.73241622	19.90042911	47.67750217	0.002231102	0.002410124	moderately acetylated
FAM109.	1.710837743	1.94314928	3.928985947	4.59903E-05	4.73134E-05	moderately acetylated
ZNF711	3.931959984	2.246109167	9.02699362	0.000124049	0.000189212	moderately acetylated
KIAA094	2.202467268	2.502987008	5.041091673	0.001565369	0.002607875	moderately acetylated
SUV420I	2.165104987	1.645517269	4.951532136	0.000126912	0.00086638	moderately acetylated
LSR	4.198689184	3.604352712	9.596810369	0.000117695	0.000325698	moderately acetylated
ZNF837	1.063000989	1	2.426544132	0.023902746	0.020535341	moderately acetylated
CDS1	3.929689354	2.493642134	8.959595451	0.001277125	0.000514477	moderately acetylated
WNT11	2.727561845	1.990700405	6.199313023	0.016292863	0.014606667	moderately acetylated
CAMKK	4.188774127	4.34132103	9.518233971	5.52222E-05	0.000674405	moderately acetylated
FZR1	3.202951944	2.355803115	7.277577687	0.002031604	0.000705639	moderately acetylated
LOC1006	3.196352741	1.409813255	7.25194283	0.005398652	0.001731233	moderately acetylated
FCER1G	1.045962498	1	2.370342087	0.000288065	0.000175014	moderately acetylated
ULK1	1.650399258	1.345309407	3.736822856	6.71666E-05	0.000249396	moderately acetylated
MBL1P	1.050232612	1	2.374819119	0.000554576	0.000354127	moderately acetylated
MT1X	27.47186669	6.053332083	62.10106203	0.004832248	0.000749977	moderately acetylated
TBCD	3.585637776	1.632974071	8.105381182	0.002820089	0.000408874	moderately acetylated
LOC100:	1.013815705	1	2.289999496	0.000123674	0.000117038	moderately acetylated
LOC100:	1.216452314	1	2.743260084	6.49388E-05	3.46186E-05	moderately acetylated
LOC729:	1.270231107	1	2.863161854	0.006860274	0.003530523	moderately acetylated
VSTM2L	1.097047778	1	2.469167789	0.019194485	0.015301096	moderately acetylated
MRC2	1.628033852	1.033212571	3.663307943	0.001522433	0.000565903	moderately acetylated
KCNE1L	1	1	2.246501068	0.005841814	0.005841814	moderately acetylated
KLK8	5.175194392	5.431431142	11.61600137	0.001503288	0.014093163	moderately acetylated
TMEM1!	2.212046759	1	4.958377344	0.043831787	0.010805306	moderately acetylated
KLF15	1.04797595	1	2.348900177	0.011616447	0.010231725	moderately acetylated
ZNF333	2.896218849	1	6.477234045	0.000507412	6.51569E-05	moderately acetylated
TNK2	1.19392137	1	2.669640109	0.00827722	0.0047992	moderately acetylated
BRF1	2.292272187	1.405816005	5.124102943	7.86159E-05	3.08295E-05	moderately acetylated
NAA38	6.778113735	6.510027109	15.12867314	4.22576E-05	0.00017209	moderately acetylated
SHB	1.309971763	1.168111321	2.917456758	0.000831536	0.00074925	moderately acetylated
ZHX1-C8	1.681065302	1.144853182	3.732113744	0.000911156	0.000641553	moderately acetylated
GPR157	1.590085967	1	3.529317176	0.00433225	0.001449556	moderately acetylated
MAP4K4	2.589865267	2.38270973	5.741846888	0.000372848	0.001003377	moderately acetylated
TNIP2	3.497501571	2.120607186	7.752273914	6.99016E-05	2.81151E-05	moderately acetylated
COL6A2	1.278905911	1.091667309	2.834039658	0.001289171	0.00112773	moderately acetylated
GPR135	1.104476295	1	2.445070396	0.001025696	0.000245552	moderately acetylated
UPF3B	16.4627601	15.72844696	36.41031979	0.000786978	0.000765304	moderately acetylated
LPAR3	2.452235358	1.231272199	5.413837165	0.005830436	0.00211089	moderately acetylated
PPRC1	3.417006646	1.145356519	7.541499684	0.001340565	0.000173687	moderately acetylated
SCARF2	1.048745758	1.111994301	2.313555494	0.004711284	0.006255949	moderately acetylated
RILPL2	18.89639057	16.83103087	41.66331598	0.001643407	0.000994014	moderately acetylated
LRRC3	1.330248576	1.079445001	2.932918996	8.89704E-06	3.99681E-05	moderately acetylated
SLC25A:	3.106369776	2.542966035	6.843314876	0.002813996	0.001743152	moderately acetylated

TEF	1.50898424	1	3.32186777	0.007164813	0.002379431	moderately acetylated
C3orf39	6.477550292	5.844032102	14.22368189	0.000115378	0.002394888	moderately acetylated
GLI1	1.08412673	1.05361478	2.373978644	0.005192561	0.005030857	moderately acetylated
HOXA5	28.08627598	22.91208196	61.42221411	2.44152E-05	0.001047669	moderately acetylated
CIR1	9.931760343	10.22150135	21.65528206	0.000249629	0.000537073	moderately acetylated
TMEM18	2.816594983	1	6.139915862	0.011512251	0.002288903	moderately acetylated
NFKBIL1	1.534383084	1.446870298	3.344079259	0.000529029	0.000932587	moderately acetylated
C4orf47	1.697193464	1	3.698122873	0.006075267	0.000672428	moderately acetylated
CUEDC1	1.023259272	1	2.228471497	0.000594196	0.000542073	moderately acetylated
SOX9	1.122725061	1	2.441966585	0.014863465	0.010473137	moderately acetylated
EPHB2	1.466174572	1.045428386	3.188763205	1.17069E-05	4.69103E-06	moderately acetylated
UBE2Q2	1.692077588	1.764431026	3.675824929	0.008845299	0.008816325	moderately acetylated
KLHDC1	2.443117542	2.422993676	5.306785262	3.27227E-06	0.000449039	moderately acetylated
ARHGAI	2.213673844	1.278950644	4.80174916	0.000215577	0.000116563	moderately acetylated
NOL4	1.449609344	1.04784573	3.144262331	0.008900342	0.003570585	moderately acetylated
EPHB1	1.332896164	1.125554284	2.887691717	0.005303607	0.003769281	moderately acetylated
SLC35G5	1	1	2.162442694	0.000265118	0.000265118	moderately acetylated
FLI1	1.265609039	1	2.733292427	0.008029638	0.0043508	moderately acetylated
KCTD1	1.617953005	1	3.490239177	0.003960713	0.001358491	moderately acetylated
SMAD6	1.169174347	1.122887725	2.520173304	0.002561372	0.003309643	moderately acetylated
GAS1	1.489821281	1.138963226	3.210474228	0.009193669	0.004148226	moderately acetylated
ANKRD3	1.227992526	1.22511547	2.644326865	0.000250128	0.000796045	moderately acetylated
ADA	13.49967257	7.701819407	28.99617476	7.89418E-06	5.2164E-05	moderately acetylated
MSC	1.239123755	1	2.655790106	0.001097465	0.000504677	moderately acetylated
NME9	1.192247128	1	2.555240092	0.014382616	0.008748885	moderately acetylated
MFI2	1.18064033	1.040319741	2.529806787	0.002170209	0.001585496	moderately acetylated
TLE2	1.210568564	1.210335209	2.590664177	8.68058E-05	7.09264E-05	moderately acetylated
ITGB5	2.405262862	2.342683633	5.143306611	0.000237774	0.003480752	moderately acetylated
STARD7	26.29432478	16.31373234	56.21603484	0.000196567	9.91561E-05	moderately acetylated
NAF1	4.348597727	4.120429503	9.28784898	0.001425568	0.010044427	moderately acetylated
PPP1R3E	1.48190525	1.042982113	3.160244183	0.012288182	0.005052543	moderately acetylated
ACSS1	2.512125924	1.247524537	5.356258623	0.000874751	0.000335894	moderately acetylated
COMMD	15.24443411	10.8468476	32.48219391	0.00244292	0.005856881	moderately acetylated
COBL	1.522796555	1.377452113	3.242926587	4.60568E-05	0.000335694	moderately acetylated
GYLTL1	2.186962415	1.623806749	4.652630898	0.01069837	0.005455361	moderately acetylated
SWAP70	4.632521434	3.133075965	9.852558252	0.000209306	6.5007E-05	moderately acetylated
FLJ2685(	1.096339025	1	2.331607422	6.58583E-05	6.09063E-06	moderately acetylated
MFI2-AS	1.236989	1	2.626107797	0.004908736	0.00272714	moderately acetylated
CNOT6L	1.872270771	1.072935109	3.973562713	0.000145288	3.99344E-05	moderately acetylated
FERMT1	1.010547015	1	2.13697675	0.000286175	0.000269285	moderately acetylated
APOBEC	1.341716236	1	2.826612958	0.036361978	0.017884112	moderately acetylated
LSM11	1.518549823	1.472943379	3.192629939	0.003553842	0.006847718	moderately acetylated
ANKLE2	5.293707234	2.906298983	11.11011569	0.001121918	0.000494663	moderately acetylated
TRIB3	8.322621163	4.289753873	17.42032369	4.75792E-05	0.000312556	moderately acetylated
JMY	1.552986451	1.151495803	3.246780242	0.004694156	0.002530023	moderately acetylated
HIST1H1	1.193150574	1	2.493557966	0.002315955	0.000498152	moderately acetylated
LOC1003	2.995615849	1.830338167	6.256207775	0.019246755	0.008975139	moderately acetylated
LENG1	15.45359655	8.503128738	32.24569398	0.001313432	0.001618312	moderately acetylated
RILPL1	2.202608174	1.600984551	4.595039455	2.51938E-05	0.000853775	moderately acetylated
ARID4A	2.304850712	1.747817274	4.798117672	0.0055564	0.002773489	moderately acetylated
CANT1	9.21022186	8.167904418	19.1448439	4.56161E-05	2.40196E-05	moderately acetylated
TMEM15	1.13413556	1	2.355676872	0.040083003	0.028549407	moderately acetylated
SLC22A1	1.459127286	1.264049748	3.028529844	0.035763642	0.028795184	moderately acetylated
LOC6456	5.336563949	1.952010677	11.07035576	0.000139988	3.39544E-06	moderately acetylated
SCARA5	1.100676784	1	2.277188477	0.003617843	0.002597108	moderately acetylated

SGPP1	3.236080856	2.197306151	6.692433837	2.42808E-05	0.000188923	moderately acetylated
USP6NL	3.22471356	2.75415031	6.668333621	0.000379117	0.002336686	moderately acetylated
BSPRY	2.0749244	1.249985637	4.277482553	0.014485882	0.005248853	moderately acetylated
LOC1005	1.254337142	1	2.581236304	0.003329737	0.001633532	moderately acetylated
TRAPPC	38.44612815	32.12450453	79.07890359	0.005604939	0.003183142	moderately acetylated
RAB4A	33.67600442	23.75657504	69.23205848	4.12641E-05	4.68419E-05	moderately acetylated
SFN	1.504568798	1.298850192	3.091119463	0.001589836	0.001865218	moderately acetylated
FBXO46	1.387468758	1.351681932	2.849628732	0.001893948	0.004252243	moderately acetylated
PAQR3	5.134481357	3.663633983	10.51745147	0.003740396	0.001705709	moderately acetylated
SPSB4	2.012647591	1.080522322	4.121421405	0.023024334	0.00659654	moderately acetylated
ZNRF2P	1.023917288	1	2.09531526	0.00166623	0.001511402	moderately acetylated
CABIN1	2.720456979	1.402151824	5.564876067	5.70336E-05	4.02241E-06	moderately acetylated
KBTBD2	5.371188573	3.611134172	10.97567742	0.000733052	0.000232427	moderately acetylated
ZNF783	1.450502898	1.275725617	2.963226465	0.004250093	0.001866152	moderately acetylated
SNX7	16.51196103	6.831310132	33.71412011	0.004124841	0.000573314	moderately acetylated
RHOU	2.770871285	2.77360747	5.656234855	0.001555493	0.003527763	moderately acetylated
SYNGR3	4.894544724	4.453301651	9.984222866	0.011917086	0.009089235	moderately acetylated
ZBTB44	2.370448501	1.714981318	4.830773878	0.00034948	0.000428648	moderately acetylated
CBX7	1.429570272	1	2.906468261	0.008021256	0.00299834	moderately acetylated
PIGQ	4.560206251	2.496153239	9.27081253	0.00116097	0.000114432	moderately acetylated
HOXA10	18.77554686	15.27649317	37.9924159	0.000283619	0.000434078	moderately acetylated
MAF	1.346259775	1.35967794	2.723751479	0.000308487	0.00065505	moderately acetylated
FLJ4028	1	1	2.022361258	0.000816381	0.000816381	moderately acetylated
FUBP3	6.67239059	3.36605974	13.49105044	0.000271482	3.5665E-06	moderately acetylated
PPIG	9.777943644	7.708553087	19.72822484	0.000280787	0.000516547	moderately acetylated
ESRRB	1.145802608	1	2.311345805	0.003813672	0.002405789	moderately acetylated
MAPKA	7.047485778	2.529021099	14.20230569	0.012764815	0.002305436	moderately acetylated
LOC389	1.065310606	1	2.144274921	0.047092592	0.039233782	moderately acetylated
CD82	4.59666343	3.392558771	9.247978277	0.012237857	0.005354103	moderately acetylated
PXDC1	1.522837189	1.380751878	3.059327793	0.026967988	0.021984855	moderately acetylated
SNORA1	12.29816401	1	24.70141917	0.035611035	0.003755117	moderately acetylated
MAPK8I	1.253212372	1	2.515348808	0.000465193	0.000209013	moderately acetylated
SNORA7	10.23843581	1	20.51042795	0.001468878	6.14306E-05	moderately acetylated
ZNF367	1.575207355	1.158553829	3.155082558	0.000926713	0.000526079	moderately acetylated
NFE2L1	3.555533188	3.405801755	7.119146974	8.69594E-05	0.000946595	moderately acetylated
