# Supplementary material for: PCBP1/2 and TDP43 Function as NAT10 Adaptors to Mediate mRNA ac4C Formation in Mammalian Cells
Source: Adv Sci (Weinh). 2024 Nov 18;11(47):2400133. doi: 10.1002/advs.202400133 (PMC11653668; doi:10.1002/advs.202400133)
Supplement: Supplementary file 4 — Supporting Table [file ADVS-11-2400133-s004.pdf]

ADVANCED SCIENCE
Open Access

Supporting Information

for *Adv. Sci.*, DOI 10.1002/advs.202400133

PCBP1/2 and TDP43 Function as NAT10 Adaptors to Mediate mRNA ac^{4}C Formation in Mammalian Cells

Zhi-Yan Jiang, Yu-Ke Wu, Zuo-Qi Deng, Lu Chen, Yi-Min Zhu, Yuan-Song Yu, Hong-Bo Wu and Heng-Yu Fan**

Table S3. ac4C(+) mRNAs in *mouse testes*

Gene Syı	FPKM+1_inpı	FPKM+1_IgG	FPKM+1_ac4(	p-value_ac4C ı	p-value_ac4C ı	Cluster
5330439N	1	1	5.686202537	0.03992386	0.008317447	highly acetylated
Enho	1.142066276	2.177297766	9.833890032	0.000627827	9.96671E-06	highly acetylated
Gm11631	1.577827258	1.291831198	6.181348576	0.017389061	0.001834145	highly acetylated
Gm1189:	1.642553326	1.817076389	6.758182425	0.006668318	0.001141499	highly acetylated
Gm1222	1	1	5.405465989	0.037571071	0.007424214	highly acetylated
Gm1239(	1.113527774	1	5.568514152	0.017285584	0.00171283	highly acetylated
Gm1400(	1	1.097694564	7.151051166	0.037950582	0.008093596	highly acetylated
Gm1578(	1	1.189812885	7.074979418	0.014624756	0.001548889	highly acetylated
Gm1603(	1	1	6.001727109	0.002356877	3.30433E-05	highly acetylated
Gm1715(	1	1.187284787	7.761670672	0.023977943	0.003563093	highly acetylated
Gm2176	1	1.479662292	7.707256122	0.004990746	0.001317254	highly acetylated
Gm2203	1	1	8.264578676	0.007402703	0.000320074	highly acetylated
Gm2218(	1	1.872828105	10.55245731	0.030138003	0.010429681	highly acetylated
Gm2230	1	1	9.126729301	0.002402405	3.43265E-05	highly acetylated
Gm2346	1	1	8.858229176	0.014653175	0.001222063	highly acetylated
Gm2360	1	1	6.664302747	0.049670062	0.012466534	highly acetylated
Gm2362	1	1	6.067975704	0.031450521	0.005311019	highly acetylated
Gm2443(	1	1	9.8508994	0.032145528	0.005535252	highly acetylated
Gm2500	1	1	9.36186825	0.02535281	0.002246507	highly acetylated
Gm2525(	1.842439741	1.998602928	8.028513568	0.020131007	0.004541185	highly acetylated
Gm2526	1	1	6.085044583	0.003510452	7.29981E-05	highly acetylated
Gm2569	1	1	10.2412373	0.010554738	0.00064336	highly acetylated
Gm2586	1	1	7.221210078	0.040442652	0.008520262	highly acetylated
Gm2601	1	1	14.41356159	0.012780753	0.000935894	highly acetylated
Gm2655(	1	1.22184381	9.525238556	0.002760365	7.74252E-05	highly acetylated
Gm2697	1.207698277	1.165255733	7.957949156	0.012400381	1.85856E-06	highly acetylated
Gm3785(	1	1.189706729	7.227964689	0.006938067	0.000882703	highly acetylated
Gm4534	1	1.25897584	7.588739687	0.002251587	7.23019E-05	highly acetylated
Gm4534	1	1	6.570600844	0.039455986	0.008136341	highly acetylated
Gm4811	1	1	6.080639025	0.011825753	0.000803984	highly acetylated
Gm4947	1	1.195745055	6.000221583	0.007496847	0.000548195	highly acetylated
Gm4958(	1.104329922	1.332041612	6.45496894	0.023010283	0.004177757	highly acetylated
Gm8152	1	1.285875459	7.103859392	0.000538842	5.32157E-05	highly acetylated
Gm9378	1.382764553	1	5.970054967	0.00040651	2.50826E-05	highly acetylated
Igkv13-6	1	1	7.41616128	0.022144765	0.002718701	highly acetylated
Mir509	1	1	12.03996382	0.033974279	0.006144762	highly acetylated
mt-Tf	1	1	8.389950184	0.016953404	0.001622627	highly acetylated
Rnu12	1	3.838539135	19.94714614	0.049491719	0.025993597	highly acetylated
Rnu2-10	1	1.561340302	11.62041555	0.001802936	0.00030516	highly acetylated
Snora31	1	1.821927025	10.8055727	0.006122372	0.001209241	highly acetylated
Olfr647-ı	1	16.08592305	72.2767358	0.035005405	0.01502506	moderately acetylated
Gm1556	15.49283666	168.347905	557.3046986	0.001485059	6.71335E-05	moderately acetylated
Lars2	12.87628076	139.0689277	391.2700499	0.000592034	1.2935E-05	moderately acetylated
Gm2393	72.95469962	525.4874473	1926.899728	0.00254228	0.000338203	moderately acetylated
Gm2271(	1	4.168504799	16.69062546	0.015288718	0.007734681	moderately acetylated
Mir466	1	5.037283602	15.67532918	0.00095453	0.003461514	moderately acetylated
Gm2762	1	6.046510648	15.02864708	0.001655132	0.008130728	moderately acetylated
Gm25431	1	5.723528649	13.82473658	0.004646846	0.041862156	moderately acetylated
Gm2412(	1.349383924	3.712500331	16.01682545	0.034716621	0.015185919	moderately acetylated
Mir467d	1	2.792660943	11.82138027	0.048473746	0.022551407	moderately acetylated
Fth-ps2	1	2.293716273	8.666182515	0.004289619	0.000612176	moderately acetylated
Mir466p	2.012820812	5.902404857	17.09169176	0.030079947	0.024825054	moderately acetylated
Snord49b	1	2.733066115	8.461587144	0.048849021	0.016261746	moderately acetylated

Orai1	2.139037262	6.185421203	15.66997013	0.00389249	0.000510649	moderately acetylated
Gm24013	1	1.822082434	6.754653674	0.023895106	0.007812828	moderately acetylated
Gm4908(	1.099274287	2.157306257	7.364779819	0.014704663	0.000283407	moderately acetylated
Ssxa1	1.823205957	3.488040135	12.00502782	0.011215908	0.002389266	moderately acetylated
Gm4411	1	3.056743069	6.446111689	0.035420434	0.037289717	moderately acetylated
Gm2819(	1	1.44368762	6.366047584	0.001298511	0.000526731	moderately acetylated
Hbb-bs	159.2750117	208.5642876	991.593056	0.008623178	0.000559093	moderately acetylated
Gm2805	1	2.025455147	6.030348296	0.00332899	0.002588318	moderately acetylated
Ctdsp2-ps	2.055906284	4.837980237	12.21922889	0.009242803	0.002710382	moderately acetylated
Fbll1	1.35984452	2.888573952	7.725078593	0.004716736	0.002984392	moderately acetylated
Gm4526	1.319649122	2.782461728	7.488352592	0.000198823	0.000281115	moderately acetylated
Zar1	1.078044201	1.473947645	5.87990629	0.006352221	0.000522432	moderately acetylated
Gm1130	1.291090589	3.076414276	6.976165715	0.01219416	0.00532637	moderately acetylated
Gm4356	1	1.674623331	5.385092671	0.033094929	0.016341285	moderately acetylated
Gm4538(	1	1.847439812	5.085012158	0.003364772	0.005460617	moderately acetylated
Gm1219	1	1.093654379	5.004681036	0.021437808	0.00291852	moderately acetylated
Gm2419	1	1	4.958200003	0.031450521	0.005311019	moderately acetylated
Gm6420	1.374029263	2.941958485	6.634047068	0.008223747	0.00551711	moderately acetylated
Gm3406(	1.437047526	2.320306627	6.914810054	0.017585035	0.00585305	moderately acetylated
Gm6025	6.381555359	7.675301431	30.64597681	0.014774676	0.005473351	moderately acetylated
Gm1269	1	1	4.74158258	0.003165359	0.000284813	moderately acetylated
Gm4121	1	1	4.700076308	0.026055524	0.004241791	moderately acetylated
Gm3832	1.422206334	1.380343882	6.658085414	0.010579667	0.0018159	moderately acetylated
Gm4408	1	1	4.669762078	0.008957758	0.000466059	moderately acetylated
4930554(	2.051207607	4.111140047	9.570584269	0.026766997	0.0001822	moderately acetylated
Iglv1	1	1	4.655047559	0.049050721	0.012182201	moderately acetylated
Gm1240	1.469974204	3.215408655	6.842315834	0.042246074	0.040309077	moderately acetylated
Gm43821	1	1	4.651336088	0.037485519	0.007392563	moderately acetylated
Gm2347	1	1.983618794	4.61896492	0.015227421	0.015242601	moderately acetylated
Gm1415	1	1	4.616843875	0.001876727	2.0988E-05	moderately acetylated
Gm4281	1	1.058476128	4.594876032	0.002134332	3.64747E-05	moderately acetylated
Gm2963	1.242967796	1.206343867	5.680386608	0.000535827	3.01059E-05	moderately acetylated
Fbxo48	1.040116178	1	4.737131545	0.000779881	5.32691E-05	moderately acetylated
Gsc	1.35544579	3.045731915	6.146379012	0.010490058	0.005966194	moderately acetylated
Gm3434	1	1	4.461387813	0.017312729	0.001690001	moderately acetylated
Gm47791	1	1	4.460708211	0.019833854	0.002198527	moderately acetylated
Pafah1b1	2.206476799	4.027049747	9.544188526	0.000623697	0.000203858	moderately acetylated
Hnrnpl	2.991189043	6.269079073	12.83581881	0.001017011	8.88296E-05	moderately acetylated
Gm2570	2.193921222	1.705198866	9.409389889	0.001031986	0.000847392	moderately acetylated
Wdr46-ps	1	1	4.267353935	0.000167078	7.34772E-06	moderately acetylated
Gm2188	1	1	4.266030456	0.033007036	0.005818887	moderately acetylated
Gm1832	1	1.085017987	4.242381701	0.027651939	0.004782529	moderately acetylated
Gm13211	1	1	4.196841595	0.00269164	4.3044E-05	moderately acetylated
Gm1386	1.570512989	2.700153255	6.530320345	0.00707329	0.004225487	moderately acetylated
H4c14	1	1.066000909	4.144657332	0.005006397	0.000189202	moderately acetylated
2310026I	1.225352631	1.381854162	5.071659449	0.023937035	0.005466442	moderately acetylated
Gm2818	1	1	4.138388542	0.000944872	5.33819E-06	moderately acetylated
Gm42721	1.088779763	1.858585727	4.448333836	0.020320436	0.0071537	moderately acetylated
Gm2537(	1.677752273	1	6.800989654	0.003372205	0.000404953	moderately acetylated
Nxph3	1.128834281	2.197055406	4.565350973	0.025536897	0.01981709	moderately acetylated
4930573I	2.613503322	3.647634202	10.5047059	0.041821056	0.018725365	moderately acetylated
Krt23	1.748775514	2.165627845	7.007691783	0.016477603	0.003342114	moderately acetylated
Gm1709	1	1	3.977082777	0.001090365	7.10495E-06	moderately acetylated
Gm1294	1.214621553	1.2774183	4.801455889	0.001352763	0.000207521	moderately acetylated
Gm6044	1.180643391	1.091233801	4.662007336	0.016052205	0.002050605	moderately acetylated

Egln1	1.376214742	1.628913924	5.410102218	0.006798327	0.00045564	moderately acetylated
Echdc2	2.143021651	2.029586772	8.357941253	0.012994818	0.001388794	moderately acetylated
Gm1372(	1	1.022352021	3.898739525	0.028085428	0.005864074	moderately acetylated
Pla2g4d	1.904343529	1.688852049	7.393435173	0.003563068	0.000331659	moderately acetylated
Gm4967{	1	1	3.867823174	0.045451744	0.010285929	moderately acetylated
Gm1501{	1.72694397	1.448928211	6.654367998	0.02750845	0.005716694	moderately acetylated
Gm4769{	1.487248931	2.553023924	5.729438981	0.002242549	0.000685618	moderately acetylated
Gm9402	1.2323223	1.183905937	4.746093903	0.002186964	0.000243472	moderately acetylated
Gm1910(	1	1.078682263	3.847174824	0.041550669	0.009977043	moderately acetylated
Gm2944(	1.168173605	1.975215526	4.479681544	0.012805025	0.007992175	moderately acetylated
Gm47961	1.356288744	1	5.195123874	0.034444264	0.004058916	moderately acetylated
Gm4296(	1.185170609	2.230740396	4.53352508	0.044065214	0.034669865	moderately acetylated
Gm2636(	2.629055161	3.382157889	10.03529139	0.036969148	0.034006662	moderately acetylated
Gm1201(	1.089871438	1.530059547	4.150115782	0.011321733	0.003724163	moderately acetylated
1700048(	9.177744948	10.91777336	34.74928876	0.006721757	9.51378E-05	moderately acetylated
Scgb3a1	1	1.359804288	3.763855437	0.037241313	0.021517112	moderately acetylated
Gm1644(	1.165548305	1	4.381035925	0.002920087	7.34053E-05	moderately acetylated
Gm4265(	1.205566824	1.99182197	4.527138341	0.016423195	0.00564684	moderately acetylated
Gm1229(	1	1	3.754847175	0.047341523	0.011411704	moderately acetylated
Rtl10	2.302616362	2.432451741	8.597453176	0.0135895	0.001520582	moderately acetylated
Gm1614(	1	1	3.727287957	0.00492666	0.000266318	moderately acetylated
Gm1907{	1	1.12524255	3.721866407	0.00084742	6.23551E-05	moderately acetylated
Gm4350(	1	1	3.679722384	0.0053182	0.000166444	moderately acetylated
Gm4745(	1	1.675902205	3.670459891	0.015018888	0.015355891	moderately acetylated
Platr22	1.222983432	1.461850767	4.465530093	0.001327609	3.23825E-05	moderately acetylated
9930024(	1.47093816	1.611315465	5.370532128	0.007796717	0.002041436	moderately acetylated
Rhox10	1.118140304	1.046520327	4.074214124	0.045003273	0.009771901	moderately acetylated
Gm4851(	2.269592285	1.536329642	8.269651834	0.013904611	0.003332996	moderately acetylated
9530078(	1.389658727	2.327791846	5.047429848	0.026329958	0.027772366	moderately acetylated
Gm1712(	1.242640787	1.288357003	4.512767634	0.013998516	0.00318896	moderately acetylated
Gm4381(	1.388539018	1	5.035608198	0.002789838	3.0918E-05	moderately acetylated
Gm1558(	1.237525829	1.427467972	4.47975809	0.001354245	9.42203E-05	moderately acetylated
Rpsa-ps5	2.476887297	4.41860962	8.947318015	0.008501266	0.002728813	moderately acetylated
Arpin	1.237525829	1.16549795	4.466240794	0.007719563	0.000546922	moderately acetylated
5330431(	1.275520282	1.949944035	4.593599415	0.006461412	0.001085605	moderately acetylated
Stbd1	1.405857957	2.469051338	5.034805495	0.011998156	0.00689162	moderately acetylated
Gm1864(	1.391067472	1.07778849	4.97240876	0.049963404	0.011131989	moderately acetylated
S100a9	1.172684008	1.495902699	4.180482611	0.003941158	0.001933091	moderately acetylated
4930524(	2.285568698	3.20473031	8.112784945	0.001627617	0.000559265	moderately acetylated
Tcf24	1.021497985	1.705779706	3.615241487	0.000166115	8.42546E-05	moderately acetylated
Gm1416(	1.164116618	1.38562838	4.116427849	0.026634752	0.005707457	moderately acetylated
AL77240	1.272603989	1.428593445	4.497913192	0.038840381	0.012617119	moderately acetylated
Gm3823(	1	1.17138293	3.528204343	0.016442297	0.0022608	moderately acetylated
Gm3102(	1	1.488647146	3.526406921	0.044528197	0.033372033	moderately acetylated
Gm4727(	1	1	3.505882719	0.002181431	2.8325E-05	moderately acetylated
Gm4712(	1	1.285540319	3.500400228	0.008820948	0.000737451	moderately acetylated
Gm4856(	1	1	3.489768676	0.039978381	0.008317447	moderately acetylated
1700074(	4.256540906	3.269869084	14.82724162	0.028977981	0.003910458	moderately acetylated
Gm4313(	3.124536585	3.091922427	10.85698141	0.002445287	0.001133919	moderately acetylated
2900045(	1.279074465	1.727244992	4.430995022	0.016801142	0.00582549	moderately acetylated
Gm1355(	1	1.151346336	3.455221616	0.026418082	0.005214061	moderately acetylated
Gm4806(	1.103136215	1.322982482	3.808984192	0.035321732	0.009147976	moderately acetylated
Gm4868(	1.530241484	1.875246293	5.281935897	0.025073477	0.011567012	moderately acetylated
Fv1	1.555216626	1.98116203	5.351893754	0.00985149	0.002163342	moderately acetylated
Rnf183	1.059147672	1.139983951	3.638439086	0.039969921	0.009850837	moderately acetylated

Serpinb6c	3.36531158	4.132422318	11.55739057	0.00292406	0.000184468	moderately acetylated
Gm14037	1	1	3.426444873	0.001352889	1.09276E-05	moderately acetylated
4930500/	5.347111711	7.485337707	18.22931169	0.009320491	0.003437282	moderately acetylated
Gm31812	1.154087269	1.069560821	3.93297317	0.04972464	0.012069954	moderately acetylated
AC10961	1.851279612	1.973066649	6.27760286	0.005257173	0.000976607	moderately acetylated
Gm7497	1.544658926	2.416079609	5.201957064	0.005943858	0.007299051	moderately acetylated
Gm43175	1.070977207	1.009412203	3.595133544	0.020838775	0.002495955	moderately acetylated
Gm36262	1	1.071132284	3.355110088	0.019163324	0.002511136	moderately acetylated
Gm10923	1	1.298518565	3.345757952	0.041690795	0.017542375	moderately acetylated
4930401(	1.573151199	1.895269491	5.261799532	0.031695741	0.008819498	moderately acetylated
Gm12034	6.083142045	9.08631169	20.34510489	0.005750276	0.0046381	moderately acetylated
Gm44886	1.069126574	1.716128841	3.568303348	0.001670544	0.000353693	moderately acetylated
Slc25a13	2.068021946	1.579393838	6.900992807	0.001032359	6.85416E-05	moderately acetylated
Dntt	1.157314227	1	3.859274241	0.01182735	0.00072443	moderately acetylated
Krt39	1.110399329	1	3.695035128	0.00585328	0.000302166	moderately acetylated
Gm4752(	1.183961331	1	3.939799509	0.002821625	3.65698E-05	moderately acetylated
Metrnl	2.648228572	2.276587526	8.808395501	0.014187783	0.001554144	moderately acetylated
Gm8557	1.189770636	1.236661197	3.956725647	0.03844387	0.009851983	moderately acetylated
Gm6658	1.095640374	1.22023026	3.625967589	0.01560365	0.002220114	moderately acetylated
Dnm1	1.171729953	1.915575909	3.864309608	0.000620315	5.24667E-05	moderately acetylated
Gm12247	1.538155536	1.59420523	5.070350787	0.016262342	0.005036101	moderately acetylated
Gm4846	1.206745533	1	3.964474598	0.003669222	0.000221374	moderately acetylated
Gm43415	1	1.441750888	3.280221995	0.020228807	0.007216266	moderately acetylated
Rnf130	1.968964305	3.187285086	6.444291868	0.000325458	0.000666615	moderately acetylated
D730050	1	1	3.266698188	0.034857968	0.006761788	moderately acetylated
Gm47367	1.273361796	2.069303864	4.149027125	0.013333284	0.015228474	moderately acetylated
Gm50425	1	1	3.241486642	0.036517351	0.007038478	moderately acetylated
Gm20511	1	1	3.234817422	0.035915962	0.00682235	moderately acetylated
Gm47152	1.412073382	1.398091088	4.565178024	0.03529636	0.008422838	moderately acetylated
Gm50295	1	1	3.232038213	0.045283358	0.010511944	moderately acetylated
Gm15551	1.168487948	1.436255807	3.77229156	0.000453202	0.000197064	moderately acetylated
Gm26877	2.566432188	2.092032174	8.27754423	0.017331521	7.26285E-06	moderately acetylated
Gm9172	1.755899809	1.996585173	5.648826763	0.049914748	0.038080457	moderately acetylated
Gm1325(	1.718255397	1.276624155	5.525086084	0.011832812	0.000829588	moderately acetylated
Gm45081	3.521428035	3.698352503	11.3043952	0.0122042	0.000426879	moderately acetylated
Nr6a1os	1	1.167619464	3.200732117	0.02307032	0.003796835	moderately acetylated
Gm30978	1	1	3.197911347	0.001316512	2.23516E-05	moderately acetylated
Gm32618	1.067572003	1.479778123	3.40187212	0.01175295	0.010281276	moderately acetylated
1700011I	2.138966364	2.689134176	6.808846504	0.04412281	0.023992894	moderately acetylated
C030015.	1.141342301	1.474783101	3.623527374	0.019403368	0.003798661	moderately acetylated
Gm31393	6.171064276	7.378115843	19.54329004	0.003137606	0.000564053	moderately acetylated
Gm13823	1	1.286284583	3.16029327	0.04680685	0.021885181	moderately acetylated
Gm42743	1.149141384	1.737452016	3.624542243	0.015504186	0.007624904	moderately acetylated
Hoxd9	1.168803469	1.701354158	3.683426175	0.00481905	0.001628694	moderately acetylated
Tcf15	1	1.529034068	3.148165335	0.033782249	0.021498718	moderately acetylated
Gm13594	1	1.170211505	3.145096398	0.038196228	0.010952653	moderately acetylated
Gm11476	1	1.209556943	3.143584365	0.014823015	0.002623454	moderately acetylated
Gm14235	1.123143514	1	3.526205882	0.014851139	0.001537596	moderately acetylated
Gm8601	1	1	3.138701008	0.000538842	1.73867E-06	moderately acetylated
Gm807	1.289068046	1.018971144	4.037141924	0.031095043	0.004761689	moderately acetylated
Olfr896-p	1	1.094821704	3.131093796	0.027452423	0.004926983	moderately acetylated
Olfr29-ps	1.561626494	1.410631578	4.871487527	0.024842301	0.005175031	moderately acetylated
Rasl11a	1.375274989	1.642419712	4.278509	0.035553182	0.010758539	moderately acetylated
Gm27008	1.258877232	1.312181393	3.911892223	0.00569776	0.001098807	moderately acetylated
4933402(	2.26284566	1.384499504	7.018251445	0.025219019	0.002405268	moderately acetylated

Sez6l	1.564309718	1.961798167	4.851359751	0.011707379	0.001785853	moderately acetylated
Igdcc3	1.025011391	1	3.173317693	2.04634E-06	1.67629E-08	moderately acetylated
Gm1348(	1.180282105	1.467890167	3.636919042	0.030725938	0.008320558	moderately acetylated
Gm1926:	1.554559046	1.393989209	4.783244094	0.0299046	0.00281561	moderately acetylated
Eogt	1.340328463	1.327158925	4.121281568	0.006416504	0.000993627	moderately acetylated
Frs3	2.873135677	3.877002899	8.802511865	0.008906113	0.001414026	moderately acetylated
Gm36241	2.31680156	3.12630569	7.095990999	0.012411933	0.004747589	moderately acetylated
Gm4257(	1.348401818	1.805199136	4.125405873	0.003274725	0.002506892	moderately acetylated
1700106(	3.874657703	2.772313359	11.81901467	0.001455727	0.000103437	moderately acetylated
Gm2909:	1.771378198	1.751219835	5.382878996	0.021312578	0.005539503	moderately acetylated
Gm2196(	1	1	3.038808336	0.01474997	0.002209319	moderately acetylated
Gm4531(	2.112852497	2.552529913	6.412827172	0.047918012	0.038882734	moderately acetylated
Nme4	2.310520987	2.786646011	7.006128133	0.010321649	0.004687312	moderately acetylated
Gm19951	1.712711633	1.383398869	5.193167816	0.007609289	0.000683802	moderately acetylated
Gm7706	1	1.176529171	3.021930696	0.023309637	0.005361696	moderately acetylated
Gm4571(	2.048151771	1.931338983	6.178125768	0.01981445	0.018431567	moderately acetylated
Gm4549(	1	1.061355346	3.015653629	0.017789401	0.00378176	moderately acetylated
Alx3	1	1	3.013250238	0.035391737	0.006636358	moderately acetylated
Gm2986:	1.443171348	1.545407439	4.342856774	0.01830919	6.60109E-05	moderately acetylated
Gm2008:	1	1	3.00391919	0.034277848	0.006248647	moderately acetylated
Ppic	1.642487899	2.208547403	4.932917032	0.028796153	0.014609011	moderately acetylated
Gm1413:	1.125544641	1	3.377350196	0.002783737	0.000251965	moderately acetylated
Gm38341	1	1	2.997844467	0.030034908	0.004867092	moderately acetylated
Gm1247:	1.766071266	1.836976408	5.283452612	0.007000826	0.000840065	moderately acetylated
A730043	1	1.14579854	2.989155884	0.034077022	0.010726659	moderately acetylated
Gm6218	1.287073415	1.14130179	3.822399735	0.008798577	0.001257955	moderately acetylated
Gm2703:	1.525015089	1.457649229	4.500993307	0.01158944	3.69681E-05	moderately acetylated
Zfp286os	2.896375574	3.309839091	8.548288705	0.017503797	0.001387611	moderately acetylated
Olfr1003-	1	1	2.939192696	0.020400862	0.002321424	moderately acetylated
Frmpd1os	2.194640452	1	6.449039146	0.020556094	0.001221044	moderately acetylated
Gm5676	1.133939156	1.097507297	3.330882337	0.019935966	0.002362586	moderately acetylated
Olfr682-p	1	1	2.932885534	0.013753551	0.002048143	moderately acetylated
5330406N	1.387425726	1.296211245	4.062094629	0.024079252	0.003993789	moderately acetylated
Gm29791	1.199427107	1	3.503269643	0.046753532	0.011183253	moderately acetylated
Gm3915:	1.263262419	1.208184599	3.683857282	0.042537206	3.0777E-05	moderately acetylated
Asgr1	1.566568525	1.680089571	4.544993006	0.00824257	0.001210276	moderately acetylated
A330043	1.446685095	1	4.191394504	0.040092574	0.004789657	moderately acetylated
Gm1400:	1	1	2.896702977	0.01042749	0.000628227	moderately acetylated
Gm5021:	1.030157595	1.059865168	2.975723725	0.033770907	0.00649038	moderately acetylated
Pnlip	1.266932501	1.236197443	3.658747533	0.011516473	6.838E-06	moderately acetylated
Gm2044:	1.549640563	1.762961169	4.455247408	0.02580612	0.014179252	moderately acetylated
Gm4807:	1	1	2.874825983	0.043250009	0.009653746	moderately acetylated
Gm4514:	1.527140657	1.699626873	4.389494497	0.047017102	0.022250479	moderately acetylated
9330162(	1.02349871	1.397988967	2.938383416	0.004553884	0.000422288	moderately acetylated
Pianp	1.158086728	1.235552626	3.322638931	0.01179716	0.002454936	moderately acetylated
4930570I	2.107383934	1.565540866	6.026591576	0.01408318	0.001203657	moderately acetylated
Snord104	23.53526307	23.62779636	67.12786681	0.012351537	0.00128318	moderately acetylated
Gm45574	1	1	2.84806261	0.038517901	0.007778408	moderately acetylated
4930456(	3.2365825	3.248741636	9.200951703	9.97296E-05	0.000102516	moderately acetylated
Rps2-ps9	1	1.374209303	2.840926728	0.016900833	0.00586911	moderately acetylated
Gm2894(	1.107183177	1.063743162	3.138303032	0.025035762	0.004191532	moderately acetylated
D730003	1	1	2.830504655	0.014209429	0.001150974	moderately acetylated
Crebzf	1.647875316	2.25338589	4.652559779	0.005618264	0.001223375	moderately acetylated
D630033	1	1.357588382	2.822391759	0.013379655	0.007031607	moderately acetylated
Gm1120:	1	1.082355011	2.811018635	0.033491829	0.007811347	moderately acetylated

Rpl27-ps1	1.773268831	2.27594229	4.978561369	0.014835829	0.00142962	moderately acetylated
Ube2ql1	1	1	2.804443805	0.000418817	1.05083E-06	moderately acetylated
Gm7676	7.499198126	8.458277705	21.03089323	0.032813609	0.010465905	moderately acetylated
Gm34933	1	1	2.795184616	0.043397527	0.009714966	moderately acetylated
Snord71	10.54434671	5.504573832	29.44056407	0.022693195	0.000173598	moderately acetylated
Ccl5	2.455402406	2.921874855	6.843094772	0.014819653	0.005534871	moderately acetylated
Gm50107	2.118771925	2.105043289	5.876343066	0.008293179	0.001311603	moderately acetylated
Gm16725	1	1	2.772046749	0.007165223	0.000300124	moderately acetylated
4930422(	1.076326039	1.1246216	2.976927889	0.014515991	0.001814355	moderately acetylated
Gm10855	1	1.070132238	2.754537507	0.017041489	0.002004253	moderately acetylated
Efna3	1.574028887	1.603507168	4.317637611	0.002080914	0.000205981	moderately acetylated
Gm42872	1	1	2.73969368	0.035822644	0.006789077	moderately acetylated
Nat2	2.184507914	1.722022431	5.96960676	0.00069219	6.59841E-05	moderately acetylated
Gm18609	2.276643617	2.279851744	6.218345529	0.008441528	0.000582331	moderately acetylated
Gm1402(	2.089317852	2.444731268	5.68465842	0.008681461	0.002249096	moderately acetylated
Gm1687(	1.059303324	1	2.881804508	0.033999841	0.006066617	moderately acetylated
1700085I	1.566924857	1.143162475	4.259689837	0.007715053	0.001323546	moderately acetylated
Gm18783	1.548207963	1.543885877	4.207564988	0.001646472	0.0005562	moderately acetylated
Gm49802	1.235048376	1.138833154	3.353562634	0.028171148	0.004965218	moderately acetylated
1700012(	13.24585035	13.53065412	35.96258484	0.006047312	0.000266187	moderately acetylated
Gm10373	1	1.281791428	2.711874636	0.022085629	0.005445165	moderately acetylated
Gm3803(	1.853967864	1.758544599	5.00549865	0.04701743	0.011902891	moderately acetylated
2010001/	6.049918895	6.191730189	16.31729224	0.033842216	0.000414646	moderately acetylated
9530056I	1.027398496	1.010899846	2.762801249	0.02284781	0.002936198	moderately acetylated
Aqp5	1.104048925	1.062628827	2.968185877	0.006435764	0.00024274	moderately acetylated
Gm15832	1.123009078	1.04970051	3.017600285	0.013938419	0.001370887	moderately acetylated
Gm48447	3.47810453	3.210350249	9.345204447	0.018568282	0.002111944	moderately acetylated
Smc2os	3.879905823	4.498664347	10.41568782	0.022781055	0.005494337	moderately acetylated
Gm15867	1	1.046211977	2.683819858	0.021193297	0.004848866	moderately acetylated
Hs3st1	1.161687986	1.116572943	3.110824562	0.014702731	0.002410836	moderately acetylated
Gm1211(	1.130167585	1.397872219	2.998765897	0.003012601	0.000742648	moderately acetylated
4930518I	4.341370092	5.363098132	11.49881404	0.003386483	0.001093022	moderately acetylated
Gm12973	2.039517901	1.08801525	5.397861615	0.039071995	5.663E-05	moderately acetylated
Gm19531	1.916466762	2.278233103	5.071806927	0.045804041	0.027598304	moderately acetylated
9430031I	1.389799145	1.369415468	3.657154523	0.004501715	0.001590556	moderately acetylated
Gm20531	1.131305247	1.084862117	2.975934813	0.007733525	0.000541843	moderately acetylated
4930504(	1	1	2.625706428	0.045972438	0.010809737	moderately acetylated
Trav7-6	1	1	2.621197235	0.009417775	0.000514307	moderately acetylated
Btn1a1	1.065652624	1	2.784516358	0.001725557	6.51744E-05	moderately acetylated
Gm18958	1	1.129856855	2.609206498	0.001222127	2.15986E-05	moderately acetylated
A930024	3.852564945	4.899082279	10.03174825	0.00205004	0.00090976	moderately acetylated
Bicdl1	1.307448773	1.348997799	3.401353254	0.027800847	0.004966718	moderately acetylated
Cfhr1	1.630193789	1.194962894	4.235883236	0.001565057	0.000123593	moderately acetylated
Uhrf2	1.734560379	2.062360083	4.506053203	0.001543199	0.000164125	moderately acetylated
Gm7680	1.133740434	1.184863528	2.934430357	0.012906568	0.001817147	moderately acetylated
Iffo2	1.087008738	1.206953643	2.802459734	0.000398201	2.0811E-05	moderately acetylated
Spred3	1.891552704	2.324458072	4.87371039	0.001458725	0.000104074	moderately acetylated
Ucn	1.536553883	1.090328704	3.950702909	0.002894387	0.000206084	moderately acetylated
Trac	1.530241484	1.249102106	3.919175152	0.047622705	0.011050909	moderately acetylated
Rab26os	1.906851632	2.033029846	4.871124227	0.038661862	0.003921697	moderately acetylated
Gm8885	1.753687728	1.630165272	4.46456573	0.044354766	0.010145886	moderately acetylated
Gm2632(	1.589157204	1.175189539	4.044508561	0.039098044	0.012656304	moderately acetylated
Gm41253	3.70658512	3.553729711	9.432856887	0.011803811	0.001325188	moderately acetylated
Gm4313(	1	1	2.526904578	0.003507132	7.28609E-05	moderately acetylated
Aldh4a1	1.854199904	2.011732285	4.676009384	0.00012594	0.000179104	moderately acetylated

Gm40332	2.921984057	2.612203605	7.367455057	0.016944723	0.007578339	moderately acetylated
Rab25	1.053879888	1	2.652035173	0.001327286	4.1674E-05	moderately acetylated
Tcl1	1.488569389	1.019238488	3.738603237	0.006250606	0.000770128	moderately acetylated
Gm47572	1.053639424	1.226682803	2.628106457	0.00329774	0.000162472	moderately acetylated
C920006(	5.650629082	5.347027839	14.08624932	0.011541895	0.001068418	moderately acetylated
Nek3	2.00482362	1.825631889	4.992075739	0.023318216	0.003238071	moderately acetylated
Thap3	1.675720032	1.228724022	4.167189465	0.00283865	0.000259902	moderately acetylated
Inf2	1.018505656	1.166867092	2.531728075	0.000336917	9.16219E-05	moderately acetylated
Gm37968	1	1.212816529	2.485679608	0.007852603	0.00071328	moderately acetylated
Sox10	1.177268539	1.270414018	2.921696526	0.00961519	0.001717748	moderately acetylated
Rnase10	7.442513646	6.455785936	18.46974497	0.027748155	1.14349E-06	moderately acetylated
Gm43429	1.040044892	1.167571887	2.558224817	0.032943128	0.008560061	moderately acetylated
Gm14366	1	1	2.45774141	0.02113957	0.002486171	moderately acetylated
Zfp583	2.346671792	1.702751903	5.762653709	0.006040406	0.000314866	moderately acetylated
Fam180a	1	1	2.453189962	0.030835808	0.005370612	moderately acetylated
Abca9	2.500857481	2.386968781	6.127745367	0.000704333	4.11487E-05	moderately acetylated
Il1r2	1.141731214	1	2.795685465	0.016552953	0.001314107	moderately acetylated
Gm45505	1.427884742	1.510606361	3.483800891	0.005085387	0.00243586	moderately acetylated
Gm43091	2.385146193	1.169553163	5.801463744	0.030531908	0.000493774	moderately acetylated
Gm36903	1	1	2.429509097	0.014697165	0.001629407	moderately acetylated
Cd200r2	1.412073382	1.568730147	3.430436198	0.000392137	0.000285232	moderately acetylated
Gm16577	8.27252651	5.797259491	20.08690809	0.015363723	0.002665393	moderately acetylated
Gm46350	1.217207355	1.172821658	2.953985017	0.029419194	0.01168191	moderately acetylated
Jph2	1.233032098	1.31480226	2.987016699	0.013831683	0.003228826	moderately acetylated
Chrna4	1.035898468	1.176907058	2.508920355	0.000614455	1.38104E-05	moderately acetylated
Gm47376	5.964289835	6.82661909	14.43779336	0.005440718	0.005154614	moderately acetylated
Gm20125	1.438998631	1.171255217	3.48318399	0.007414358	0.00071598	moderately acetylated
Gm43197	1	1	2.418230588	0.006753942	0.000267054	moderately acetylated
Olfr1296-	1	1	2.413699589	0.004558089	0.000122602	moderately acetylated
G0s2	3.380303332	3.271354008	8.145030297	0.005883719	0.001089312	moderately acetylated
Grhpr	6.510806689	6.008063075	15.67430375	0.006493067	0.000884044	moderately acetylated
Gm5896	1.088028371	1	2.616638567	0.015166672	0.001054655	moderately acetylated
Tbr1	1.032862214	1	2.481884703	0.048969283	2.08496E-07	moderately acetylated
Gm16063	1.291461368	1.125632585	3.102732102	0.034688917	0.00450924	moderately acetylated
Gm26802	1.252260407	1.037959963	3.006046494	0.009127849	1.86374E-07	moderately acetylated
Gm49240	2.670685046	3.198264932	6.409473027	0.001545819	0.003907211	moderately acetylated
Gm43648	1	1	2.395933445	0.034544848	0.006340648	moderately acetylated
Psg19	3.840638465	4.123716735	9.186575219	0.03051111	0.007948633	moderately acetylated
Gm43557	1.133119697	1.075038199	2.707896952	0.021483334	0.003448949	moderately acetylated
Ferd3l	1.050869668	1.131920057	2.509327377	0.005042155	0.000799102	moderately acetylated
2900026A	1.10002706	1.187343224	2.623432872	0.022997821	0.003937437	moderately acetylated
Gm29488	1	1.052835898	2.382253024	0.001770004	5.38335E-06	moderately acetylated
Etv2	3.236323816	3.517960287	7.689661907	0.010959574	0.002244652	moderately acetylated
Gm31497	7.075337379	5.539188198	16.79186707	0.047001328	0.006072747	moderately acetylated
Micall2	1.467671965	1.669157069	3.479821866	0.022036313	0.00442398	moderately acetylated
Gm12280	1.111011148	1.138215919	2.629426435	0.032697998	0.006557764	moderately acetylated
5430403N	1	1.069659433	2.362424841	0.001808631	0.00012381	moderately acetylated
Gm48789	3.096303541	3.433574773	7.311673249	0.003715611	0.041706492	moderately acetylated
Ly6g6d	1	1	2.360938547	0.045513975	0.009818373	moderately acetylated
Bcas3os1	1.024914608	1.174800462	2.409526552	0.002864812	0.000380605	moderately acetylated
Gm2451	1.180462567	1.045571238	2.769077451	0.030285137	0.003843299	moderately acetylated
Adm	1.556769934	1.465520485	3.651715043	0.003890681	0.000851485	moderately acetylated
Apol10b	1.038770345	1.019580932	2.435668159	0.000586919	1.73733E-05	moderately acetylated
Gm6122	2.460311598	1.76065219	5.761364832	0.00201087	9.99178E-05	moderately acetylated
A630014	1.151040637	1.086872216	2.689112237	0.024910329	0.003000201	moderately acetylated

Pygb	2.462050754	2.695423936	5.725175269	0.003957891	0.000746988	moderately acetylated
4930429I	4.701444175	4.964588976	10.93015707	0.00378113	9.10189E-07	moderately acetylated
A130023	1.139450885	1.181654509	2.648756103	0.000512373	4.71411E-05	moderately acetylated
Tagln	1.136027745	1.099915903	2.636240229	0.04540988	0.010877953	moderately acetylated
Gm3399	1	1	2.314762097	0.029212612	0.006677596	moderately acetylated
Mkrn3	1.778603514	1.408495461	4.11629428	0.017668335	0.00353866	moderately acetylated
2610307I	2.532917986	1.427344706	5.856998255	0.007337181	0.000608893	moderately acetylated
Prr5	4.056425872	3.314439441	9.372045035	0.014924994	0.001124471	moderately acetylated
Gm3545	3.995624093	3.717388751	9.223091718	0.012119072	0.012426109	moderately acetylated
4930515I	4.355504484	4.17862736	10.0336895	0.048575127	0.014966018	moderately acetylated
Gm49451	6.158674678	5.419271017	14.16980323	0.015785817	0.002437023	moderately acetylated
Gm4363	1.123227686	1.101224758	2.58231014	0.017730287	0.002973856	moderately acetylated
Gm4411	1	1.034487919	2.298172687	0.004982601	0.000174037	moderately acetylated
Scd3	2.497275361	2.483519419	5.733739702	0.000309681	0.000101985	moderately acetylated
Ifi204	1.303760917	1.197454836	2.991010427	0.000505139	1.17741E-05	moderately acetylated
Gm1545	1.373254875	1.562259131	3.148575044	0.019198421	0.018698071	moderately acetylated
Gm1065	3.83908826	4.37653668	8.801730907	0.033473028	0.002721243	moderately acetylated
4930405	1.170077457	1	2.680658412	0.036799152	0.007987584	moderately acetylated
Smim26	9.325129031	8.939776256	21.34390136	0.027237482	0.011176635	moderately acetylated
Gm4243	1.191789473	1.037955055	2.72586354	0.010698715	0.000911781	moderately acetylated
G730013	1.094191277	1.012490594	2.500598239	0.021206323	0.002214502	moderately acetylated
Hoxb7	1.233526042	1	2.816669412	0.001951153	4.67337E-05	moderately acetylated
Cxcl10	1.120081331	1.047771529	2.553110927	0.0130336	0.002002604	moderately acetylated
Slc35a1	6.071171279	6.272718616	13.81925156	0.028732191	0.0001448	moderately acetylated
Gm3746	1	1	2.273116718	0.049438547	0.013600554	moderately acetylated
Gm4852	1.106633737	1.181528415	2.512190381	0.012913468	0.003049623	moderately acetylated
Rhd	1.508951989	1.417381196	3.417058372	0.00647848	0.001004563	moderately acetylated
Zc3h12d	1.198257446	1.075626599	2.709500881	0.024681951	0.004998642	moderately acetylated
Cdh1	1.118243619	1	2.525399186	0.012829822	0.000700298	moderately acetylated
Gm1258	3.119303737	1.789864268	7.034679765	0.033934995	3.65115E-05	moderately acetylated
Gm3669	1.619719734	1.426540806	3.650912868	0.026354855	0.01159104	moderately acetylated
Gm2192	1.545648016	1.48501695	3.481310509	0.006097586	0.000967983	moderately acetylated
Rnf217	1.098219616	1.208886689	2.46956323	0.005756844	0.00041758	moderately acetylated
Gm32921	3.559560743	3.48530712	7.994172794	0.011511919	0.005969624	moderately acetylated
1700057I	4.09589876	3.651132941	9.192910781	0.001044331	0.000105425	moderately acetylated
Gm9316	1.212096594	1.016875527	2.698439643	0.030078829	0.003420869	moderately acetylated
Mageb3	2.694235925	2.72552247	5.997865093	0.000767776	7.24813E-05	moderately acetylated
4930553I	8.770780368	9.365224799	19.52385631	0.013664594	0.005140439	moderately acetylated
Cd300ld	1.034079793	1	2.297152509	0.001198287	2.80611E-05	moderately acetylated
Gm4316	1	1.020053678	2.218524069	0.022786891	0.003119691	moderately acetylated
Borcs5	6.863125108	5.704148679	15.21846409	0.001501909	7.21214E-05	moderately acetylated
Gm1200	5.519607077	5.546900428	12.23716817	0.002896573	0.000344272	moderately acetylated
Slc2a4rg-	1.090867996	1.205175126	2.411813924	0.004543046	0.000894786	moderately acetylated
A930006	4.485036339	4.769977582	9.911549269	0.023574325	0.004018261	moderately acetylated
Gm3822	1.056267823	1.164474924	2.333644251	0.00501355	0.001471734	moderately acetylated
Fcmr	6.659778051	6.08231066	14.69559806	0.001630965	3.20441E-05	moderately acetylated
Acnat2	1.206745533	1.154390683	2.655145239	0.015946113	0.001827193	moderately acetylated
Gm42671	1.280988318	1.351380031	2.815061265	0.013456981	0.004102867	moderately acetylated
Zfp14	2.141354331	1.492171146	4.703571485	0.001587505	0.000148566	moderately acetylated
Gm15441	3.76984737	3.562154448	8.274108705	0.005216939	0.002716023	moderately acetylated
4921511I	2.411604845	2.161712686	5.287384417	0.007152437	0.00239274	moderately acetylated
Gm4919	1	1	2.191060015	0.007636628	0.000340335	moderately acetylated
Gm4732	1.113527774	1	2.437711733	0.023311684	0.001629585	moderately acetylated
Gm4285	1.126425038	1	2.464418368	0.005636979	0.000291251	moderately acetylated
Gck	2.604885204	2.252544243	5.697183896	0.024925675	0.004337101	moderately acetylated

Tek	2.013895537	1.921290077	4.401613285	0.013002996	0.001979563	moderately acetylated
Sh3bp2	2.154040474	1.812826005	4.68633615	0.024820128	0.002709746	moderately acetylated
Tab3	1.325860108	1.224023801	2.882243079	0.006168362	0.000323211	moderately acetylated
Gm8873	1.187924362	1	2.574681194	0.031784517	7.28338E-05	moderately acetylated
Ripk3	2.61247083	1.653866899	5.655449208	0.002080104	9.17893E-05	moderately acetylated
Gm16617	1	1	2.16462046	0.012116841	0.001912286	moderately acetylated
H3c15	2.811658403	2.581479796	6.081294879	0.031462743	0.015801737	moderately acetylated
Gm4411	1.099165074	1.026166301	2.375698206	0.006480844	0.000450666	moderately acetylated
Gm1853	2.47966271	2.198492469	5.352570521	0.012738693	0.002393281	moderately acetylated
Mmgt1	1.851592369	1.851758196	3.995771415	0.01251992	3.81507E-05	moderately acetylated
Gm4707	8.741120901	7.99604139	18.83513641	0.028412242	0.01364445	moderately acetylated
Gm4758	1.210719343	1.009219483	2.607254019	0.031401069	0.003380258	moderately acetylated
Tram1l1	1.139070793	1.183186972	2.452448091	0.032635253	0.007141503	moderately acetylated
Pofut1	1.663871753	1.785307661	3.581388431	0.008512123	6.19574E-05	moderately acetylated
Lhx8	1.213604389	1.10024631	2.611592132	0.034824095	0.005737524	moderately acetylated
Tstd1	1.519844592	1.418275339	3.263077282	0.024105069	0.005760341	moderately acetylated
Siglech	1.053290601	1.06899109	2.261126824	0.009867305	0.000726879	moderately acetylated
Gm4246	1.424323414	1.238035854	3.053531797	0.019355512	0.003751748	moderately acetylated
Rsad2	1.083175135	1.035065904	2.318962356	0.012003163	0.001014308	moderately acetylated
Gm4910	2.086036774	2.002084358	4.460223675	0.025174811	0.02173987	moderately acetylated
Gm4458	1.925050707	1.207642722	4.112959497	0.002721892	0.000354611	moderately acetylated
4930500	2.178314409	2.076666227	4.65007825	0.030462499	0.005401846	moderately acetylated
Gm4708	1.021294839	1	2.178882555	0.002141373	4.25501E-05	moderately acetylated
Gm4494	1	1.045162594	2.128490558	0.010432067	0.00016088	moderately acetylated
Sod3	6.859891841	6.007950779	14.59945947	0.002019285	0.000252412	moderately acetylated
Hectd3	2.177112292	1.826363925	4.631989199	0.001524128	0.000188671	moderately acetylated
Gm1779	1.476306749	1.424415467	3.130484388	0.023203717	0.012244108	moderately acetylated
Krtcap3	7.838054413	5.482612951	16.6172014	0.009557847	0.001186856	moderately acetylated
Tsku	1.797969971	1.285768344	3.808486632	0.007899612	0.000947627	moderately acetylated
Gm1349	1.696368644	1.764741456	3.589673204	0.043333796	0.016057394	moderately acetylated
Gm2818	1	1	2.114933242	0.007922302	0.000365898	moderately acetylated
Il18	3.885213118	2.338227483	8.213994106	0.025780356	0.000563131	moderately acetylated
Gm3256	1.602400714	1.043128809	3.373594458	0.048633349	0.004618488	moderately acetylated
Tmem62	1.673096268	1.323416346	3.517068598	0.019592161	6.76862E-05	moderately acetylated
Gm4295	1.053133541	1.010461247	2.211279461	0.004907636	0.00017649	moderately acetylated
Gm4785	1.146332877	1	2.405032205	0.023299042	0.002717011	moderately acetylated
BC04840	1.747342908	1.786287323	3.657267515	0.003116409	0.000692044	moderately acetylated
Pax6os1	1.111353987	1	2.315717132	0.017530398	0.001236443	moderately acetylated
Sarm1	1.36030799	1.234862277	2.833939594	0.004647207	0.000173731	moderately acetylated
Palm3	1.41177391	1.303848464	2.93555367	0.01775883	0.003988847	moderately acetylated
Gm4322	1	1	2.076598817	0.025575463	0.003583504	moderately acetylated
Moxd2	1.121157328	1	2.323929546	0.0202831	0.001623919	moderately acetylated
Pde9a	1.980901784	2.008119092	4.099592091	0.007811478	0.001085889	moderately acetylated
Gm1914	2.046870411	1.217408964	4.218722166	0.014278819	0.000992934	moderately acetylated
9330198	1.578290632	1.280359982	3.245530518	0.032970952	0.009207036	moderately acetylated
Gal3st3	1.409562815	1.374172286	2.897489614	0.002952594	0.000309992	moderately acetylated
Gm4326	1.266755272	1.059941314	2.602799307	0.000143635	5.59077E-06	moderately acetylated
Tns4	1.087227649	1	2.229698158	0.000642125	5.31858E-06	moderately acetylated
Gm2881	1	1	2.049876425	0.035485492	0.006669456	moderately acetylated
4930520	14.11953929	12.33817279	28.83323929	0.002164695	0.000183609	moderately acetylated
Fibcd1	1.626111287	1.41351395	3.318060049	0.001791536	0.000176978	moderately acetylated
Sowahc	2.910620251	2.884211345	5.937651978	0.009803494	0.002736598	moderately acetylated
Gm4894	1.428223526	1.126900847	2.905347911	0.011238808	0.000263685	moderately acetylated
C130083	1	1.012155825	2.027754501	0.014775635	0.001322578	moderately acetylated
Gm15091	1.756366184	1.607175001	3.553144132	0.003557094	0.000362553	moderately acetylated

Gm3022ٹ	6.203113587	5.58704596	12.5385633	0.002309533	0.000167439	moderately acetylated
Gm2655[illegible]	1.653985869	1.568583788	3.340610468	0.008985056	0.000993183	moderately acetylated
Rlbp1	1.701842993	1.244510587	3.43428963	0.024487758	0.001375577	moderately acetylated
Gm2834[illegible]	18.18034907	14.57544755	36.64278475	0.045218184	0.011479487	moderately acetylated
Cib3	3.656288795	3.316284453	7.350383423	0.002433494	0.000583792	moderately acetylated
Gm1350[illegible]	2.048151771	1.999045127	4.113342734	0.041828002	0.000183042	moderately acetylated
Neurl1b	1.014238043	1.011213082	2.029649725	0.001251527	1.80136E-05	moderately acetylated
