# Supplementary material for: PCBP1/2 and TDP43 Function as NAT10 Adaptors to Mediate mRNA ac4C Formation in Mammalian Cells
Source: Adv Sci (Weinh). 2024 Nov 18;11(47):2400133. doi: 10.1002/advs.202400133 (PMC11653668; doi:10.1002/advs.202400133)
Supplement: Supplementary file 5 — Supporting Table [file ADVS-11-2400133-s001.docx]

**Supporting tables**

**Table S1. FPKMs of transcripts defined as ac4C(+) mRNAs in WT HEK293T cells, which the enriching levels were more than 2-folds between the acRIP and the input groups and between the acRIP and the IgG groups. (In a separated xlsx file).**

**Table S2. FPKMs of transcripts defined as ac4C(+) mRNAs in *siTDP43* HEK293T cells, which the enriching levels were more than 2-folds between the acRIP and the input groups and between the acRIP and the IgG groups. (In a separated xlsx file).**

**Table S3. FPKMs of transcripts defined as ac4C(+) mRNAs in mouse testis, which the enriching levels were more than 2-folds between the acRIP and the input groups and between the acRIP and the IgG groups. (In a separated xlsx file).**

**Table S4. siRNA sense sequence information**

| **siRNA name** | **Genes targeted** | **Species** | **Sequences (5′-3′)** |
| --- | --- | --- | --- |
| *siNC* | NC | human | UUCUCCGAACGUGUCACGUTT |
| *siPCBP1/2* | *PCBP1* | human | GAGUGUGUCAAGCAGAUUUTT |
|  | *PCBP2* |  | GGAUGCAAGAUCAAGGAAATT |
| *siTDP43* | *TDO43* | human | GGCUGGUAGAAGGAAUUCUTT |
|  |  |  | GUCACAGCGACAUAUGAUATT |
| *siNAT10* | *NAT10* | human | GGACTGCTGTAAGACTCTA |

**Table S5. Sequences of *in vitro* transcribed RNAs for acRIP and RNA pull down**

| **Name** | **Gene** | **Total length (nt)** | **Sequences (5′-3′)** |
| --- | --- | --- | --- |
| T7+β-globin | mouse β-globin | 195 | TAATACGACTCACTATAGGGGAGGAAGTAGTGAAGAGTGTTAGAGGATGCTTGTCATCACCGAAGCCTGATTCCGTAGAGCCACACCCTGGTAAGGGCCAATCTGCTCACACAGGATAGAGAGGGCAGGAGCCAGGGCAGAGCATATAAGGTGAGGTAGGATCAGTTGGATGTGGGAGTTGTAAGGTAGAATGTG |
| SP6+*RRBP1*^site-WT^ | human | 53 | ATTTAGGTGACACTATAGCTGAAGCACCCGCCAGCTCCCGCGGAGCCCTCCTC |
| SP6+*RRBP1*^site-mut^ | human | 53 | ATTTAGGTGACACTATAGCTGAAGCACCTGCGAGUAGUAGGGGAGCCCTCCTC |
| SP6+*RRBP1*^exon15-WT^ | human | 102 | ATTTAGGTGACACTATAGAATTACACCGAGTGGCTGCAGGATCTCAAAGAGAAAGGCCCCACGCTGCTGAAGCACCCGCCAGCTCCCGCGGAGCCCTCCTCG |
| SP6+*RRBP1*^exon15-mut^ | human | 102 | ATTTAGGTGACACTATAGAATTACACCGAGTGGCTGCAGGATCTCAAAGAGAAAGGCCCCACGCTGCTGAAGCACCTGCGAGUAGUAGGGGAGCCCTCCTCG |

**Table S6. Primer sequences**

| **Primer name** | **Genes targeted** | **Application** | **Sequences (5′-3′)** |
| --- | --- | --- | --- |
| hGAPDH-F | human GAPDH | Real-time PCR | GGAGCGAGATCCCTCCAAAAT |
| hGAPDH-R | human GAPDH | Real-time PCR | GGCTGTTGTCATACTTCTCATGG |
| h*PCBP1-*F | human *PCBP1* | Real-time PCR | AAAGGCGGGTGTAAGATCAAAG |
| h*PCBP1-*R | human *PCBP1* | Real-time PCR | GGCAAATCTGCTTGACACACTC |
| h*PCBP2-*F | human *PCBP2* | Real-time PCR | GCGCAGATCAAAATTGCGAAC |
| h*PCBP2-*R | human *PCBP2* | Real-time PCR | ATATTGAGCCAGGCTAATGCTG |
| h*TARDBP*-F | human *TARDBP* | Real-time PCR | GGGTAACCGAAGATGAGAACG |
| h*TARDBP*-R | human *TARDBP* | Real-time PCR | CTGGGCTGTAACCGTGGAG |
| h*NAT10*-F | human *NAT10* | Real-time PCR | ATAGCAGCCACAAACATTCGC |
| h*NAT10*-R | human *NAT10* | Real-time PCR | ACACACATGCCGAAGGTATTG |
| h18S-F | human 18S rRNA | Real-time PCR | GTAACCCGTTGAACCCCATT |
| h18S-R | human 18S rRNA | Real-time PCR | CCATCCAATCGGTAGTAGCG |
| h5.8S-F | human 5S rRNA | Real-time PCR | GCTACGCCTGTCTGAGCGTCG |
| h5.8S-R | human 5S rRNA | Real-time PCR | CGGCTCTCTCTTTCCCTCTCCG |
| mB-globin-F | mouse beta globin | Real-time PCR | TCATCACCGAAGCCTGATTCCG |
| mB-globin-R | mouse beta globin | Real-time PCR | CTACCTTACAACTCCCACATCCAACTGA |
| *Egfp*-F | *Egfp* | Real-time PCR | AGTCCGCCCTGAGCAAAGA |
| *Egfp*-R | *Egfp* | Real-time PCR | TCCAGCAGGACCATGTGATC |
| h*EEF1A1*-F | human *EEF1A1* | Real-time PCR | TGTCGTCATTGGACACGTAGA |
| h*EEF1A1*-R | human *EEF1A1* | Real-time PCR | ACGCTCAGCTTTCAGTTTATCC |
| h*RRBP1-*F | human *RRBP1* | Real-time PCR | TACGACACTCAAACCTTGGGG |
| h*RRBP1-*R | human *RRBP1* | Real-time PCR | GGTTGGCTAGGGCTTCTTCATA |
| h*RBBP6*-F | human *RBBP6* | Real-time PCR | ATACCATCCCAACAACACAAGC |
| h*RBBP6*-R | human *RBBP6* | Real-time PCR | CGCCTACGCTCCTTTTGAA |
| h*UPF3B*-F | human *UPF3B* | Real-time PCR | CCTAAGGAGAAGCGAGTAACCC |
| h*UPF3B*-R | human *UPF3B* | Real-time PCR | CCTTGTTGCGATCCTGCTTATC |
| h*FUS*-F | human *FUS* | Real-time PCR | ATGGCCTCAAACGATTATACCCA |
| h*FUS*-R | human *FUS* | Real-time PCR | GTAACTCTGCTGTCCGTAGGG |
| h*ZFP36L2*-F | human *ZFP36L2* | Real-time PCR | CAACTCCACGCGCTACAAGA |
| h*ZFP36L2*-R | human *ZFP36L2* | Real-time PCR | CACTTTTCGCCGTACTTGCAC |
| h*LAMP1*-F | human *LAMP1* | Real-time PCR | CAGATGTGTTAGTGGCACCCA |
| h*LAMP1*-R | human *LAMP1* | Real-time PCR | TTGGAAAGGTACGCCTGGATG |
| h*UPF3B*-5U-F | human *UPF3B* | Real-time PCR | TCCGGCCAAGCCGCTTCA |
| h*UPF3B*-5U-R | human *UPF3B* | Real-time PCR | CTTATCTTCCCCCTTGGAGCTGTCC |
| h*LAMP1*-5U-F | human *LAMP1* | Real-time PCR | CGCTGTCTCTAACGCCAGCCC |
| h*LAMP1*-5U-R | human *LAMP1* | Real-time PCR | CAGCAGTAGCAGCAGCAGGGGT |
| h*RRBP1*-e15-F | human *RRBP1* | Real-time PCR | TTACACCGAGTGGCTGCAGGATC |
| h*RRBP1*-e15-R | human *RRBP1* | Real-time PCR | AGGAGGGCTCCGCGGGA |
| m*Enho*-3U-F | mouse *Enho* | Real-time PCR | TGGTGCTAGAGCCAGGGCCA |
| m*Enho*-3U-R | mouse *Enho* | Real-time PCR | CAGCCTAGAGGGCCTAGAGGTGCT |
| m*Hoxd9*-F | mouse *Hoxd9* | Real-time PCR | GCCACTACGGGATTAAGCCTG |
| m*Hoxd9*-R | mouse *Hoxd9* | Real-time PCR | GCGGAGCACTCAGTCCTTT |
| m*Eef1a1*-F | mouse *Eef1a1* | Real-time PCR | CAACATCGTCGTAATCGGACA |
| m*Eef1a1*-R | mouse *Eef1a1* | Real-time PCR | GACATCTCCCTGTGGAAATTCG |
| m18S-F | mouse 18S rRNA | Real-time PCR | TGATTAAGTCCCTGCCCTTTG |
| m18S-R | mouse 18S rRNA | Real-time PCR | CTTCTCTCACCTCACTCCAGACAC |
| m*Rnf183*-F | mouse *Rnf183* | Real-time PCR | CCCCAAAGTGCTAGACTGCTG |
| m*Rnf183*-R | mouse *Rnf183* | Real-time PCR | GGGCTGGTCCTTGAGACAG |
| m*Hoxb7*-F | mouse *Hoxb7* | Real-time PCR | AAGTTCGGTTTTCGCTCCAGG |
| m*Hoxb7*-R | mouse *Hoxb7* | Real-time PCR | ACACCCCGGAGAGGTTCTG |
| m*Gapdh*-F | mouse *Gapdh* | Real-time PCR | AGGTCGGTGTGAACGGATTTG |
| m*Gapdh*-R | mouse *Gapdh* | Real-time PCR | TGACCTCAACTACATGGTCTACA |
| m*Uhrf2*-5U-F | mouse *Uhrf2* | Real-time PCR | GTTCCGGTTTCCTTCCACCGAG |
| m*Uhrf2*-5U-R | mouse *Uhrf2* | Real-time PCR | CCGGGAGGATCCGGTTCTCTG |
| Oligo(dT)_30_ | – | Reverse transcription | AAGCAGTGGTATCAACGCAGAGTACTTTTTTTTTTTTTTTTTTTTTTTTTTTTTT |

**Table S7. Antibody information**

| **Protein/Target name** | **Manufacture (catalogue number)** | **Application (working dilution)** | **Website link** |
| --- | --- | --- | --- |
| FLAG | Sigma (F3165) | western blot (1:3000) | <http://www.sigmaaldrich.com/catalog/product/sigma/f3165?lang=zh&region=CN> |
| HA | Cell Signaling Technology (3724) | western blot (1:1000) | <https://www.cellsignal.com/products/primary-antibodies/ha-tag-c29f4-rabbit-mab/3724> |
| NAT10 | Abcam (ab194297) | endo IP | ttps://www.abcam.cn/nat10-antibody-epr18663-ab194297.html |
|  | ProteinTech (13365) | western blot (1:1000) | <https://www.ptglab.com/products/NAT10-Antibody-13365-1-AP.htm> |
| IgG | Cell Signaling Technology (3900) | acRIP; endo IP | <https://www.cst-c.com.cn/products/primary-antibodies/rabbit-da1e-mab-igg-xp-isotype-control/3900?site-search-type=Products> |
| TDP43 | GeneTex (114210) | endo IP | <https://www.genetex.cn/Product/Detail/TDP43-antibody/GTX114210> |
|  | ProteinTech (10782) | western blot (1:1000) | <https://www.ptglab.com/products/TARDBP-Antibody-10782-2-AP.htm> |
|  | Biolegend (808301) | IF (1:200) | <https://www.biolegend.com/en-us/products/purified-anti-tdp43-antibody-11572> |
| PCBP1 | ProteinTech (14523) | western blot (1:1000) | <https://www.ptgcn.com/products/hnRNP-E1-Antibody-14523-1-AP.htm> |
|  | Santa Cruz (sc-137249) | IF (1:200) | <https://www.scbt.com/p/hnrnp-e1-antibody-e-2> |
| PCBP2 | ProteinTech (15070) | WB (1:1000) | <https://www.ptgcn.com/Products/PCBP2-Antibody-15070-1-AP.htm> |
| ac^4^C | Abcam (ab252215) | acRIP; dot blot (1:500) | <https://www.abcam.cn/n4-acetylcytidine-ac4c-antibody-eprnci-184-128-ab252215.html> |
| DDB1 | Abcam (ab9194) | western blot (1:2000) | [https://www.abcam.cn/products/primary-antibodies/ddb1-antibody-ab9194.html](https://www.abcam.cn/products/primary-antibodies/ddb1-antibody-ab9194.html#:~:text=Recognizes%20the%20large%20subunit%20of%20DNA%20damage-binding%20protein,is%20also%20known%20as%3A%20DDBA%3B%20XAP1%3B%20XPCE%3B%20XPE-BF%3BUV-DDB1) |
| α-tubulin | Cell Signaling Technology (2144) | western blot (1:1000) | <https://www.cellsignal.com/products/primary-antibodies/a-tubulin-antibody/2144> |
| SYCP3 | Abcam (ab97672) | IF (1:200) | <https://www.abcam.com/products/primary-antibodies/scp3-antibody-cor-10g117-ab97672.html> |
